# Self‐Healing Pd(II) Cage Metallogels for Iodine Capture in Water and Heterogeneous Photocatalysis

**DOI:** 10.1002/advs.78042

**Published:** 2026-09-29

**Authors:** Monotosh Dalapati, Raghunath Singha, Debashree Manna, Dipak Samanta

**Affiliations:** ^1^ School of Chemical Sciences National Institute of Science Education and Research (NISER) Bhubaneswar Odisha India; ^2^ Homi Bhabha National Institute Training School Complex Mumbai India; ^3^ Institute of Organic Chemistry and Biochemistry Czech Academy of Sciences Praha Czech Republic; ^4^ Center For Interdisciplinary Sciences (CIS) National Institute of Science Education and Research (NISER) An OCC of Homi Bhabha National Institute (HBNI) Bhubaneswar Odisha India

**Keywords:** dynamic adsorption, heterogenous photocatalysis, iodine, metallogel, self‐assembled cage, self‐healing

## Abstract

Adaptive multifunctional systems that integrate responsiveness, environmental remediation, and catalysis within a single platform remain exceptionally rare. Herein, two carbazole‐derived metal‐organic cages (MOCs) were constructed through coordination‐driven self‐assembly and subsequently transformed into fibrous metallogels (**MOC1G** and **MOC2G**) in mixed DMSO/H_2_O media through cooperative *π−π* stacking and dynamic supramolecular interactions. The resulting metallogels display reversible gel−sol transitions, interconnected nanofibrous architectures, and pronounced viscoelastic behavior. Rheological analyses revealed dominant elastic characteristics with rapid recovery after large mechanical deformation. Remarkably, the metallogels exhibit autonomous self‐healing without external stimuli, restoring their structural integrity within ∼18 min for **MOC1G** and ∼14 min for **MOC2G** after mechanical damage. Temperature‐dependent DLS and DSC studies further confirmed reversible thermally driven assembly−disassembly processes. Beyond their adaptive soft‐matter properties, the discrete MOCs demonstrated highly efficient capture of iodine species from aqueous media, achieving uptake capacities up to 3.01 g g^‒1^ together with excellent selectivity and fast adsorption kinetics under both static and continuous‐flow conditions. In addition, the cages function as visible‐light‐responsive heterogeneous photocatalysts for aerobic sulfide oxidation, affording sulfoxides under mild conditions. This work establishes the first example of cage‐based metallogels that exhibit adaptive and autonomous self‐healing behavior while integrating environmental remediation and photocatalytic functionality within a single supramolecular platform.

## Graphical abstract

Two carbazole‐derived metal‐organic cages hierarchically self‐assemble into supramolecular metallogels that integrate autonomous self‐healing, reversible stimuli responsiveness, high‐capacity aqueous iodine capture, and visible‐light‐driven photocatalytic sulfide oxidation within a single multifunctional platform.

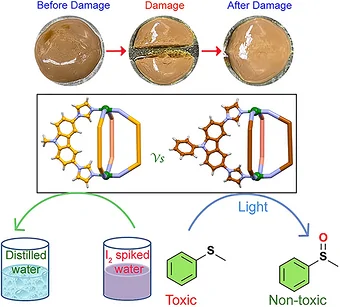

## 1 Introduction

Adaptive materials that can recover from mechanical damage while responding to external stimuli are of considerable interest in soft‐matter science [1, 2]. Biological tissues provide a compelling model because they combine self‐repair, environmental responsiveness, and mechanical integrity within dynamic networks [3, 4, 5, 6]. Inspired by these features, synthetic self‐healing gels have been developed using reversible dynamic covalent bonds and noncovalent interactions, allowing damaged networks to reorganize and recover their structure and function [7, 8, 9, 10]. Incorporating thermoresponsiveness into such systems can further regulate network assembly and disassembly, enabling reversible gel−sol transitions and stimulus‐assisted healing [11, 12, 13]. Nevertheless, simultaneously achieving rapid self‐healing, reversible thermal responsiveness, and robust mechanical properties in a simple and modular material remains challenging [14]. Supramolecular gels are particularly attractive in this regard because their networks are sustained by dynamic interactions such as hydrogen bonding, *π−π* stacking, hydrophobic interactions, van der Waals forces, and metal−ligand coordination [15, 16, 17, 18]. The reversibility of these interactions enables structural adaptation while providing access to stimuli‐responsive behavior and diverse functional properties [17, 19, 20, 21, 22].

Among these systems, metallogels formed through coordination‐driven assembly offer an additional level of molecular control because metal−ligand coordination can define both the primary molecular architecture and the higher‐order network [23, 24]. Metal−organic cages (MOCs) are especially appealing building blocks because they combine well‐defined coordination geometries with enclosed cavities for molecular recognition and host−guest chemistry [25]. Their cavities have been extensively harnessed for the encapsulation of anions [26], neutral molecules [27], radical initiators [28], and pharmaceutical compounds [29], enabling advances in molecular recognition, photophysics, gas adsorption, and catalysis [30, 31, 32, 33, 34]. However, most MOCs are studied as discrete molecular hosts, whereas their use as molecular building blocks for adaptive supramolecular gels remains comparatively underexplored [35, 36, 37]. In particular, Pd_2_L_4_ cages provide a useful platform for this purpose because their well‐defined cationic cavities can be combined with ligand‐derived functionality and their molecular surfaces can participate in intermolecular interactions. Thus, converting such discrete cages into higher‐order gels offers an opportunity to couple molecular‐scale host–guest properties with macroscopic self‐healing and stimuli‐responsive behavior.

This molecular‐to‐material connection is also attractive for environmental applications [38]. Iodine is widely used across medical, industrial, agricultural, and food‐processing sectors [39], yet radioactive isotopes such as ^129^I and ^131^I, produced during nuclear fuel reprocessing, create serious environmental and health hazards. The extremely long half‐life of ^129^I (∼1.6 × 10^7^ years) and the high radiotoxicity of ^131^I make their removal from water particularly important [40, 41]. Various porous materials−including MOFs, COFs, HOFs, and porous organic polymers−have been explored for iodine capture [42, 43, 44, 45, 46, 47]. However, many of these systems demonstrate high adsorption capacities only under static conditions, whereas practical water‐treatment processes require efficient iodine removal under continuous flow and at low concentrations [48, 49]. Materials capable of capturing iodine efficiently under such realistic conditions remain limited. The cationic cavities and π‐rich surfaces of MOCs provide a potentially useful molecular environment for stabilizing iodine species through electrostatic, confinement, and π‐interaction/charge‐transfer effects.

The same cage architecture can, in principle, provide a second function through its photophysical properties [50, 51]. Incorporation of heavy metal centers such as Pd(II) promotes intersystem crossing (ISC) and facilitates the generation of reactive oxygen species (ROS) [52, 53, 54], while carbazole‐based chromophores are known to stabilize long‐lived triplet excited states required for ROS formation [55, 56]. These features make metal‐organic cages attractive candidates for photocatalytic aerobic oxidation reactions, such as the conversion of sulfides to sulfoxides an important transformation in organic synthesis and pharmaceutical chemistry [53, 57, 58]. Importantly, the possibility of combining host−guest recognition and photocatalysis within the same molecular cage provides a direct design route toward multifunctional supramolecular materials. Despite advances in cage‐based adsorption, photocatalysis, and adaptive gels as separate fields, systems that integrate cage‐based hierarchical assembly, self‐healing behavior, aqueous iodine capture, and photocatalytic activity within one platform remain rare.

Herein, we report a multifunctional metal−organic cage platform that integrates thermoresponsive metallogel formation, efficient iodine capture from aqueous environments, and heterogeneous photocatalysis within a single architecture. Two carbazole‐derived Pd_2_L_4_ cages, **MOC1** and **MOC2**, were constructed through coordination‐driven self‐assembly and subsequently organized into fibrous metallogels in a DMSO/H_2_O medium. The resulting gels (**MOC1G** and **MOC2G**) exhibit reversible gel−sol transitions and autonomous self‐healing, demonstrating how discrete cage units can be translated into adaptive macroscopic materials. The molecular cavities of the corresponding MOCs enable efficient capture of iodine species from aqueous media through π−iodine, and electrostatic interactions, exhibiting rapid adsorption kinetics (0.5 g mg^−1^ min^−1^), excellent dynamic flow‐through performance with breakthrough volumes reaching 8.2 L g^−1^, and near‐quantitative iodine removal from dilute aqueous solutions, reducing residual iodine concentrations to the low ppb range. In addition, the synergistic combination of Pd(II) centers and carbazole chromophores enables efficient ROS‐mediated aerobic oxidation of sulfides under blue light, affording high conversions (≥70%) and excellent selectivity (∼97%) toward sulfoxides under ambient conditions while completely suppressing sulfone formation. Comparison of **MOC1** and **MOC2** further allows the influence of ligand substitution on assembly, iodine binding, and photocatalytic performance to be examined within a common structural framework. Thus, this work establishes a unified cage‐based strategy that links molecular architecture to hierarchical soft‐matter assembly, environmental remediation, and photocatalysis.

## 2 Results and Discussion

### 2.1 Synthesis and Characterization

The carbazole‐derived ligands **L1** and **L2** were prepared through Ullmann coupling reactions between the corresponding dibromo‐carbazole precursors bearing methyl or phenyl substituents and imidazole, affording the desired ligands in good yields (Schemes S1–S6). The synthesized ligands were comprehensively characterized by ^1^H and ^13^C NMR spectroscopy together with high‐resolution mass spectrometry (HRMS) (Figures S1–S14). Subsequent coordination‐driven self‐assembly of ligands **L1** and **L2** with Pd(NO_3_)_2_ in DMSO using a 2:1 ligand‐to‐metal stoichiometry at room temperature furnished the corresponding metal−organic cages **MOC1** and **MOC2**, respectively (Scheme 1). The assembly process was completed within 1 h, producing clear brown solutions, and the resulting cages were isolated as white solids upon addition of excess ethyl acetate. The formation of **MOC1** and **MOC2** was confirmed by NMR spectroscopy, ESI‐MS, and single‐crystal X‐ray diffraction analysis (Figure 1, Figures S15–S29). The formation of the Pd(II)‐directed self‐assembled cages was verified by ^1^H NMR spectroscopy, which revealed significant downfield shifts of the imidazole resonances upon metal coordination. In the case of **MOC1**, the H_a_ resonance shifted from 8.27 to 8.97 ppm, while the H_c_ resonance moved from 7.79 to 8.05 ppm (Figure 1a). Likewise, **MOC2** displayed pronounced downfield shifts, with the H_a_ proton moving from 8.26 to 9.60 ppm and the H_c_ proton from 7.76 to 8.13 ppm (Figures S12–S23), consistent with successful coordination to Pd(II).

**SCHEME 1:**
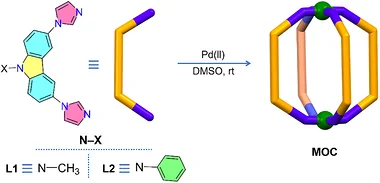
Illustration of the Pd(II)‐directed coordination‐driven self‐assembly of ligands **L1** and **L2** into discrete metal‐organic cage architectures (**MOC1** and **MOC2**).

**FIGURE 1:**
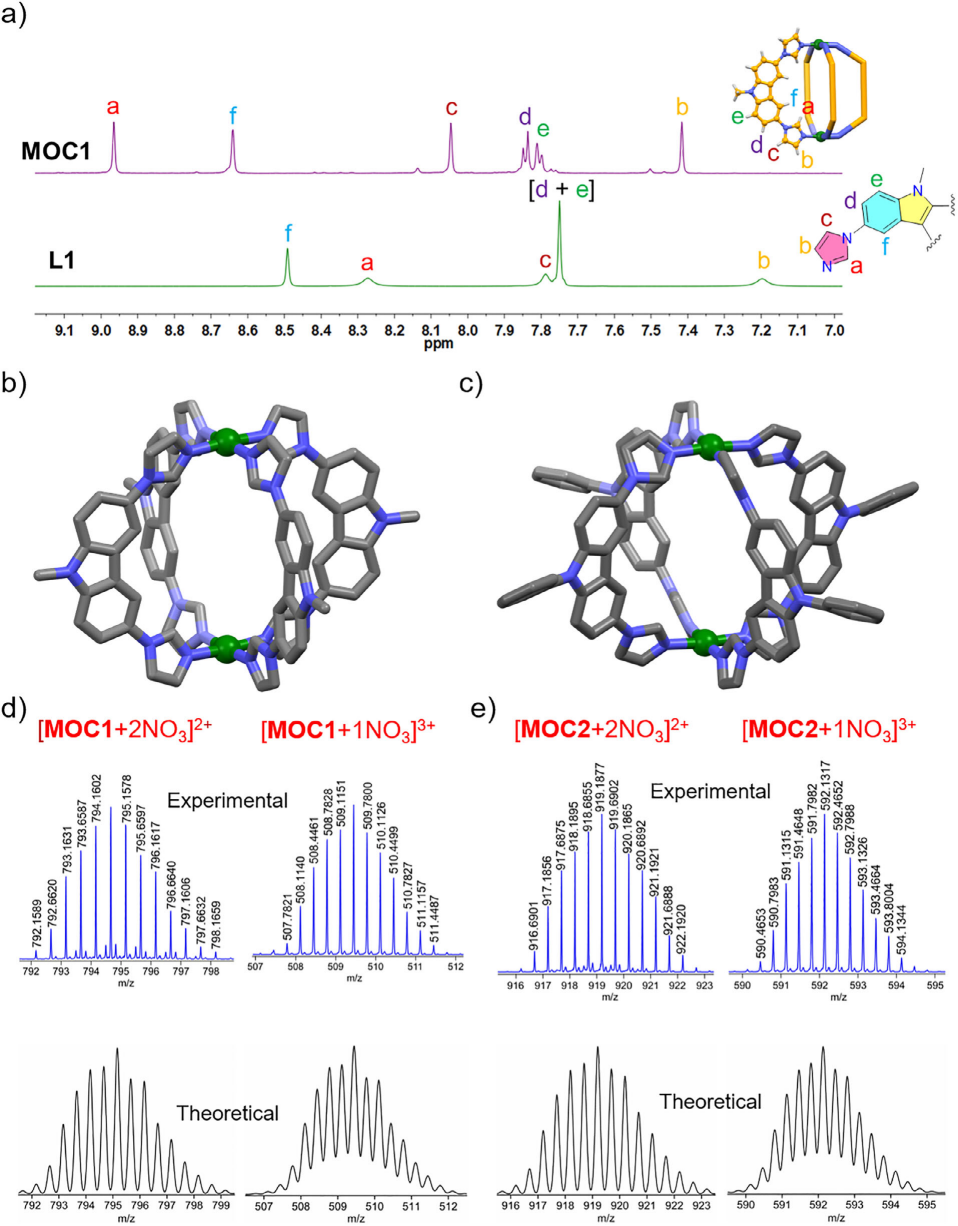
(a) Partial ^1^H NMR spectra (700 MHz, DMSO‐*d_6_*, 298 K) of ligand **L1** and the corresponding self‐assembled cage **MOC1**. Single‐crystal X‒ray diffraction structures of (b) **MOC1** and (c) **MOC2** (counteranions, hydrogen atoms, and solvent molecules omitted for clarity; Pd, green; C, dark grey; N, blue). Experimental and simulated ESI‐MS isotopic distributions of representative cationic fragments for (d) **MOC1** and (e) **MOC2**.

Detailed signal assignments were established using two‐dimensional ^1^H‐^1^H COSY and NOESY experiments (Figures S17–S20 and S25–S27). Furthermore, ^1^H DOSY NMR measurements confirmed the formation of discrete and monodisperse cage species, as all proton signals exhibited identical diffusion coefficients. The measured diffusion constants of 7.85 × 10^−^^11^ m^2^ s^−^^1^ for **MOC1** and 6.55 × 10^−^^11^ m^2^ s^−^^1^ for **MOC2** correspond to hydrodynamic radii of 14.0 and 16.78 Å, respectively, supporting the formation of well‒defined cage architectures (Figures S21–S28). ESI‒MS further confirmed the successful formation of the cages by revealing characteristic multiply charged species corresponding to the assembled complexes. For **MOC1**, prominent peaks were observed at m/z = 795.1578 and 509.4470, assignable to [**MOC1** + 2NO_3_]^2+^ and [**MOC1** + 1NO_3_]^3+^, respectively (Figure 1d, Figure S22). Similarly, **MOC2** displayed signals at m/z = 919.1877 and 592.1317 corresponding to [**MOC2** + 2NO_3_]^2+^ and [**MOC2** + 1NO_3_]^3+^, respectively (Figure 1e, Figure S29). In both cases, the experimentally observed isotopic distributions showed excellent agreement with the simulated patterns, confirming the formation of discrete Pd_2_L_4_ cage architectures.

Conclusive structural confirmation of the assemblies was obtained by single‐crystal X‒ray diffraction analysis. Single crystals suitable for X‒ray measurements were grown by slow vapor diffusion of 1,4‐dioxane and THF into DMSO solutions of **MOC1** and **MOC2**, respectively (Figure 1b,c). **MOC1** crystallizes in the monoclinic space group *C*2/c and features a dinuclear lantern‐type [Pd_2_L_4_] architecture in which each Pd(II) center adopts a square‐planar coordination geometry defined by four imidazole nitrogen donors from four independent ligands. The asymmetric unit consists of one Pd(II) ion and two ligand fragments, with a Pd···Pd separation of 9.985 Å (Figure 1b, Table S1). In contrast, **MOC2** crystallizes in the triclinic space group $P\bar{1\;}$ and displays a closely related [Pd_2_L_4_] cage structure with an intermetallic Pd···Pd distance of 9.941 Å (Figure 1c, Table S2).

After confirming the successful formation of the cages, their thermal behavior, porosity, and solid‐state characteristics were systematically investigated. Thermogravimetric analysis (TGA) performed under a nitrogen atmosphere demonstrated that both cages remain thermally stable up to ~210 ^ο^C, with the initial weight loss corresponding to the release of encapsulated solvent molecules (Figure S53). The porosity of the materials was further evaluated by N_2_ adsorption−desorption measurements at 77 K following activation at 110 ^ο^C under vacuum for 12 h to remove residual solvent molecules completely. Brunauer−Emmett−Teller (BET) analysis revealed surface areas of 42.30 m^2^ g^−1^ for **MOC1** and 74.44 m^2^ g^−1^ for **MOC2**, with pore diameters of 1.52 and 1.70 nm, respectively, consistent with the intrinsic cavity dimensions of the cage architectures (Figures S30 and S31, Table S3). Powder X‐ray diffraction (PXRD) analysis indicated that both materials are predominantly amorphous in the solid state, as evidenced by their broad and weak diffraction patterns (Figure S99). Scanning electron microscopy (SEM) imaging revealed distinct morphologies for the two cages, where **MOC1** exhibited aggregated block‐like structures, while **MOC2** formed interconnected fibrous nanostructures (Figure S32). Furthermore, energy‐dispersive X‐ray spectroscopy (EDS) elemental mapping confirmed the uniform spatial distribution of all constituent elements (e.g., Pd, C, N and O) throughout the materials (Figures S33 and S34).

Having established the formation of discrete and structurally well‐defined Pd_2_L_4_ cages, we next considered how these molecular architectures could be translated into higher‐order soft materials. Importantly, the cage design incorporates two complementary structural features: the rigid, π‐conjugated carbazole surfaces provide an extended platform for intermolecular aromatic interactions, while the discrete Pd(II)‐directed architecture maintains a defined molecular cavity and dynamic coordination environment. We therefore anticipated that these cages could serve not only as isolated host molecules but also as molecular building blocks for hierarchical supramolecular assembly. In an appropriate solvent environment, cooperative π−π stacking, hydrophobic, electrostatic, and other reversible noncovalent interactions could organize the discrete cages into extended one‐dimensional and three‐dimensional networks. This molecular‐to‐macroscopic hierarchy provides a direct link between the structural precision of the individual cages and the adaptive properties of the resulting soft materials. We therefore investigated whether **MOC1** and **MOC2** could undergo further organization into self‐supporting metallogels and whether the molecular differences between the two cages would influence their resulting network structures and mechanical properties.

### 2.2 Gel Synthesis, Characterization, and Self‐Healing

Although discrete metal−organic cages (MOCs) are widely recognized for their defined cavities and host−guest capabilities, their utilization as building blocks for supramolecular metallogels remains comparatively unexplored. Motivated by the strong aggregation tendency of carbazole‐based systems [59, 60], we envisioned that the synthesized MOCs could further organize through hierarchical self‒assembly to generate extended gel networks. In appropriate solvent environments, cooperative noncovalent interactions−primarily *π−π* stacking and hydrophobic interactions were expected to promote intermolecular organization and ultimately driving their integration into extended, three‐dimensional gel frameworks. Consistent with this hypothesis, addition of an aqueous Pd(NO_3_)_2_ solution to DMSO solutions of ligands **L1** or **L2** in a 1:2 metal‐to‐ligand ratio, followed by gentle shaking and standing for 2 h, resulted in the formation of stable light‐brown supramolecular metallogels, **MOC1G** and **MOC2G** in a DMSO/H_2_O mixed solvent system (3:2, v/v) (Figure 2a). The critical gelation concentration (CGC) was determined to be 2.2% (w/v), and successful gel formation was verified by the vial inversion method for both the MOCGs. Gelation was found to be highly dependent on the solvent composition, while the DMSO/H_2_O mixture (3:2) was optimal, other solvents such as DMF, methanol, and acetonitrile failed to produce gels, highlighting the crucial influence of solvent polarity and solvation effects on the self‒assembly process (Table S4). The gelation process is attributed to the hierarchical aggregation of the discrete MOC units through multiple cooperative supramolecular interactions. In particular, the π−conjugated carbazole moieties within the cage framework facilitate strong intermolecular *π−π* stacking interactions, which are further reinforced by hydrogen‒bonding interactions involving solvent molecules and counter anions, together with anion‐mediated electrostatic interactions and dynamic solvent−ligand exchange processes [36, 37, 61]. The synergistic interplay of these noncovalent interactions drives the organization of discrete **MOC1** and **MOC2** cages into an interconnected three‐dimensional fibrous network, ultimately producing self‐supporting supramolecular metallogels. We performed control experiments using the pure carbazole‐based ligands in the absence of Pd(II) metal precursors, as well as experiments using the Pd(II) metal acceptor alone under otherwise identical conditions. In both cases, no gel formation was observed, demonstrating that neither the ligand nor the metal precursor individually is capable of generating the observed gel network (Figure S35).

**FIGURE 2:**
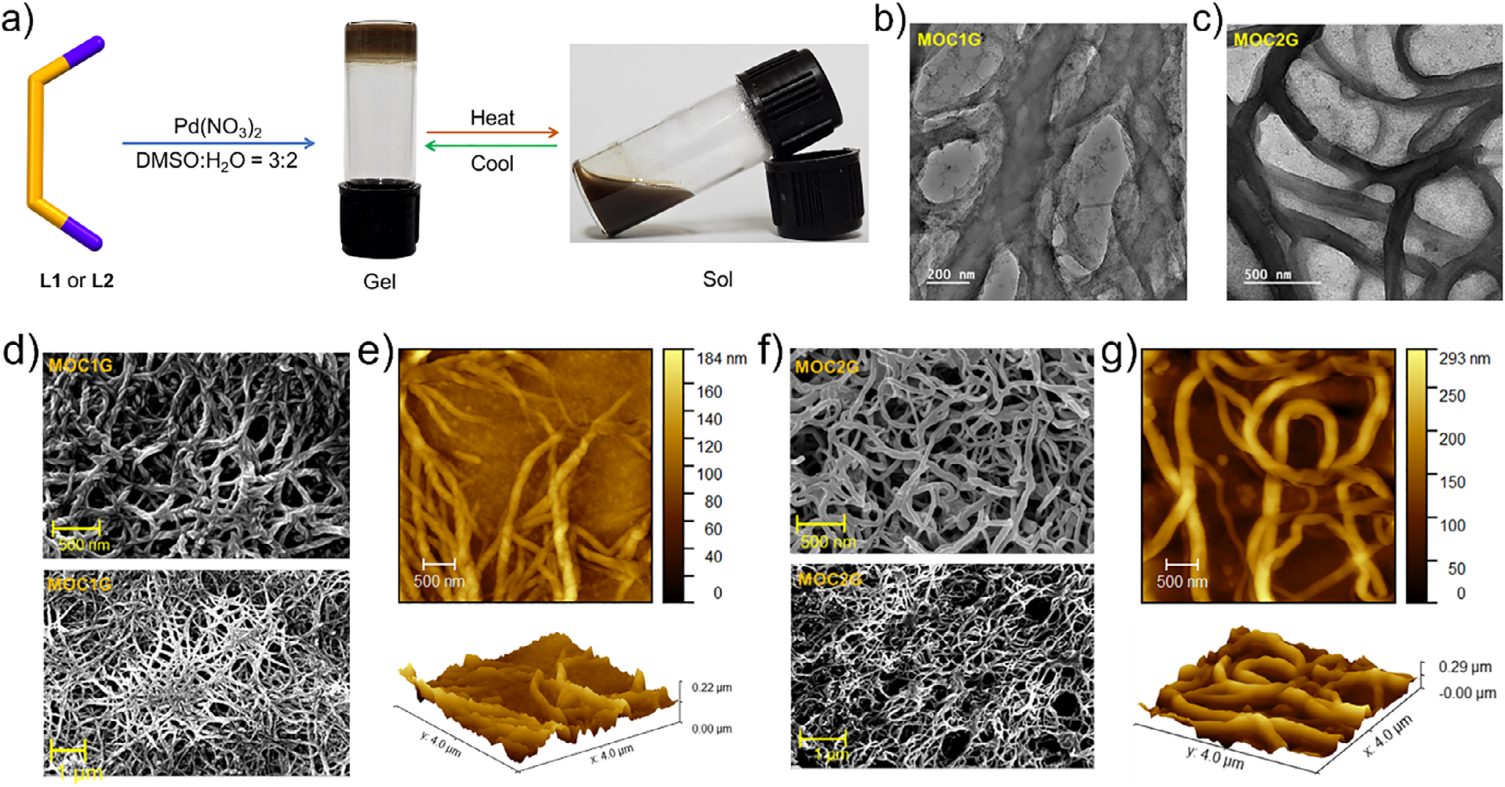
(a) Schematic illustration of supramolecular metallogel formation through Pd(NO_3_)_2_‒mediated complexation of ligands **L1** and **L2**, together with the reversible thermally induced sol‐gel transition. TEM images of (b) **MOC1G** and (c) **MOC2G** showing the fibrous nanostructures of the metallogels. FE‒SEM images of (d) **MOC1G** and (f) **MOC2G** highlighting their interconnected gel‐network morphologies. AFM topographic images of (e) **MOC1G** and (g) **MOC2G**.

The supramolecular morphologies of **MOC1G** and **MOC2G**, originating from cage‐based hierarchical self‐assembly were comprehensively examined by FE‒SEM, TEM, and AFM analyses. FE‒SEM images show that both metallogels consist of interconnected three‐dimensional fibrous architectures characteristic of supramolecular gel networks (Figure 2d,f). **MOC1G** forms an entangled network of relatively uniform nanofibers with an average diameter of 127 ± 14 nm, generating an open and continuous framework capable of supporting gelation (Figure 2d, Figure S49a,b). In contrast, **MOC2G** exhibits comparatively thicker worm‐like fibers with an average diameter of 108 ± 13 nm arranged in a denser and more compact network, likely arising from enhanced π‐stacking interactions and steric effects associated with the phenyl substituents (Figure 2f, Figure S49c,d). TEM analysis further confirmed the presence of elongated and interconnected fibrous assemblies in both metallogels (Figure 2b,c). Consistent with these observations, AFM topographic and height analyses revealed narrower height distributions for **MOC1G** (103 ± 43 nm), whereas **MOC2G** displayed significantly thicker fibers and larger height variations (159 ± 35 nm), indicative of a more tightly packed network structure (Figure 2e,g, Figure S50). Three‐dimensional AFM reconstructions clearly visualized the cross‐linked fibrous morphology and hierarchical nanoscale organization of the metallogels. The distinct morphological differences between **MOC1G** and **MOC2G** are attributed to the methyl versus phenyl substitution on the carbazole scaffold, which modulates intermolecular packing, fiber bundling, and overall network organization. Collectively, these microscopic studies demonstrate that carbazole‐based discrete MOCs undergo hierarchical self‐assembly into adaptive nanofibrous metallogels with the observed morphological distinctions. In addition, EDS elemental mapping confirmed the uniform distribution of Pd, C, N, and O throughout the metallogel matrices (Figures S45 and S46).

Further evidence for the metallogel properties was obtained from FT‒IR spectroscopy. The stacked FT‐IR spectra of ligands **L1** and **L2** and their corresponding metallogels show that the characteristic aromatic C─H and C═C vibrational bands remain largely preserved after gel formation. In contrast, noticeable shifts were observed for the imidazole‐associated vibrations upon coordination to Pd(II). The C═N stretching bands shifted from 1653.17 cm^−1^ (**L1**) and 1657.55 cm^−1^ (**L2**) to 1652.69 cm^−1^ (**MOC1G**) and 1655.58 cm^−1^ (**MOC2G**), respectively, while the C−N stretching vibrations moved from 1308.46 to 1315.50 cm^−1^ for **MOC1G** and from 1309.00 to 1312.57 cm^−1^ for **MOC2G** (Figures S44–S51 and S52). These spectral shifts provide strong evidence for coordination of the imidazole nitrogen donors to the Pd centers and confirm the crucial role of metal−ligand interactions in stabilizing the supramolecular metallogel framework [37] We also performed additional spectroscopic investigations, including ESI‐MS, ^1^H‐DOSY NMR, and variable‐temperature ^1^H NMR spectroscopy on the metallogel material after dissolution (Figures S36‒S43). These additional data provide consistent evidence that the Pd_2_L_4_ cage units remain structurally intact upon incorporation into the gel network. In particular, the ESI‐MS and DOSY NMR results support the persistence of the discrete cage species, while the variable‐temperature ^1^H NMR measurements further confirm the stability of the cage architecture under the investigated conditions. These results support our proposed hierarchical assembly model in which the preformed Pd_2_L_4_ cages act as discrete molecular nodes/building units that further organize through noncovalent interactions to generate the fibrous metallogel network, rather than undergoing decomposition or extensive structural reorganization during gel formation.

The mechanical behavior and gelation properties of the metallogels were systematically analyzed by oscillatory rheology, including amplitude and frequency sweep measurements, to evaluate their viscoelasticity, structural integrity, and mechanical resilience [21, 61]. Amplitude sweep experiments were performed over a strain range of 0.01%–100% at a fixed angular frequency of 10 rad s^−1^ to probe the deformation tolerance of the supramolecular networks (Figure 3a, Figure S54). Within the linear viscoelastic region (LVR), both the storage modulus (G′) and loss modulus (G″) remained nearly invariant up to ~1% strain for both metallogels, indicating that the gel structures remain intact under small deformations. Upon increasing the strain, G′ decreased significantly while G″ initially remained relatively stable before declining, reflecting progressive disruption of the gel network. The crossover points, where G″ exceeded G′ and the gels transitioned from a solid‐like to a liquid‐like state, occurred at 31.6% strain for **MOC1G** and 46.5% strain for **MOC2G**, indicating distinct mechanical responses of the two gel systems and highlighting the enhanced elasticity of **MOC2G**. The viscoelastic properties were further investigated through frequency sweep measurements conducted within the LVR at a constant strain well below the critical strain (0.1%) (Figure 3b, Figure S55). In both metallogels, G′ remained consistently higher than G″ across the examined frequency range, confirming the predominately elastic and solid‐like nature of the supramolecular networks. Notably, **MOC2G** exhibited higher storage modulus values than **MOC1G**, indicating superior mechanical strength and rigidity. These rheological results demonstrate that both materials behave as viscoelastic supramolecular solids stabilized by reversible noncovalent interactions. The enhanced mechanical performance of **MOC2G** is likely associated with stronger intermolecular π–π interactions induced by the phenyl‒substituted carbazole units relative to the methyl‐substituted analogue.

**FIGURE 3:**
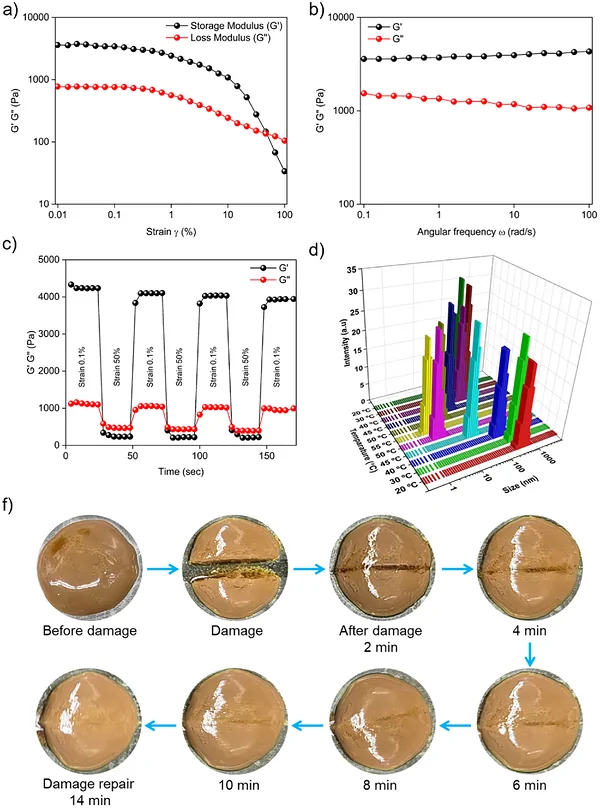
(a) Amplitude sweep rheological analysis of **MOC2G**, showing the strain‐dependent viscoelastic behavior. (b) Frequency‒dependent viscoelastic moduli (storage modulus, G′, and loss modulus, G′′) of **MOC2G** measured at a constant strain of 0.1 %. (c) Thixotropic behavior of **MOC2G** under a continuous step‐strain cycle (0.1 % → 50 % → 0.1 % strain). (d) DLS analysis of **MOC2G** at varying temperatures. (e) Photographs demonstrating the autonomous self‐healing behavior of **MOC2G**, showing spontaneous restoration of the gel within 14 min after mechanical damage.

Both metallogels also displayed pronounced thermoresponsive behavior characterized by reversible gel‐sol transitions. Visual inspections revealed that mild heating converted the self‐supporting gels into free‐flowing sols, whereas cooling restored the gel state together with its original mechanical integrity (Figure 2a). To further investigate this thermally responsive behavior, differential scanning calorimetry (DSC) and temperature‒dependent dynamic light scattering (DLS) studies were carried out. DSC thermograms of **MOC1G** and **MOC2G** exhibited distinct endothermic transitions corresponding to thermally induced gel‒to‒sol transformations (Figure S47) [62, 63]. **MOC1G** showed a transition in the range of ∼52 °C‒55 °C, while **MOC2G** exhibited a slightly higher transition temperature (∼54 °C‒60°C), suggesting enhanced thermal stability and stronger supramolecular interactions in the latter system. Temperature‐dependent DLS measurements further provided insight into the reversible nanoscale assembly behavior of the metallogels (Figure 3d, Figure S48). At lower temperatures (20 °C–30 °C), the number‐average size distributions were dominated by large aggregates with hydrodynamic diameters of ~300–500 nm, consistent with the presence of extended fibrous assemblies associated with the gel state. Upon heating to ∼45 °C‒55 °C, these hierarchical aggregates dissociated into smaller species, resulting in hydrodynamic diameters of ~30 nm for **MOC1G** and ~10 nm for **MOC2G**, characteristic of the sol state. Cooling the solutions restored the larger aggregate populations, with the hydrodynamic diameters returning to ∼400 nm at 20 °C, thereby confirming the reversible reconstruction of the supramolecular gel network (Figure 3d, Figure S48) [64, 65]. Overall, the DSC, and DLS studies establish that the metallogels undergo a fully reversible, thermally controlled sol‒gel transition driven by dynamic supramolecular interactions.

The metal–organic cage‐based gels display remarkable macroscopic self‐healing behavior, highlighting the dynamic and reversible nature of the underlying supramolecular network (Figure 3e, Figure S57). In the undisturbed state, the gels exist as homogeneous, self‒supporting monoliths, however, mechanical cutting generates a visible fracture, disrupting the continuous network and separating the material into two independent pieces. Upon bringing the fractured surfaces back into contact, the damaged interface gradually disappears without requiring any external stimulus, demonstrating spontaneous autonomous healing. Complete recovery of the gel network occurs within ~18 min for **MOC1G** and ~14 min for **MOC2G**, after which the gels fully regain their original shape and mechanical integrity (Figure 3e, Figure S57) [21, 66]. The self‒healing performance was further investigated by thixotropic rheological measurements using alternating strain cycles (0.1% → 50% → 0.1%) (Figure 3c, Figure S56). Application of high oscillatory strain (50%) disrupted the gel network, resulting in an immediate transition from gel to sol state, as indicated by the crossover of the storage and loss moduli (G″ > G′). Upon restoring the strain to 0.1%, the storage modulus rapidly recovered and again exceeded the loss modulus, with both G′ and G″ values returning close to their original magnitudes [21, 67]. This rapid and reproducible recovery demonstrates the remarkable ability of the metallogel to reconstruct its supramolecular network following mechanical disruption, resulting in excellent self‒healing efficiency and mechanical resilience. Additionally, we performed multiple self‐healing cycles and quantitatively evaluated the mechanical recovery of the gels. Specifically, frequency and amplitude‐sweep rheological measurements were carried out over three consecutive damage−healing cycles to compare the mechanical properties of the pristine and healed gels (Figures S58‒S61). The corresponding storage modulus (G′) and loss modulus (G″) were monitored before and after each healing cycle, and the quantitative recovery ratios were determined. In both cases, the gels exhibited excellent recovery of G′ and G″, demonstrating that the healing process restores not only the macroscopic integrity of the gel but also its mechanical properties to a high degree (Figures S58‒S61 and Table S5). Although self‐healing gels derived from polymers and low‐molecular‐weight gelators have been extensively investigated, the emergence of autonomous self‐healing behavior in discrete metal‐organic cage‐based architectures remains largely unexplored. Notably, this represents the first example of a metal‐organic cage system exhibiting adaptive autonomous self‐healing behavior, highlighting its promise for reusable and stimuli‒responsive soft‐matter applications.

The hierarchical assembly studies demonstrate that the discrete MOCs retain their molecular identity while gaining new collective properties through reversible intermolecular organization. Importantly, this hierarchical assembly does not eliminate the intrinsic molecular functionality of the cages; rather, it provides a route to integrate their well‐defined cavities and π‐rich surfaces into a macroscopic, recoverable material platform. This feature is particularly relevant for molecular recognition and environmental remediation, where the combination of accessible host‐guest sites and a robust, reusable material architecture is highly desirable. The cationic Pd_2_L_4_ cages contain electron‐rich carbazole surfaces within and around their confined cavities, providing complementary π‐rich and electrostatic environments for interaction with iodine species. We therefore reasoned that the same molecular characteristics responsible for the host‐guest character of the discrete cages could be exploited independently of the macroscopic gel network to achieve efficient iodine sequestration from water. Thus, having established the hierarchical assembly and adaptive behavior of the cage‐based soft materials, we next examined the intrinsic iodine‐binding capability of the discrete MOCs and evaluated whether their molecular recognition properties could translate into efficient and recyclable iodine‐removal platforms.

### 2.3 Iodine Adsorption From Aqueous Media

Radioactive iodine released during nuclear fuel reprocessing and energy generation represents a major environmental hazard, especially when iodine species enter industrial wastewater streams or natural water reservoirs [68]. The development of materials capable of rapidly removing iodine from aqueous environments is therefore of considerable importance for both environmental remediation and emergency response applications. Although a variety of porous adsorbents have been reported for iodine capture, most are primarily effective for iodine vapor adsorption or for uptake from nonpolar organic solvents such as hexane and cyclohexane [69]. In contrast, iodine sequestration from water remains particularly challenging because of the low solubility of iodine, the coexistence of multiple iodine species, and the presence of competing ions in aqueous media. To overcome these limitations, we envisioned that cationic metal–organic cages (MOCs) incorporating a high density of electron‒rich carbazole units could provide an effective platform for iodine capture through cooperative host‐guest and electrostatic interactions. The π‐electron‐rich carbazole framework together with the positively charged cage environment was expected to promote strong binding toward various aqueous iodine species, including I_2_ and I_3_^−^, thereby enabling efficient iodine sequestration under realistic aqueous conditions. To evaluate this hypothesis, adsorption studies were carried out using aqueous solutions of I_2_ and I_3_^−^ as separate source phases.

The iodine adsorption performance of the cages was initially evaluated using aqueous I_2_ solutions. Upon addition of 2 mg of **MOC1** or **MOC2** to 3 mL of a 1.2 mm I_2_ solution, the characteristic yellow coloration of iodine rapidly disappeared, producing a colorless solution within ~4 min, indicative of fast iodine uptake by both cages (Figures S63–S67). Kinetic analysis of the adsorption data revealed excellent agreement with a pseudo‒second‒order model (R^2^> 0.99), suggesting that the adsorption process is predominantly governed by chemisorption interactions (Figure S68). The calculated adsorption rate constants were 0.1463 g mg^−1^ min^−1^ for **MOC1** and 0.2198 g mg^−1^ min^−1^ for **MOC2**, with both systems achieving iodine removal efficiencies exceeding 99% (Figure S68 and Table S6). The equilibrium adsorption behavior was further analyzed using Langmuir and Freundlich isotherm models. The adsorption data showed a significantly better fit with the Freundlich model (R^2^ = 0.93−0.98) than with the Langmuir model (R^2^ = 0.80−0.82), indicating a heterogeneous multilayer adsorption process on the cage surfaces (Figures S71 and S72, Table S7). The maximum iodine uptake capacities were determined to be 2.07 g g^−1^ for **MOC1** and 2.45 g g^−1^ for **MOC2** (Figure 4e, Figure S69), highlighting the enhanced adsorption capability of **MOC2**. To examine adsorption selectivity under competitive conditions, iodine uptake studies were performed in the presence of excess interfering anions, including F^−^, Cl^−^, Br^−^, HCO_3_^−^, NO_3_^−^, and SO_4_^2−^. Remarkably, even in the presence of 100 equivalents of competing anions, iodine removal efficiencies remained exceptionally high at 94% for **MOC1** and 95% for **MOC2** (Figure 4d). The corresponding distribution coefficients (*K*_d_) remained on the order of 10^4^ mL g^−1^ even in the presence of equimolar competing anions, demonstrating the strong binding affinity and excellent selectivity of the cages toward iodine species (Figure S70) [70]. Considering the environmental relevance of radioactive iodine contamination in marine systems during nuclear accidents, the adsorption performance of the cages was also evaluated using iodine‐spiked seawater samples. Under these conditions, **MOC1** and **MOC2** exhibited uptake capacities of 1.67 and 1.98 g g^−1^, respectively, reaching equilibrium after 18 and 24 h (Figure 4c). These results demonstrate that the cages maintain high iodine adsorption efficiency even under chemically complex and high salinity conditions, highlighting their potential applicability for practical environmental remediation.

**FIGURE 4:**
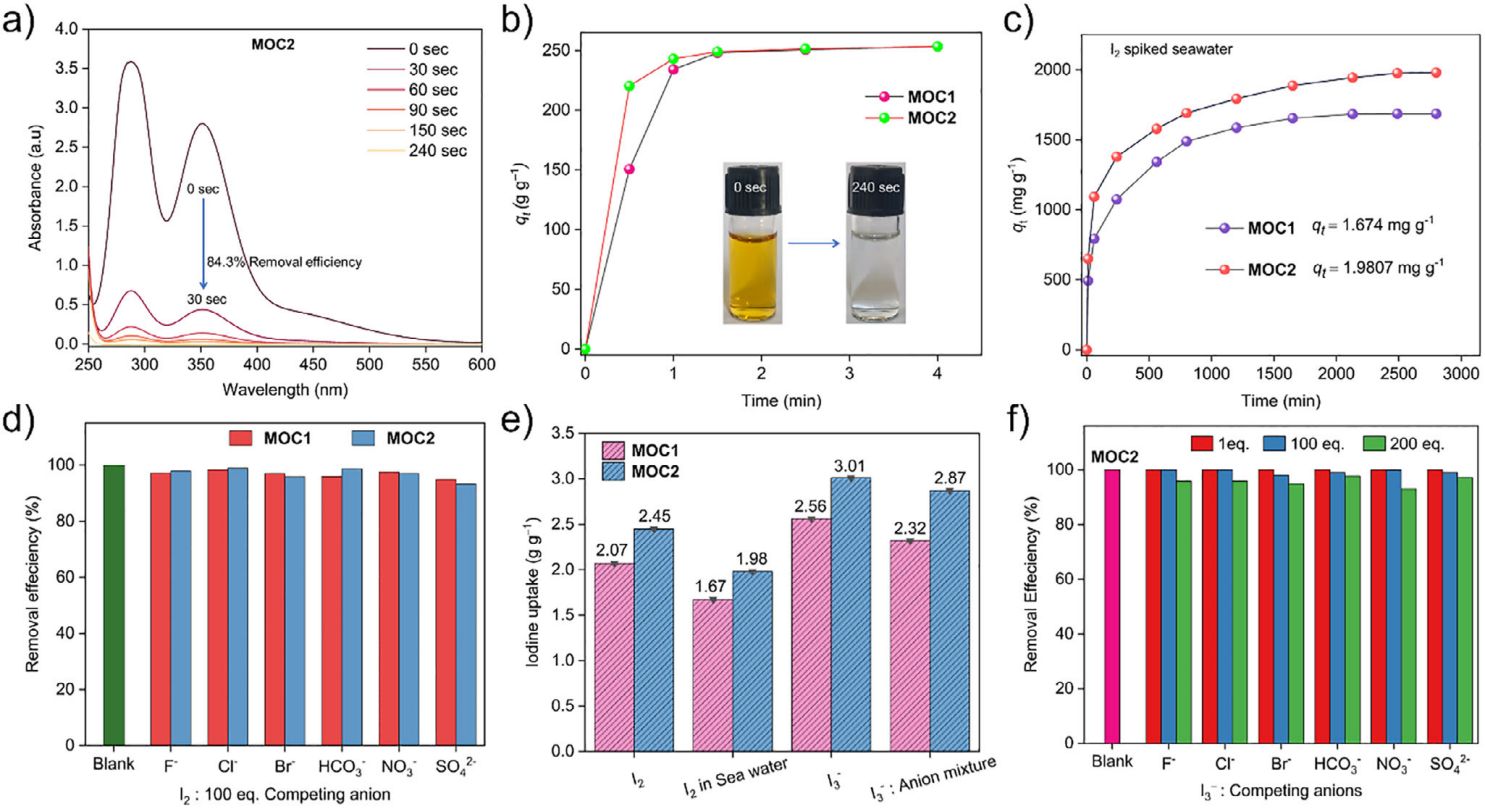
(a) Time‐resolved UV–vis spectra monitoring the progressive removal of I_3_^−^ following the addition of **MOC2**. (b) Non‐linear pseudo‐second‐order kinetic fit describing I_3_^−^ adsorption by MOCs. (c) Time dependent adsorption profile for I_2_ capture from seawater by the MOCs. (d) Selective static adsorption of I_2_ by the MOCs in the presence of 100 equiv. of competing anions. (e) Comparative adsorption performance of both MOCs toward I_2_ and I_3_^−^ in various aqueous environments. (f) Effect of individual competing anions (1−200 equiv.) on I_3_^−^ uptake by **MOC2**.

Beyond molecular iodine, triiodide (I_3_^−^) constitutes one of the major iodine species encountered in aqueous nuclear waste streams and contaminated groundwater. Upon addition of 2 mg of each cage to 3 mL of an aqueous 0.5 mm I_3_^−^ solution, rapid iodine removal was observed.

Notably, **MOC2** achieved >84% I_3_^−^ removal within only ~30 s, whereas **MOC1** required ~60 s to attain comparable efficiency (Figure 4a, Figures S74‒S76). Kinetic analyses revealed that the adsorption process follows a pseudo‒second‒order model with high correlation coefficients, indicating a predominantly chemisorption‒controlled mechanism (Figure 4b, Figure S77). Importantly, the adsorption rate exhibited by **MOC2** ranks among the fastest reported for aqueous iodine capture systems (Table S12). Maximum uptake experiments performed under excess I_3_^−^ conditions (300 mg I_2_ and 600 mg KI in 20 mL water) revealed exceptionally high adsorption capacities of 2.56 and 2.32 g g^−1^ for **MOC1**, and 3.01 and 2.87 g g^−1^ for **MOC2** in the absence and presence of one equivalent of competing anions, respectively (Figure 4e). These uptake values are competitive with state‐of‐the‐art COF, MOF, and POP based iodine sorbents (Table S11). We have also performed I_3_^−^ adsorption experiments over a range of pH values (pH 4–9) using the cage materials. The adsorption profiles showed no significant variation in I_3_^−^ uptake or adsorption behavior across this pH range, indicating that the iodine sequestration capability of the cages is relatively insensitive to moderate changes in the solution pH (Figures S82‒S84). Adsorption experiments at higher pH were not feasible because I_3_^−^ undergoes conversion to IO_3_^−^ under strongly alkaline aqueous conditions. Remarkably, even in the presence of 200 equivalents of interfering anions (F^−^, Cl^−^, Br^−^, HCO_3_^−^, NO_3_^−^, and SO_4_^2−^), both cages retained outstanding I_3_^−^ removal efficiencies of ~95% for **MOC1** and ~96.5% for **MOC2** (Figure 4f, Figure S80). The corresponding distribution coefficients remained on the order of 10^4^ mL g^−1^, comparable to values obtained in the absence of competing ions, highlighting the exceptional selectivity and binding affinity of the cages toward iodine species in highly competitive aqueous environments (Figure S81) [70]. Additionally, we freeze‐dried the as‐prepared metallogels to preserve their three‐dimensional gel morphology and subsequently evaluated their iodine adsorption performance in aqueous media under conditions identical to those used for the as‐synthesized cage powders (Figures S85–S88). Interestingly, the freeze‐dried metallogels exhibited essentially the same iodine adsorption behavior and adsorption performance as the as‐synthesized cage powders, with no significant difference in the adsorption kinetics or overall uptake (Figures S86 and S87, S89 and S90; Table S10). These results suggest that, although the hierarchical gel morphology provides the material with macroscopic self‐healing and soft‐matter properties, the intrinsic iodine‐binding capability is primarily governed by the molecularly defined cage cavities and their associated host−guest interactions. Overall, these findings establish carbazole‐based metal‒organic cages as highly efficient, selective, and robust adsorbents for the removal of both I_2_ and I_3_^−^ from complex aqueous systems, including seawater, highlighting their strong potential for radioactive iodine remediation in environmental and nuclear applications.

Although **MOC1** and **MOC2** exhibited excellent iodine uptake under static conditions, practical water‐treatment applications require efficient adsorption under continuous flow conditions. To evaluate their applicability in realistic remediation processes, dynamic flow‒through column experiments were performed, demonstrating that both cages function as highly effective heterogeneous adsorbents for iodine sequestration from aqueous media (Figure 5d). For molecular iodine adsorption, a 1.2 mm aqueous I_2_ solution was continuously passed through columns packed with the respective MOCs, leading to immediate decolorization of the effluent solution. Under these dynamic conditions, iodine removal efficiencies of ~98.0% for **MOC1** and ~98.87% for **MOC2** were achieved (Figure S94). Analysis of the breakthrough curves revealed dynamic adsorption capacities of 1.46 g g^−1^ for **MOC1** and 1.76 g g^−1^ for **MOC2**, corresponding to breakthrough volumes of 4.8 and 5.8 L g^−1^, respectively (Figure 5a). The dynamic adsorption performance was further investigated using aqueous triiodide (I_3_^−^) solutions. In the absence of competing anions, **MOC1** and **MOC2** afforded high removal efficiencies of ~97% and ~99%, respectively (Figure S95). Importantly, even in the presence of one equivalent of mixed competing anions, both materials retained excellent adsorption performance, maintaining iodine removal efficiencies of ~96% for **MOC1** and ~97.8% for **MOC2** (Figure 5d). Breakthrough analysis showed that the adsorption columns could process up to 6.0 L g^−1^ and 8.2 L g^−1^ of I_3_^−^ solution for **MOC1** and **MOC2**, respectively, before saturation, corresponding to dynamic uptake capacities of 1.14 g g^−1^ for **MOC1** and 1.50 g g^−1^ for **MOC2** (Figure 5b, Figure S96). The reusability of the adsorbents was also assessed through repeated adsorption−desorption cycles using methanol as the regeneration solvent. Notably, **MOC2** retained high iodine removal efficiency and adsorption capacity over at least three consecutive cycles, demonstrating excellent operational stability and recyclability under continuous flow conditions (Figure 5b). These findings highlight the strong potential of the MOC based systems, particularly **MOC2**, for practical iodine remediation in dynamic aqueous environments.

**FIGURE 5:**
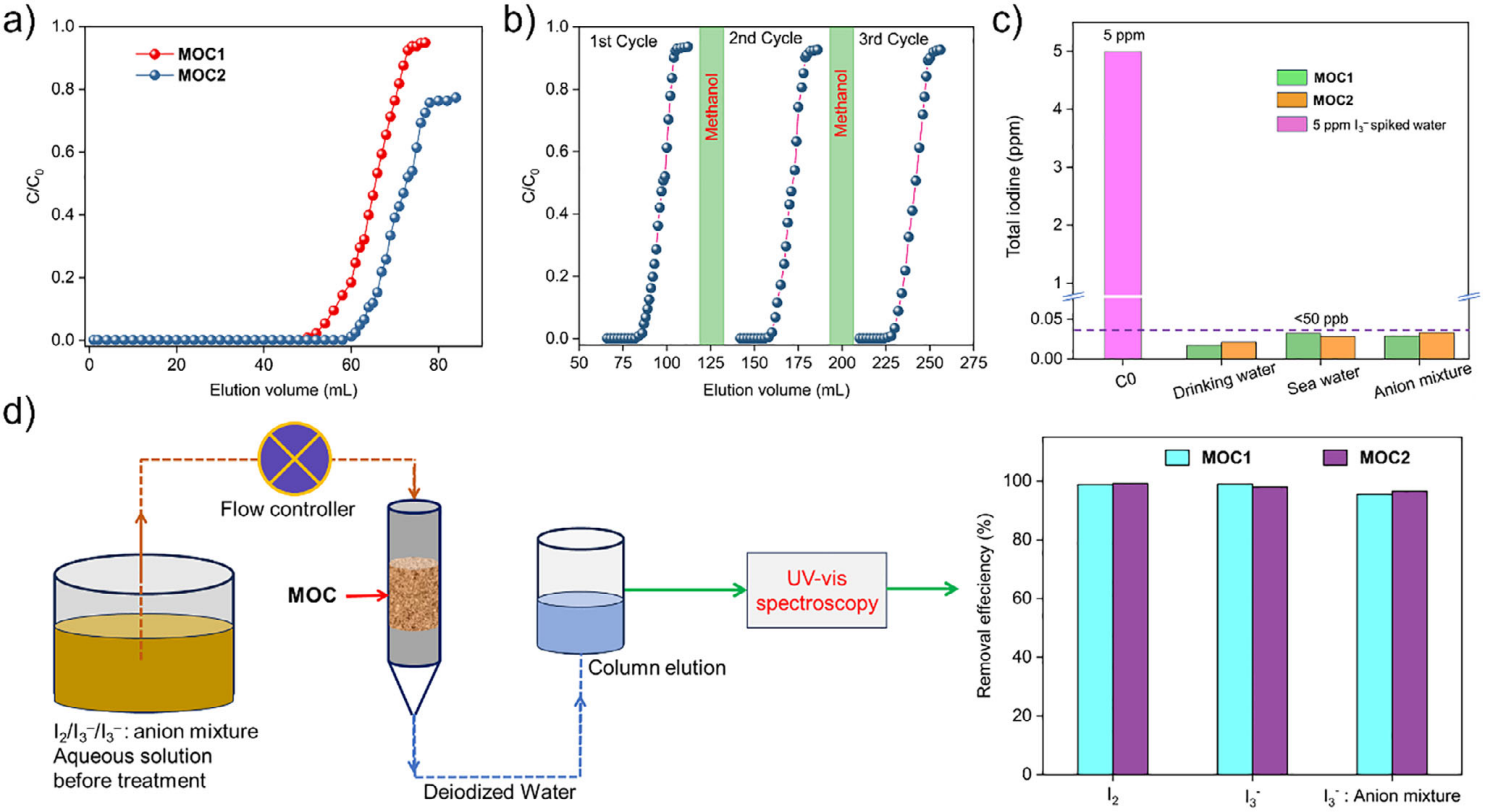
(a) Dynamic breakthrough curves for I_2_ adsorption by the MOCs under continuous flow conditions. (b) Recyclable breakthrough performance of **MOC2** for aqueous I_3_^−^ removal over three successive adsorption–desorption cycles. (c) Adsorption efficiency of the MOCs for ultratrace I_3_^‒^ removal from different aqueous media. (d) Schematic illustration of the continuous‐flow column adsorption process for various iodine species using the MOCs.

To evaluate the practical applicability of the cages under environmentally relevant low‒concentration conditions, dynamic adsorption studies were performed using 5 ppm I_3_^−^ feed solutions prepared in distilled water, seawater, and distilled water containing one equivalent of competing anions. The residual iodine concentration in the treated effluent was determined using the leuco crystal violet colorimetric assay (Scheme S7) [49, 71]. Under these conditions, **MOC1** and **MOC2** effectively reduced iodine concentrations to 24 and 21 ppb, respectively, in distilled water, while residual concentrations of 32 and 47 ppb were obtained in seawater. In the presence of competing anions, the final iodine concentrations remained exceptionally low at 37 ppb for **MOC1** and 35 ppb for **MOC2** (Figure 5c). These residual iodine levels are close to naturally occurring background concentrations in environmental waters and are significantly below levels considered hazardous to human health [70]. Despite possessing similar cage dimensions and identical cationic charge, **MOC2** consistently exhibited superior adsorption performance relative to **MOC1**. This enhancement is attributed to the phenyl substituent present in ligand **L2**, which increases the overall π‒electron density of the cage framework and strengthens π‒iodine interactions, thereby facilitating more effective iodine capture under continuous−flow conditions. Collectively, these findings demonstrate that the MOCs, particularly **MOC2**, function as highly efficient, selective, and recyclable heterogeneous adsorbents for the removal of trace iodine species from complex aqueous environments, highlighting their considerable promise for nuclear wastewater treatment and large‐scale environmental remediation applications.

The adsorption studies demonstrate that both **MOC1** and **MOC2** exhibits excellent affinity toward molecular iodine (I_2_) and triiodide (I_3_^‒^) species in aqueous media. To gain mechanistic insight into the iodine capture process and elucidate the nature of the cage−iodine interactions, the iodine‐loaded materials were systematically analyzed using TGA, SEM−EDS, FT−IR, Raman spectroscopy, XPS, and Density functional theory (DFT) calculations. Thermogravimetric analysis of the iodine‐encapsulated cages revealed pronounced weight losses in the temperature range of 40 ^ο^C−180 ^ο^C under nitrogen atmosphere, corresponding to the thermal release of adsorbed iodine species (Figure S98) [72]. The magnitude of the mass loss closely matched the experimentally determined adsorption capacities, confirming substantial iodine incorporation within the cage motifs. Further evidence for strong iodine−cage interactions was obtained from FT−IR spectroscopy. Upon iodine adsorption, distinct shifts in several characteristic vibrational bands were observed for both cages. In particular, the aromatic C═C stretching vibrations shifted from 1516.04 and 1518.23 cm^−1^ to 1513.19 and 1516.12 cm^−1^, while the C─H stretching bands moved from 1339.89 and 1325.16 cm^−1^ to 1333.08 and 1323.66 cm^−1^ for **MOC1** and **MOC2**, respectively (Figure S97).

These systematic spectral changes indicate direct involvement of the π‒rich carbazole framework in iodine binding through charge‒transfer and π−iodine interactions. Morphological changes associated with iodine uptake were investigated by SEM analysis. Pristine **MOC1** and **MOC2** exhibited relatively uniform morphologies with broad particle‒size distributions, whereas the iodine‐loaded materials adopted noticeably expanded and fluffy textures, consistent with significant iodine incorporation into the supramolecular assemblies (Figure S100). Correspondingly, EDS spectra of the iodine‐loaded cages displayed intense iodine signals in the 3.5−5.0 keV region, with signal intensities proportional to the iodine loading levels (Figures S101 and S102) [73]. Elemental mapping further confirmed a dense and homogeneous distribution of iodine throughout the cage materials, supporting uniform iodine−cage interactions. Raman spectroscopy provided direct insight into the chemical speciation of the adsorbed iodine species. The iodine‐loaded cages exhibited characteristic Raman bands at ∼106.60 and 107.83 cm^−1^ corresponding to symmetric stretching vibrations of I_3_^−^, while bands at ∼135.89 and 138.53 cm^−1^ were assigned to asymmetric I_3_^−^ stretching modes for **MOC1** and **MOC2**, respectively (Figure 6c). Additional signals at ∼160.72 and 162.19 cm^−1^ were attributed to the stretching of I_5_^−^ species, indicating that the adsorbed iodine undergoes partial conversion into polyiodides within the cage environment. A weak broad feature centered around 215−220 cm^−1^ further suggested the coexistence of molecular iodine within the **MOC1** framework [74, 75]. These observations collectively indicate that the electron‐rich carbazole ligands and cationic cage cavities facilitate stabilization of polyiodide species through charge‐transfer interactions. X‐ray photoelectron spectroscopy (XPS) further clarified the electronic interactions between iodine and the cage framework. The I 3d spectra of iodine‐loaded cages exhibit characteristic doublets at 631.83 and 620.44 eV for I_2_@**MOC1** and 631.81 and 620.34 eV for I_2_@**MOC2**, corresponding to I 3d_5/2_, along with peaks at 630.23 and 618.81 eV for I_2_@**MOC1** and 630.14 and 618.93 eV for I_2_@**MOC2** assigned to I 3d_3/2_ (Figure 6a, Figure S104). Quantitative deconvolution of the spectra revealed polyiodide compositions of ~64.8% I_3_^−^ and ~35.2% I_5_^−^ for I_2_@**MOC1**, and ~63% I_3_^−^ and ~37% I_5_^−^ for I_2_@**MOC2** [76]. Moreover, the aromatic C═C‐associated C 1s signals shifted from 284.92 and 285.37 to 285.42 and 285.56 eV (Figure 6b, Figure S103) [77]. Collectively, these spectroscopic and analytical studies establish that iodine adsorption by **MOC1** and **MOC2** is dominated by chemisorption involving synergistic π‒donor interactions from the carbazole units together with strong electrostatic attraction within the cationic cage. This cooperative binding environment promotes efficient charge transfer and stabilization of polyiodide species such as I_3_^−^ and I_5_^−^, thereby accounting for the exceptional iodine uptake performance of the metal‒organic cages.

**FIGURE 6:**
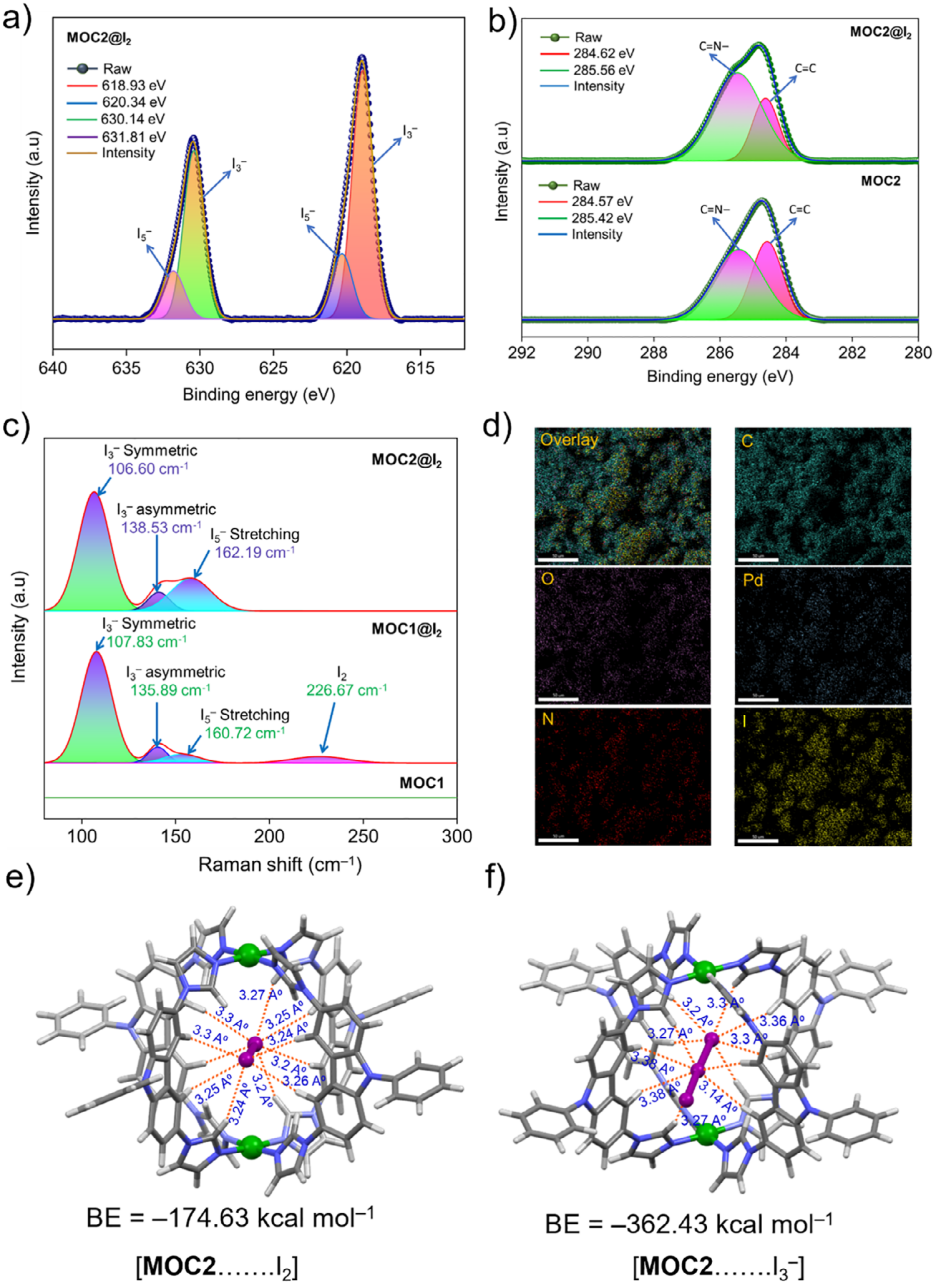
(a) High‐resolution XPS spectrum of the I 3d region for iodine‐loaded I_2_@**MOC2**. (b) Comparative C 1s XPS spectra of pristine and iodine‐adsorbed **MOC2**. (c) Raman spectra of the MOCs before and after iodine adsorption. (d) EDS elemental mapping of iodine distribution in I_2_@**MOC2**. DFT‒optimized structures illustrating different interaction sites and the corresponding binding energies for (e) **MOC2**@I_2_ and (f) **MOC2**@I_3_^‒^.

Methanol proved to be an efficient regeneration solvent, as demonstrated by the gradual release of iodine monitored using UV–vis spectroscopy (Figures S92‒S67). Time dependent increases in the absorbance at 360 and 445 nm confirmed the controlled recovery of adsorbed iodine species, validating the recyclability of the adsorbent system. Importantly, ^1^H NMR spectra of the regenerated cages remained identical to those of the pristine materials, confirming that the cage structures remain intact after repeated adsorption−desorption cycles and highlighting their excellent structural robustness for long term iodine remediation applications (Figures S105 and S106).

We made multiple attempts to obtain suitable single crystals of the iodine‐loaded MOC complexes under various crystallization conditions, including different solvents, solvent combinations, iodine concentrations, and crystallization protocols. However, despite these extensive efforts, we were unable to obtain single crystals of sufficient quality for single‐crystal X‐ray diffraction analysis. Therefore, to obtain deeper molecular‐level insight into the iodine adsorption process, DFT calculations were carried out for the cages **MOC1** and **MOC2** interacting with I_2_ and I_3_^−^ species [78]. Electrostatic potential (ESP) surface analysis revealed that the cationic nature of the cages, arising from the Pd centers together with positively polarized regions between neighboring aromatic ligands, generates a highly favorable electrostatic environment for iodine binding (Figure S107) [79]. These positively charged regions strongly promote strong interactions with iodine species, particularly with the anionic I_3_^−^ species. Consistent with this interpretation, the calculated binding energies indicate substantially stronger interactions for I_3_^−^ than for neutral I_2_, highlighting the dominant contribution of electrostatic attraction between the polyiodide anions and the cationic cage structure. The theoretical calculations further demonstrate that **MOC2** exhibits significantly higher binding affinity toward both iodine species relative to **MOC1**. For **MOC2**, the calculated binding energies were −174.63, −158.67, and −153.06 kcal mol^−1^ for different I_2_ binding configurations and −362.43, −310.53, and −310.13 kcal mol^−1^ for I_3_^−^ binding configurations, whereas comparatively weaker interactions were obtained for **MOC1**, with binding energies ranging from −154.54 to −131.80 kcal mol^−1^ for I_2_ and from −345.76 to −292.82 kcal mol^−1^ for I_3_^−^ (Figure 6e,f, Figures S108‒S111). These results are fully consistent with the experimentally observed superior iodine uptake performance of **MOC2**. For both cage systems, iodine encapsulation within the internal cavity was calculated to be energetically more favorable than adsorption at external surface sites. In particular, cavity‒confined iodine species exhibited significantly stronger binding energies than externally associated species, emphasizing the important role of confinement effects and host−guest stabilization within the cage cavities (Figure 6e,f, Figures S108–S111) [80]. Together, the DFT results support the experimental findings and confirm that the exceptional iodine capture performance arises from the synergistic interplay of electrostatic interactions, confinement effects, and charge‐transfer interactions within the π‒electron‐rich cage framework.

The iodine adsorption studies further establish that the functional performance of these cages originates from their molecularly engineered internal and external environments. In particular, the π‐electron‐rich carbazole framework and cationic Pd_2_L_4_ architecture provide a cooperative platform in which electrostatic attraction, confinement, and π−iodine/charge‐transfer interactions collectively promote guest stabilization. Notably, these same structural and electronic features are also expected to influence the excited‐state behavior of the cages. The extended π‐conjugated carbazole chromophores can act as light‐responsive units, while the heavy Pd(II) centers may facilitate intersystem crossing and the generation of reactive excited states capable of activating molecular oxygen. Thus, the electronic characteristics that enable efficient iodine recognition also provide a molecular basis for a second function−photosensitized catalytic reactivity. The photocatalytic activity therefore emerges from the same ligand‐engineered cage platform, with the carbazole chromophores and Pd(II) centers collectively controlling both host‐guest interactions and excited‐state processes. On this basis, we next investigated the photophysical properties of **MOC1** and **MOC2** and explored their potential as heterogeneous photocatalysts for visible‐light‐driven aerobic sulfide oxidation.

### 2.4 Photocatalytic Activity

The preceding studies establish that the multifunctionality of the present system originates from a common molecular design rather than from three independent properties. The Pd_2_L_4_ architecture provides a well‐defined and cationic molecular framework, while the carbazole ligands simultaneously contribute π‐rich surfaces for host−guest recognition and extended conjugation for light harvesting. The resulting combination of structural confinement, electronic polarization, and Pd(II)‐mediated excited‐state processes provides a molecular basis for both iodine sequestration and photocatalytic activity [53]. In this context, the iodine‐binding studies demonstrate the ability of the cages to engage strongly with electron‐deficient iodine species through electrostatic, confinement, and π−iodine/charge‐transfer interactions, whereas the same π‐conjugated framework is expected to support long‐lived excited states and reactive oxygen species generation [59, 60]. We therefore investigated whether rational modification of the carbazole substituent could simultaneously tune the cage assembly, electronic structure, and photocatalytic response. In particular, comparison of **MOC1** and **MOC2** provides an opportunity to correlate ligand substitution with the hierarchical material properties, iodine‐binding behavior, and photochemical performance of the resulting cage systems.

The photophysical properties of **MOC1** and **MOC2** were examined by UV−Vis absorption spectroscopy in DMSO. Both cages display strong absorption bands within the UV region (254−380 nm), indicating their suitability for activation under blue‐light irradiation (Figure 7a). Optical band gaps estimated from Tauc plot analysis were determined to be 3.34 eV for **MOC1** and 3.29 eV for **MOC2** (Figure 7a, inset). The slightly narrower band gap observed for **MOC2** originates from the enhanced π‐conjugation imparted by the phenyl substituent in ligand **L2**, which promotes greater electronic delocalization relative to the methyl‐substituted analogue **L1**. To gain further insight into their electronic structures, Mott−Schottky analyses were conducted using an Ag/AgCl reference electrode at multiple frequencies. From the linear extrapolation of the plots, conduction band energies of −0.94 eV for **MOC1** and −0.81 eV for **MOC2** were obtained (Figure 7b). Using the relation *E_g_* = *E_CB_*  − *E_VB_*, the corresponding valence band positions were calculated to be 2.40 and 2.48 eV for **MOC1** and **MOC2**, respectively, confirming that both systems possess energetically favorable band structures for photocatalytic redox transformations (Figure 7c). Photoluminescence measurements revealed strong blue emission in the range of ∼360–420 nm for both cages, indicative of efficient radiative recombination of photoexcited charge carriers (Figure S114).

**FIGURE 7:**
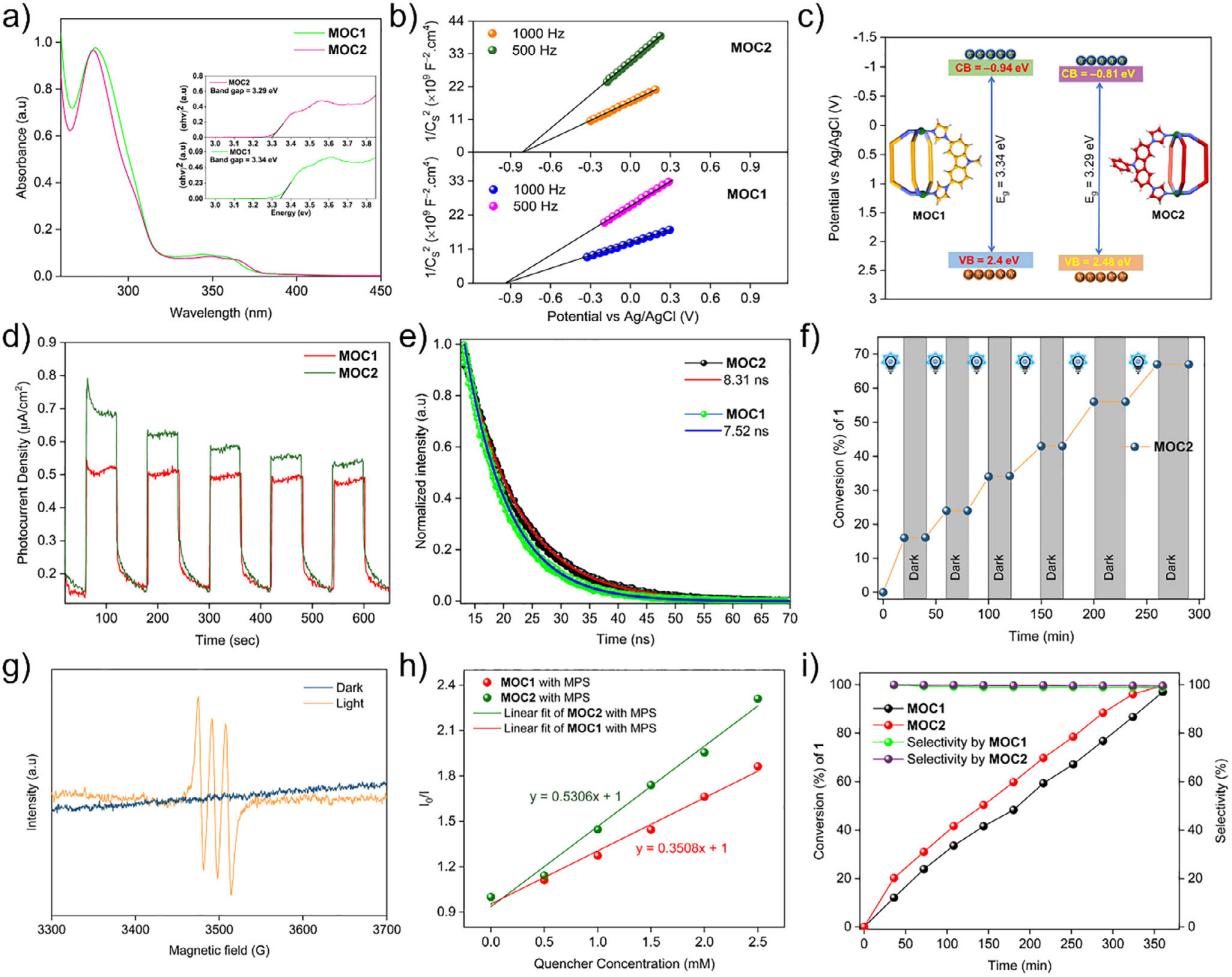
Photophysical, electrochemical, and mechanistic investigations of the MOCs. (a) UV−vis absorption spectra of the MOCs (inset: corresponding Tauc plots). (b) Mott–Schottky plots illustrating the flat‐band potentials. (c) Band energy diagrams illustrating the valence band (VB) and conduction band (CB) positions of **MOC1** and **MOC2**. (d) Transient photocurrent responses recorded under intermittent light irradiation. (e) Excited‐state lifetimes obtained from time‐correlated single‐photon counting (TCSPC) measurements for **MOC1** and **MOC2**. (f) Light on‐off control experiments highlighting the photo‐dependency of the catalytic reaction. (g) EPR spectra confirming the generation of singlet oxygen through oxidation of TEMP to TEMPO in the presence of the **MOC2** under light irradiation. (h) Stern−Volmer quenching analysis of pristine MOCs with thioanisole. (i) Kinetic profiles for the selective aerobic oxidation of thioanisole under optimized conditions.

Relative to **MOC1**, **MOC2** exhibited a slight red shift in emission, reflecting differences in the electronic environment induced by the phenyl substituent. Time‐correlated single‐photon counting (TCSPC) experiments further demonstrated that **MOC2** possesses a longer excited‐state lifetime (8.31 ns) than **MOC1** (7.52 ns) (Figure 7e), suggesting more effective suppression of electron−hole recombination and improved photosensitization efficiency. To directly probe charge‐transfer dynamics, photoelectrochemical studies were performed.

Electrochemical impedance spectroscopy (EIS) measurements showed that **MOC2** possesses a substantially smaller semicircle in the Nyquist plot, corresponding to a lower charge‐transfer resistance (Rct = 1.66 kΩ) compared with **MOC1** (Rct = 3.72 kΩ), indicating more efficient interfacial electron transport (Figure S112). Consistent with this result, linear sweep voltammetry (LSV) under illumination revealed higher photocurrent densities for **MOC2** (2.23 mA cm^−2^) than for **MOC1** (1.86 mA cm^−2^) over the potential range of −1.6 to −0.1 V vs. Ag/AgCl (Figure S113). Transient photocurrent response experiments further supported these observations, with **MOC2** exhibiting a markedly enhanced photocurrent response of 0.8 µA cm^−2^ at 0 V vs. Ag/AgCl relative to **MOC1** (Figure 7d). Collectively, these results demonstrate that phenyl substitution in **MOC2** significantly improves π‐conjugation, narrows the optical band gap, prolongs excited‐state lifetimes, suppresses charge recombination, and enhances charge‐transfer efficiency, thereby leading to superior photocatalytic performance compared with **MOC1** [81].

The combined photophysical and photoelectrochemical studies establish **MOC1** and **MOC2** as promising visible‐light‐responsive heterogeneous photocatalysts. Motivated by these results, we next explored their catalytic performance in the aerobic oxidation of sulfides to sulfoxides using molecular oxygen as the terminal oxidant. Thioanisole (methyl phenyl sulfide, MPS) was selected as a representative model substrate to optimize the photocatalytic reaction conditions. A detailed optimization study involving catalyst loading, irradiation wavelength, light source, solvent, and reaction time was conducted to identify the most efficient catalytic conditions (Figure S117). Among the two cages, **MOC2** consistently exhibited superior photocatalytic activity, affording methyl phenyl sulfoxide (MPSO, **1**) in >99% yield under blue LED irradiation (λ_max_ = 390 nm) using methanol as the reaction medium (Figure 7i). The catalytic efficiency was found to strongly depend on the irradiation wavelength, with maximum activity observed at 390 nm, while alternative solvents led to significantly lower product yields. Under the optimized conditions (0.5 mol% **MOC2**, methanol, O_2_ purging for 2 min, blue LED irradiation at 390 nm, room temperature, 6 h), the oxidation proceeded with near‐quantitative conversion and exceptional selectivity (~99%) toward the corresponding sulfoxide (Figure S117). Comparative studies demonstrated that **MOC2** outperformed **MOC1** in both catalytic efficiency and product selectivity (Figure 7i).

Control experiments confirmed the critical roles of light, oxygen, and catalyst in the reaction process the yield decreased substantially (∼64%) in the absence of oxygen, no product formation was observed in the dark, and catalyst‐free reactions produced only ∼19% yield. The conversion of MPS and formation of MPSO were monitored by ^1^H NMR spectroscopy (Figure 7i, Figure S121). Time‐dependent spectral analysis showed a gradual decrease in the characteristic resonances of MPS at δ 7.10, 7.24, and 2.43 ppm accompanied by the appearance of new signals corresponding to MPSO at δ 7.68, 7.58, and 2.67 ppm (Figure S121). Importantly, **MOC2** enabled complete conversion of MPS to MPSO within 6 h in methanol without detectable formation of the overoxidized sulfone byproduct (<1%), highlighting the remarkable chemoselectivity of the catalytic system toward partial oxidation (Figure 7i, Figure S121). Kinetic investigations revealed that the oxidation reaction follows apparent zero‐order kinetics, with **MOC2** exhibiting a rate constant of 4.505 ×10^‒4^ mol L^‒1^ s^‒1^ and a corresponding half‐life (t_1/2_) of 178.6 min (Figure S122) [82]. Furthermore, the turnover frequency (TOF) of **MOC2** (29.42 h^−1^) exceeds that of **MOC1** (27.93 h^−1^) and surpasses the values reported for most previously described photocatalysts (Tables S1–S15) [82, 83]. To demonstrate the versatility and substrate tolerance of the photocatalytic system, a diverse range of thioanisole derivatives containing both electron‐donating and electron‐withdrawing substituents was examined under the optimized reaction conditions (Table 1). In all cases, **MOC2** efficiently catalyzed the aerobic oxidation of the sulfides to their corresponding sulfoxides, generally affording yields ≥70% together with excellent selectivity of ~97%. Importantly, overoxidation to the undesired sulfone byproducts remained below 3% for all substrates investigated, highlighting the exceptional chemoselectivity of the catalytic system. Although **MOC1** also exhibited high selectivity, its catalytic activity was consistently lower than that of **MOC2**, emphasizing the beneficial role of the extended π−conjugation and improved charge‐transfer properties present in **MOC2**. As anticipated, substrates bearing electron‐donating substituents displayed enhanced reactivity and higher turnover frequencies (TOFs), with *p*‐Me and *p*‐OMe derivatives of thioanisoles exhibiting TOF values of 27.81 and 29.13 h^‒1^, respectively (Table 1, Figures S125–S128). In contrast, substrates containing electron‐withdrawing groups showed relatively slower reaction rates because of reduced electron density at the sulfur center. Nevertheless, even strongly deactivating substituents such as aldehyde and nitro groups underwent efficient oxidation, providing high product yields (typically ≥70% for **MOC2**) and excellent selectivity, with TOFs of 20.15 h^‒1^ for *p*‐CHO and 23.19 h^‒1^ for *p*‐NO_2_ derivatives (Table 1). Halogen‐substituted aromatic thioethers were also well tolerated, particularly para‐halogenated substrates, for which **MOC2** consistently achieved product yields exceeding 80% while maintaining selectivity above 97% (Table 1, Figures S129 and S130). Furthermore, replacing the methyl substituent in thioanisole with an ethyl group resulted in almost quantitative conversion with **MOC2** (99%) and high conversion with **MOC1** (86%), while preserving outstanding selectivity (99%) in both cases (Table 1, Figures S123 and S124). The broad substrate scope, high catalytic activity, and exceptional selectivity demonstrated by **MOC2** highlight its effectiveness as a heterogeneous photocatalyst for the selective aerobic oxidation of sulfur‐containing compounds. These findings further suggest its potential applicability in environmental detoxification and remediation processes involving sulfur‐containing toxic agents. The recyclability and operational durability of the catalysts were also investigated to assess their practical applicability. The photocatalysts could be readily recovered and reused over five consecutive catalytic cycles with only a slight decrease in activity. Even after the fifth run, **MOC1** and **MOC2** retained high catalytic efficiencies, providing product yields of 84% and 87%, respectively (Figure 8a). We have evaluated the photocatalytic efficiency of the freeze‐dried MOCGs under the same reaction conditions used for the corresponding as‐synthesized MOC powders. Interestingly, the MOCGs exhibited no significant difference in catalytic activity compared with the corresponding MOC powders, indicating that the hierarchical gel morphology does not adversely affect the intrinsic photocatalytic performance of the cage units (Figures S131‒133).

**TABLE 1:** Substrate scope for the heterogeneous photocatalytic oxidation of various thioethers to sulfoxides using the MOC catalysts with high conversion and selectivity^a^.

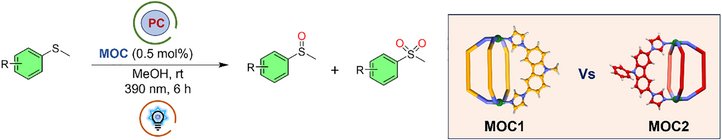						
Entry	Reactant	Product	Con.(%)^b^	Con.(%)^c^	Sel.(%)^d^	TOF (h^−1^)^e^
1	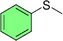	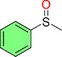	96	>99	>99	29.42
2	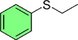	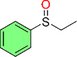	86	99	>99	29.57
3	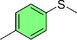	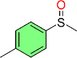	83	93	98	27.81
4	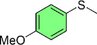	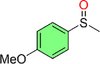	85	98	97	29.13
5	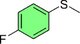	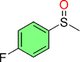	74	86	98	25.78
6	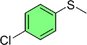	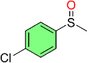	72	81	97	25.59
7	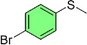	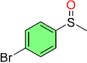	69	80	99	24.71
8	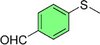	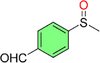	58	70	99	20.15
9	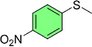	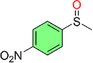	71	76	98	23.19

^a^ Reaction condition: Catalyst **MOC** (0.5 mol%); 1mL of MeOH; 390 nm Light irradiation for 6h; 0_2_ purge for 2 min; Room Temperature.

^b^ Conversion By **MOC1**.

^c^ Conversion by **MOC2**.

^d^ Selectivity.

^e^ TOF using the catalyst **MOC2**. Conversion and selectivity was measured by the ^1^H NMR spectroscopy.

**FIGURE 8:**
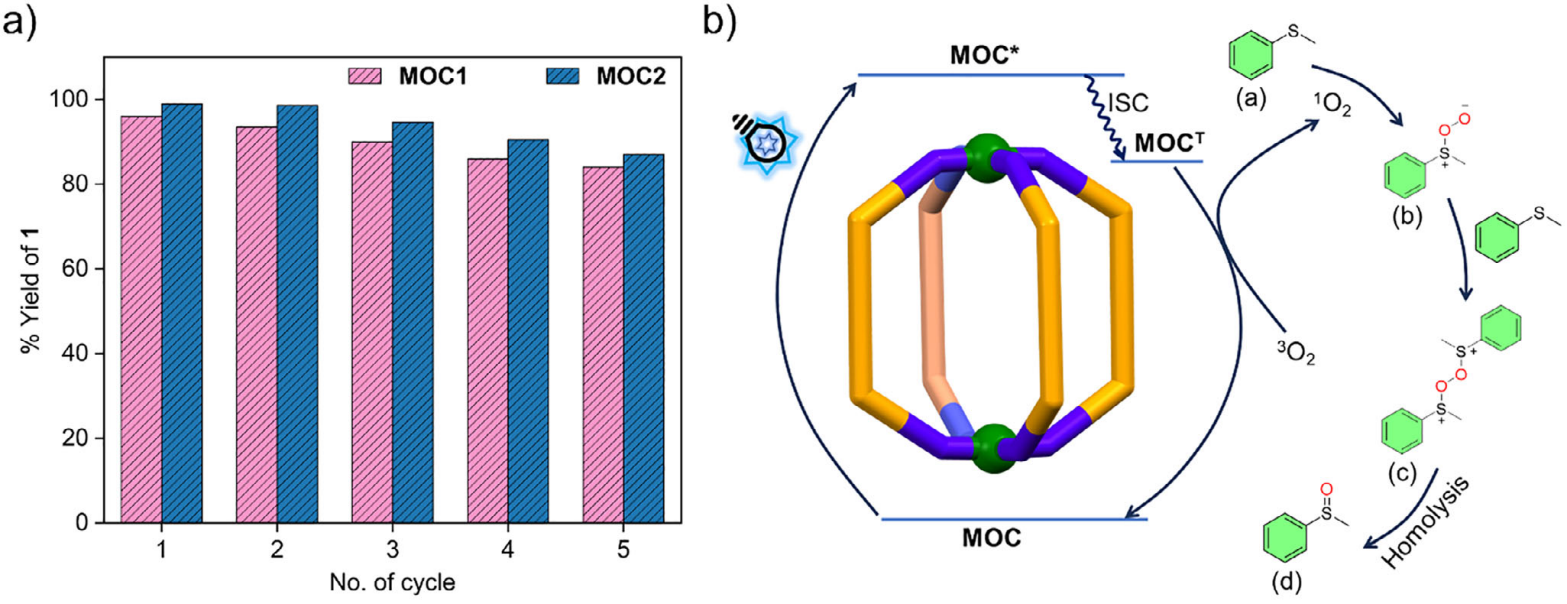
(a) Reusability of the MOCs in the photocatalytic reaction. (b) Proposed mechanistic pathway for the MOC catalyzed photocatalytic oxidation of sulfides to sulfoxides under visible‐light irradiation.

We have performed a comprehensive set of experiments to evaluate the solubility, heterogeneity, and possible leaching of **MOC1** and **MOC2** under the catalytic conditions. First, the solubility of both cages was assessed in MeOH, H_2_O, acetonitrile, CHCl_3_, DCM, and DMSO using ^1^H NMR spectroscopy (Figures S134 and S135). Well‐resolved cage resonances were observed only in DMSO‐*d*_6_, confirming appreciable solubility in DMSO, whereas no characteristic cage signals were detected in the other solvents, indicating that **MOC1** and **MOC2** are essentially insoluble in these media. These results are consistent with the heterogeneous nature of the photocatalytic reaction performed in methanol. We further conducted a hot‐filtration experiment to directly evaluate whether catalysis arises from dissolved molecular species (Figures S136 and S137). During the reaction, the reaction mixture was filtered at the reaction temperature to remove the suspended solid catalyst, and the resulting filtrate was subsequently maintained under identical reaction conditions in the absence of the solid catalyst. No significant increase in product formation was observed after hot filtration, indicating that the catalytically active species is predominantly associated with the heterogeneous cage material rather than a dissolved molecular species.

To further assess possible catalyst leaching, the filtered reaction mixtures were analyzed by ^1^H NMR spectroscopy and ICP‐OES. No characteristic cage resonances were observed in the ^1^H NMR spectra of the filtrates in DMSO‐*d*_6_, indicating that significant dissolution of the cage complexes did not occur (Figures S138 and S139). ICP‐OES analysis further showed that only ~3% of the cage material leached into the reaction solution for both **MOC1** and **MOC2** (Table S13). We also examined the structural integrity of the catalysts before and after the catalytic reaction using ^1^H NMR and XPS spectroscopy. No appreciable changes were observed in either the ^1^H NMR or XPS profiles, indicating that the cage catalysts remain structurally intact under the reaction conditions (Figures S141‒S144). Mechanistic investigations, together with previous reports, indicate that the photocatalytic aerobic oxidation of thioanisole proceeds predominantly through reactive oxygen species (ROS), particularly singlet oxygen (^1^O_2_), generated by the photoexcited metal−organic cages [84]. Radical trapping experiments using 2,2,6,6‐tetramethylpiperidin‐1‐oxyl (TEMPO) significantly suppressed the reaction efficiency, reducing the conversion to ~15%, thereby confirming the involvement of radical‐mediated intermediates during the catalytic process. Generation of ^1^O_2_ proceeds via a photosensitized pathway, wherein energy is transferred from the triplet excited state of the MOC to ground‐state molecular oxygen (^3^O_2)_. Consistent with this mechanism, electron spin resonance (ESR) spectroscopy performed under light irradiation in the presence of TEMP exhibited the characteristic three‐line signal corresponding to TEMPO formation, confirming the generation of singlet oxygen in methanol in the presence of the MOC catalyst. In contrast, no detectable ESR signal was observed in the absence of light irradiation (Figure 7g). We performed additional control experiments using a series of selective radical and charge‐carrier scavengers under otherwise identical photocatalytic conditions. Specifically, NaN_3_ was employed as a singlet‐oxygen (^1^O_2_) scavenger, isopropanol (IPA) as a hydroxyl‐radical (^•^OH) scavenger, AgNO_3_ as an electron scavenger, KI as a hole scavenger, 1,4‐benzoquinone (BQ) as a superoxide‐radical (O_2_^•−^) scavenger, and 1,1‐diphenylethylene (DPE) as a sulfur‐radical trapping reagent. Interestingly, a pronounced suppression of product formation was observed only in the presence of NaN_3_, whereas the addition of IPA, AgNO_3_, KI, BQ, or DPE did not cause any significant change in the product yield (Figures S118 and S119). These results strongly support ^1^O_2_ as the dominant reactive oxygen species responsible for the photocatalytic oxidation, while providing no significant evidence for the involvement of ^•^OH, O_2_^•−^, photogenerated electrons/holes, or sulfur‐radical intermediates under the investigated conditions. In addition, intermittent light on/off experiments revealed that the reaction immediately ceased when the light source was removed, ruling out the possibility of a self‐sustaining radical chain process operating independently of continuous photoexcitation (Figure 7f). Collectively, these findings demonstrate that the catalytic oxidation process is governed by photoinduced ROS generation through a dominant energy‐transfer pathway involving singlet oxygen.

To further understand the interaction between the excited‐state cages and the sulfide substrate, steady‐state photoluminescence quenching experiments were performed using methyl phenyl sulfide (MPS) as a quencher for both **MOC1** and **MOC2**. Gradual addition of MPS resulted in significant quenching of the emission intensities of both cages (Figure S120). Stern−Volmer analysis using the relation I_0_/I = 1 + *K*_sv_[Q] afforded quenching constants (*K*_sv_) of 350.8 m ^−1^ for **MOC1** and 530.6 M^−1^ for **MOC2**. The larger quenching constant observed for **MOC2** indicates more efficient excited‐state interaction and energy transfer between the photoexcited cage and the sulfide substrate (Figure 7h), consistent with the enhanceds photocatalytic activity exhibited by **MOC2** during sulfide oxidation [81].

Based on these mechanistic studies and related literature precedents, a plausible photocatalytic pathway for sulfide oxidation is proposed (Figure 8b) [82, 83, 85, 86]. Upon visible‐light irradiation, the MOC is initially excited to its singlet excited state (MOC*), followed by rapid intersystem crossing to the long‐lived triplet excited state (MOC^T^). This triplet excited species subsequently transfers energy to ground‐state molecular oxygen (^3^O_2_), generating highly reactive singlet oxygen (^1^O_2_) while regenerating the ground‐state catalyst. The generated ^1^O_2_ then reacts with methyl phenyl sulfide to form a persulfoxide intermediate, which subsequently reacts with another sulfide molecule to furnish the corresponding sulfoxide product, thereby completing the catalytic cycle. The predominance of this singlet oxygen‐mediated energy‐transfer pathway accounts for both the high catalytic efficiency and the remarkable selectivity observed during the photocatalytic oxidation process. To the best of our knowledge, this study represents the first example of a self‐assembled coordination cage functioning as a heterogeneous photocatalyst under visible‐light irradiation. Importantly, the present catalytic system operates efficiently under mild visible‐light irradiation without the need for additional oxidants such as H_2_O_2_ [87], thereby minimizing energy consumption and avoiding the use of stoichiometric oxidizing reagents compared to previously reported systems [88]. Despite these mild reaction conditions, the MOC catalysts exhibit excellent photocatalytic performance, highlighting the effectiveness of rational ligand engineering. In particular, the phenyl‐functionalized **MOC2** demonstrates that modulation of ligand π−conjugation provides an effective strategy for tuning the electronic structure, narrowing the band gap, and enhancing the photocatalytic activity of metal–organic cage systems.

## 3 Conclusion

Collectively, these results reveal a common molecular design principle underlying the multifunctionality of the present cage platform. The Pd(II)‐directed assembly defines a robust and cationic molecular cavity, while the π‐conjugated carbazole ligands simultaneously govern hierarchical intermolecular organization, iodine recognition, and photoinduced electronic processes. Consequently, the same structural features enable the discrete cages to undergo higher‐order assembly into adaptive self‐healing metallogels, selectively stabilize iodine species through cooperative confinement and π−iodine/electrostatic interactions, and generate reactive oxygen species for selective aerobic photocatalysis. The systematic differences between **MOC1** and **MOC2** further demonstrate that ligand‐level modulation of π‐conjugation can tune both supramolecular organization and functional performance. Thus, rather than representing three independent functionalities, the present study establishes a unified cage‐based design strategy in which molecular architecture, hierarchical assembly, host–guest recognition, and excited‐state reactivity are interconnected within a single supramolecular platform. This approach provides a general framework for developing multifunctional, adaptive soft materials that integrate structural responsiveness, environmental remediation, and sustainable catalysis.

[CCDC 2523369 and 2523370 for the cages **MOC1** and **MOC2**, respectively contains the supplementary crystallographic data for this paper. These data can be obtained free of charge from The Cambridge Crystallographic Data Centre via www.ccdc.cam.ac.uk/data_request/cif.]

## Author Contributions

**Monotosh Dalapati**: investigation, validation, methodology, conceptualization, formal analysis, writing – original draft, writing – review and editing. **Raghunath Singha**: methodology, data curation, formal analysis, investigation. **Debashree Manna**: formal analysis, investigation. **Dipak Samanta**: conceptualization, data curation, visualization, methodology, investigation, supervision, project administration, funding acquisition, validation, formal analysis, writing – original draft, writing – review and editing.

## Conflicts of Interest

The authors declare no conflicts of interest.

## Supporting information




**Supporting File**: advs78042‐sup‐0001‐SuppMat.docx.

## Data Availability

The data that supports the findings of this study are available in the Supporting Information of this article.

## References

[1] S. Wang and M. W. Urban , “Self‐healing Polymers,” Nature Reviews Materials 5, no. 8 (2020): 562–583, 10.1038/s41578-020-0202-4.

[2] M. Wang , P. Zhang , M. Shamsi , et al., “Tough and Stretchable Ionogels by in Situ Phase Separation,” Nature Materials 21, no. 3 (2022): 359–365, 10.1038/s41563-022-01195-4. 35190655

[3] J.‐H. Chen , C. Liu , L. You , and C. A. Simmons , “Boning up on Wolff's Law: Mechanical Regulation of the Cells That Make and Maintain Bone,” Journal of Biomechanics 43, no. 1 (2010): 108–118, 10.1016/j.jbiomech.2009.09.016. 19818443

[4] P. Yang , Z. Li , B. Fang , and L. Liu , “Self‐healing Hydrogels Based on Biological Macromolecules in Wound Healing: a Review,” International Journal of Biological Macromolecules 253, no. 12 (2023): 127612, 10.1016/j.ijbiomac.2023.127612. 37871725

[5] J. von Szczepanski , P. M. Danner , and D. M. Opris , “Self‐Healable, Self‐Repairable, and Recyclable Electrically Responsive Artificial Muscles,” Advanced Science 9, no. 22 (2022): 2202153, 10.1002/advs.202202153. PMC9353453 35657031

[6] A. H. Pandit , N. Mazumdar , K. Imtiyaz , M. M. Alam Rizvi , and S. Ahmad , “Self‐Healing and Injectable Hydrogels for Anticancer Drug Delivery: a Study with Multialdehyde Gum Arabic and Succinic Anhydride Chitosan,” ACS Applied Bio Materials 3, no. 12 (2020): 8460–8470, 10.1021/acsabm.0c00835. 35019617

[7] H. Wang and S. C. Heilshorn , “Adaptable Hydrogel Networks with Reversible Linkages for Tissue Engineering,” Advanced Materials 27, no. 25 (2015): 3717–3736, 10.1002/adma.201501558. PMC4528979 25989348

[8] S. Lanzalaco and E. Armelin , “Poly(N‐isopropylacrylamide) and Copolymers: a Review on Recent Progresses in Biomedical Applications,” Gels 3, no. 4 (2017): 36, 10.3390/gels3040036. PMC6318659 30920531

[9] H. Qin , T. Zhang , H.‐N. Li , H.‐P. Cong , M. Antonietti , and S.‐H. Yu , “Dynamic Au‐Thiolate Interaction Induced Rapid Self‐Healing Nanocomposite Hydrogels with Remarkable Mechanical Behaviors,” Chem 3, no. 4 (2017): 691–705, 10.1016/j.chempr.2017.07.017.

[10] R. Gu , H. Zhou , Z. Zhang , et al., “Research Progress Related to Thermosensitive Hydrogel Dressings in Wound Healing: a Review,” Nanoscale Advances 5, no. 22 (2023): 6017–6037, 10.1039/d3na00407d. PMC10629053 37941954

[11] R. Fan , Y. Cheng , R. Wang , et al., “Thermosensitive Hydrogels and Advances in Their Application in Disease Therapy,” Polymers 14, no. 12 (2022): 2379, 10.3390/polym14122379. PMC9227257 35745954

[12] D. Y. Ko , U. P. Shinde , B. Yeon , and B. Jeong , “Recent Progress of in Situ Formed Gels for Biomedical Applications,” Progress in Polymer Science 38, no. 3‒4 (2013): 672–701, 10.1016/j.progpolymsci.2012.08.002.

[13] J. Qu , X. Zhao , Y. Liang , T. Zhang , P. X. Ma , and B. Guo , “Antibacterial Adhesive Injectable Hydrogels with Rapid Self‐healing, Extensibility and Compressibility as Wound Dressing for Joints Skin Wound Healing,” Biomaterials 183 (2018): 185–199, 10.1016/j.biomaterials.2018.08.044. 30172244

[14] H. Yin , F. Liu , T. Abdiryim , and X. Liu , “Self‐Healing Hydrogels: From Synthesis to Multiple Applications,” ACS Materials Letters 5, no. 7 (2023): 1787–1830, 10.1021/acsmaterialslett.3c00320.

[15] J. Zhou , X. Du , Y. Gao , J. Shi , and B. Xu , “Aromatic–Aromatic Interactions Enhance Interfiber Contacts for Enzymatic Formation of a Spontaneously Aligned Supramolecular Hydrogel,” Journal of the American Chemical Society the American Chemical Society 136, no. 8 (2014): 2970–2973, 10.1021/ja4127399. PMC3986011 24512553

[16] G. O. Lloyd and J. W. Steed , “Anion‐tuning of Supramolecular Gel Properties,” Nature Chemistry 1, no. 6 (2009): 437–442, 10.1038/nchem.283. 21378911

[17] S. Panja and D. J. Adams , “Stimuli Responsive Dynamic Transformations in Supramolecular Gels,” Chemical Society Reviews 50, no. 8 (2021): 5165–5200, 10.1039/d0cs01166e. 33646219

[18] P. Dastidar , S. Ganguly , and K. Sarkar , “Metallogels From Coordination Complexes, Organometallic, and Coordination Polymers,” Chemistry – An Asian Journal 11, no. 18 (2016): 2484–2498, 10.1002/asia.201600814. 27410949

[19] J. Zhang and C.‐Y. Su , “Metal‐organic Gels: From Discrete Metallogelators to Coordination Polymers,” Coordination Chemistry Reviews 257, no. 7‒8 (2013): 1373–1408, 10.1016/j.ccr.2013.01.005.

[20] R. A. Meurer , S. Kemper , S. Knopp , et al., “Biofunctional Microgel‐Based Fertilizers for Controlled Foliar Delivery of Nutrients to Plants,” Angewandte Chemie International Edition 56, no. 26 (2017): 7380–7386, 10.1002/anie.201701620. 28524364

[21] Q. Zhang , D. Tang , J. Zhang , et al., “Self‐Healing Heterometallic Supramolecular Polymers Constructed by Hierarchical Assembly of Triply Orthogonal Interactions with Tunable Photophysical Properties,” Journal of the American Chemical Society 141, no. 44 (2019): 17909–17917, 10.1021/jacs.9b09671. PMC7001772 31617714

[22] C. M. Brown , D. J. Lundberg , J. R. Lamb , et al., “Endohedrally Functionalized Metal–Organic Cage‐Cross‐Linked Polymer Gels as Modular Heterogeneous Catalysts,” Journal of the American Chemical Society 144, no. 29 (2022): 13276–13284, 10.1021/jacs.2c04289. 35819842

[23] X. Yan , D. Xu , X. Chi , et al., “A Multiresponsive, Shape‐Persistent, and Elastic Supramolecular Polymer Network Gel Constructed by Orthogonal Self‐Assembly,” Advanced Materials 24, no. 3 (2011): 362–369, 10.1002/adma.201103220. 22161963

[24] J. Zhao , Y. Zhang , Z. Wang , and D. Yang , “Incorporation of Cages into Gels: Access to a New Class of Soft Materials with Well‐Defined Functionality,” Chemistry – A European Journal 31, no. 17 (2025): 202404363, 10.1002/chem.202404363. 39876063

[25] H. Xu , T. K. Ronson , A. W. Heard , et al., “A Pseudo‐cubic Metal–organic Cage with Conformationally Switchable Faces for Dynamically Adaptive Guest Encapsulation,” Nature Chemistry 17, no. 2 (2025): 289–296, 10.1038/s41557-024-01708-5. PMC11794150 39779971

[26] X. Z. Wang , D. W. Chen , F. Fadaei‐Tirani , X. P. Zhou , and K. Severin , “Selective Recognition and Extraction of Short‐Chain Perfluoroalkyl Carboxylates by a Supramolecular Receptor,” Angewandte Chemie International Edition 64, no. 22 (2025): 202504880, 10.1002/anie.202504880. 40126862

[27] A. B. Sainaba , M. Venkateswarulu , P. Bhandari , K. S. A. Arachchige , J. K. Clegg , and P. S. Mukherjee , “An Adaptable Water‐Soluble Molecular Boat for Selective Separation of Phenanthrene from Isomeric Anthracene,” Journal of the American Chemical Society 144, no. 16 (2022): 7504–7513, 10.1021/jacs.2c02540. 35436087

[28] M. Ray , S. Krishnaswamy , A. K. Pradhan , and D. K. Chand , “Radical Initiators in the Nanoconfinement Paradise of a Molecular Prism,” Chemistry of Materials 35, no. 17 (2023): 6702–6712, 10.1021/acs.chemmater.3c00749.

[29] S. Sato , M. Ikemi , T. Kikuchi , S. Matsumura , K. Shiba , and M. Fujita , “Bridging Adhesion of a Protein onto an Inorganic Surface Using Self‐Assembled Dual‐Functionalized Spheres,” Journal of the American Chemical Society 137, no. 40 (2015): 12890–12896, 10.1021/jacs.5b06184. 26190770

[30] C.‐S. Kwan , A. Walther , A. S. Mikherdov , J. J. Holstein , C. Drechsler , and G. H. Clever , “Dual Cation/Anion Binding in Crown Ether‐Based Coordination Cages,” Journal of the American Chemical Society 148, no. 3 (2025): 3230–3239, 10.1021/jacs.5c17231. 41422546

[31] R. Singha , P. De , and D. Samanta , “Solvent‐Induced Self‐Assembly of Pd_6_L_3_ and Pd_8_L_4_ Coordination Cages for Benzene–Cyclohexane Separation,” Inorganic Chemistry 64, no. 9 (2025): 4367–4377, 10.1021/acs.inorgchem.4c04965. 40009737

[32] D. Samanta , D. Galaktionova , J. Gemen , et al., “Reversible Chromism of Spiropyran in the Cavity of a Flexible Coordination Cage,” Nature Communications 9, no. 1 (2018): 641, 10.1038/s41467-017-02715-6. PMC5811438 29440687

[33] J. L. Zhu , D. Zhang , T. K. Ronson , et al., “A Cavity‐Tailored Metal‐Organic Cage Entraps Gases Selectively in Solution and the Amorphous Solid State,” Angewandte Chemie International Edition 60, no. 21 (2021): 11789–11792, 10.1002/anie.202102095. PMC8251750 33768657

[34] M. R. Black , S. Bhattacharyya , S. P. Argent , and B. S. Pilgrim , “Structural Transformations of Metal–Organic Cages through Tetrazine‐Alkene Reactivity,” Journal of the American Chemical Society 146, no. 41 (2024): 28233–28241, 10.1021/jacs.4c08591. PMC11487605 39236092

[35] S. C. Wei , M. Pan , Y. Z. Fan , H. Liu , J. Zhang , and C. Y. Su , “Creating Coordination‐Based Cavities in a Multiresponsive Supramolecular Gel,” Chemistry – A European Journal 21, no. 20 (2015): 7418–7427, 10.1002/chem.201406517. 25876958

[36] S. Ganta and D. K. Chand , “Multi‐Stimuli‐Responsive Metallogel Molded from a Pd_2_L_4_‐Type Coordination Cage: Selective Removal of Anionic Dyes,” Inorganic Chemistry 57, no. 7 (2017): 3634–3645, 10.1021/acs.inorgchem.7b02239. 29064678

[37] S. Bera , A. Dutta , and P. Dastidar , “Developing Supramolecular Metallogel Derived from Pd_2_L_4_ Cage Molecule for Delivering an Anti‐Cancer Drug to Melanoma Cell B_16_−F_10_ ,” Chemistry – An Asian Journal 19, no. 17 (2024): 202400419, 10.1002/asia.202400419. 38872363

[38] M. Dalapati , R. Singha , P. Maity , D. Manna , and D. Samanta , “Self‐Assembled Pd(II) Nano‐Adsorbents for Iodine and Methyl Iodide Capture in Vapor and Aqueous Phases,” Small 21, no. 33 (2025): 2504242, 10.1002/smll.202504242. PMC12372449 40583392

[39] Y. Ma , J. Pan , H. Rong , et al., “Porous Aromatic Framework with Multifunctional Sites for Effective Recovery of Various Trace Iodine Species from Water,” Advanced Science 12, no. 17 (2025): 2500993, 10.1002/advs.202500993. PMC12061248 40047336

[40] A. Saiz‐Lopez , J. M. C. Plane , A. R. Baker , et al., “Atmospheric Chemistry of Iodine,” Chemical Reviews 112, no. 3 (2011): 1773–1804, 10.1021/cr200029u. 22032347

[41] W. Zhou , A. Li , M. Zhou , Y. Xu , Y. Zhang , and Q. He , “Nonporous Amorphous Superadsorbents for Highly Effective and Selective Adsorption of Iodine in Water,” Nature Communications 14, no. 1 (2023): 5388, 10.1038/s41467-023-41056-5. PMC10477329 37666841

[42] X. Zhang , J. Maddock , T. M. Nenoff , M. A. Denecke , S. Yang , and M. Schröder , “Adsorption of Iodine in Metal–organic Framework Materials,” Chemical Society Reviews 51, no. 8 (2022): 3243–3262, 10.1039/d0cs01192d. PMC9328120 35363235

[43] L. Wang , R. Guo , L. Song , et al., “Recent Progress in Covalent Organic Framework‐based Adsorption for Iodine Capture,” Separation and Purification Technology 377 (2025): 134386, 10.1016/j.seppur.2025.134386.

[44] M.‐Z. Zhuo , X.‐Y. Yan , Z.‐H. Yang , Z.‐Y. Chen , X.‐H. Wang , and M.‐X. Wu , “Hydrogen‐bonded Organic Frameworks Leveraging Lewis Basic Motifs for High‐capacity Iodine Capture,” Chemical Communications 61, no. 84 (2025): 16416–16419, 10.1039/d5cc03745j. 40990565

[45] A. Hazra and S. K. Samanta , “Fabricating Tetraphenylethylene‐Based Ionic Porous Organic Polymers for Efficient Sequestration of Toxic Iodine and Oxoanions in Multiple Media,” ACS Applied Materials & Interfaces 17, no. 14 (2025): 21281–21294, 10.1021/acsami.5c01579. 40131737

[46] R. Singha , R. Pradhan , P. De , et al., “Dual Capture of Iodine and Methyl Iodide Using Nonporous Nitrogen‐Enriched Palladium(II) Assemblies,” Inorganic Chemistry 64, no. 3 (2025): 11586–11599, 10.1021/acs.inorgchem.5c00972. 40461979

[47] R. Singha , R. Pradhan , M. Dalapati , P. De , D. Manna , and D. Samanta , “Microporous Self‐Assembled Pd(II) Tetrahedral Cages for Rapid and Reversible Multi‐Phase Sequestration of Iodine and Methyl Iodide,” Small 22, no. 35 (2026): 73642, 10.1002/smll.73642. PMC13288786 42107109

[48] G. Matthys , A. Laemont , N. De Geyter , R. Morent , R. Lavendomme , and P. Van Der Voort , “Robust Imidazopyridinium Covalent Organic Framework as Efficient Iodine Capturing Materials in Gaseous and Aqueous Environment,” Small 20, no. 47 (2024): 2404994, 10.1002/smll.202404994. 39169707

[49] M. Zhang , J. Samanta , B. A. Atterberry , R. Staples , A. J. Rossini , and C. Ke , “A Crosslinked Ionic Organic Framework for Efficient Iodine and Iodide Remediation in Water,” Angewandte Chemie 134, no. 52 (2022): 202214189, 10.1002/ange.202214189. 36331335

[50] L. Xiao , H. Zhao , F. Fu , et al., “Metal–Organic Cages for Ultrafast Photocatalytic Synthesis of Hydrogen Peroxide,” Angewandte Chemie International Edition 64, no. 47 (2025): 202516678, 10.1002/anie.202516678. 40996345

[51] G. Li , Z. Du , C. Wu , et al., “Charge‐transfer Complexation of Coordination Cages for Enhanced Photochromism and Photocatalysis,” Nature Communications 16, no. 1 (2025): 546, 10.1038/s41467-025-55893-z. PMC11718061 39789017

[52] K. Lu , C. He , and W. Lin , “Nanoscale Metal–Organic Framework for Highly Effective Photodynamic Therapy of Resistant Head and Neck Cancer,” Journal of the American Chemical Society 136, no. 48 (2014): 16712–16715, 10.1021/ja508679h. PMC4277757 25407895

[53] P. K. Maitra , S. Bhattacharyya , N. Hickey , and P. S. Mukherjee , “Self‐Assembly of a Water‐Soluble Pd_16_ Square Bicupola Architecture and Its Use in Aerobic Oxidation in Aqueous Medium,” Journal of the American Chemical Society 146, no. 22 (2024): 15301–15308, 10.1021/jacs.4c02956. 38785321

[54] Y. Xu , W. Tuo , L. Yang , et al., “Design of a Metallacycle‐Based Supramolecular Photosensitizer for in Vivo Image‐Guided Photodynamic Inactivation of Bacteria,” Angewandte Chemie International Edition 61, no. 5 (2021): 202110048, 10.1002/anie.202110048. 34806264

[55] J. Luo , J. Lu , and J. Zhang , “Carbazole–triazine Based Donor–acceptor Porous Organic Frameworks for Efficient Visible‐light Photocatalytic Aerobic Oxidation Reactions,” Journal of Materials Chemistry A 6, no. 31 (2018): 15154–15161, 10.1039/c8ta05329d.

[56] R. Sun , X. Hu , X. Yang , et al., “Carbazole‐containing Covalent Triazine Frameworks for Efficient Hydrogen Peroxide Photosynthesis from Natural Sunlight,” Chemical Engineering Journal 490 (2024): 151332, 10.1016/j.cej.2024.151332.

[57] X. Lang , J. Zhao , and X. Chen , “Visible‐Light‐Induced Photoredox Catalysis of Dye‐Sensitized Titanium Dioxide: Selective Aerobic Oxidation of Organic Sulfides,” Angewandte Chemie International Edition 55, no. 15 (2016): 4697–4700, 10.1002/anie.201600405. 26969891

[58] Y. Han , K. Xing , J. Zhang , et al., “Application of Sulfoximines in Medicinal Chemistry from 2013 to 2020,” European Journal of Medicinal Chemistry 209 (2021): 112885, 10.1016/j.ejmech.2020.112885. 33227576

[59] Z. Chen , X.‐W. Deng , X.‐Y. Wang , A.‐Q. Wang , and W.‐T. Luo , “Carbazole‐based Aggregation‐induced Phosphorescent Emission‐active Gold(I) Complexes with Various Phosphorescent Mechanochromisms,” Frontiers in Chemistry 10 (2022): 1083757, 10.3389/fchem.2022.1083757. PMC9752811 36531308

[60] H. Liu , D.‐D. Ren , X.‐L. Zhu , Y.‐P. Wu , and H.‐R. Fu , “Coordination‐driven Stacking of Carbazole‐based Molecule for Dynamic Long‐lived Room Temperature Phosphorescence,” Journal of Solid State Chemistry 323 (2023): 123983, 10.1016/j.jssc.2023.123983.

[61] I. Pal , S. Ghosh , D. Saha , et al., “Organic and Coordination Polymer‐Based Gelators‐Directed Mechanically Self‐Repairing Flexible Metallosupramolecular Soft Scaffolds,” Inorganic Chemistry 64, no. 12 (2025): 5971–5985, 10.1021/acs.inorgchem.4c04986. 40096708

[62] M. Fahad , M. A. Khan , and M. Gilbert , “Investigation of Thermal Gel Formation of Methylcellulose in Glycols Using DSC and XRD,” Gels 7, no. 4 (2021): 205, 10.3390/gels7040205. PMC8628798 34842671

[63] K. Takata , K. Kawahara , Y. Yoshida , A. Kuzuya , and Y. Ohya , “Analysis of the Sol‐to‐gel Transition Behavior of Temperature‐responsive Injectable Polymer Systems by Fluorescence Resonance Energy Transfer,” Polymer Journal 49, no. 9 (2017): 677–684, 10.1038/pj.2017.33.

[64] H. M. N. Ahmad , G. Dutta , J. Csoros , et al., “Stimuli‐Responsive Templated Polymer as a Target Receptor for a Conformation‐Based Electrochemical Sensing Platform,” ACS Applied Polymer Materials 3, no. 1 (2020): 329–341, 10.1021/acsapm.0c01120. PMC7971449 33748761

[65] V. R. de la Rosa , W. Nau , and R. Hoogenboom , “Thermoresponsive Interplay of Water Insoluble Poly(2‐alkyl‐2‐oxazoline)s Composition and Supramolecular Host–Guest Interactions,” International Journal of Molecular Sciences 16, no. 4 (2015): 7428–7444, 10.3390/ijms16047428. PMC4425026 25849653

[66] M. Zhang , D. Xu , X. Yan , et al., “Self‐Healing Supramolecular Gels Formed by Crown Ether Based Host–Guest Interactions,” Angewandte Chemie International Edition 51, no. 28 (2012): 7011–7015, 10.1002/anie.201203063. 22653895

[67] C. Cheng , X. Zhang , Y. Meng , Z. Zhang , J. Chen , and Q. Zhang , “Multiresponsive and Biocompatible Self‐healing Hydrogel: Its Facile Synthesis in Water, Characterization and Properties,” Soft Matter 13, no. 16 (2017): 3003–3012, 10.1039/c7sm00350a. 28367574

[68] Z. Zheng , Q. Lin , Y. Gao , X. Ji , J. L. Sessler , and H. Wang , “Recent Advances in Iodine Adsorption from Water,” Coordination Chemistry Reviews 511 (2024): 215860, 10.1016/j.ccr.2024.215860.

[69] S. T. Wang , Y. J. Liu , C. Y. Zhang , F. Yang , W. H. Fang , and J. Zhang , “Cluster‐Based Crystalline Materials for Iodine Capture,” Chemistry – A European Journal 29 (2023): 202202638, 10.1002/chem.202202638. 36180419

[70] S. Fajal , W. Mandal , A. Torris , et al., “Ultralight Crystalline Hybrid Composite Material for Highly Efficient Sequestration of Radioiodine,” Nature Communications 15, no. 1 (2024): 1278, 10.1038/s41467-024-45581-9. PMC10858966 38341406

[71] J. L. Lambert , G. L. Hatch , and B. Mosier , “Iodide and Iodine Determination in the Parts‐per‐billion Range with Leuco Crystal Violet and N‐chlorosuccinimide‐succinimide Reagents,” Analytical Chemistry 47, no. 6 (1975): 915–916, 10.1021/ac60356a010.

[72] S. Maji and R. Natarajan , “A Halogen‐Bonded Organic Framework (XOF) Emissive Cocrystal for Acid Vapor and Explosive Sensing, and Iodine Capture,” Small 19, no. 44 (2023): 2302902, 10.1002/smll.202302902. 37394720

[73] K. Junthod , B. Todee , K. Khamphaijun , et al., “Halogen‐Bonding Interaction‐Mediated Efficient Iodine Capture of Highly Nitrogen‐Functionalized Hyper‐Crosslinked Polymers,” ACS Applied Polymer Materials 6, no. 12 (2024): 7124–7136, 10.1021/acsapm.4c00931.

[74] Y. Xie , T. Pan , Q. Lei , et al., “Efficient and Simultaneous Capture of Iodine and Methyl Iodide Achieved by a Covalent Organic Framework,” Nature Communications 13, no. 1 (2022): 2878, 10.1038/s41467-022-30663-3. PMC9130143 35610232

[75] M. Dalapati , A. Das , P. Maity , R. Singha , S. Ghosh , and D. Samanta , “N‐Heteroatom Engineered Nonporous Amorphous Self‐Assembled Coordination Cages for Capture and Storage of Iodine,” Inorganic Chemistry 63, no. 34 (2024): 15973–15983, 10.1021/acs.inorgchem.4c02343. 39140114

[76] K. Cheng , H. Li , J. R. Wang , P. Z. Li , and Y. Zhao , “From Supramolecular Organic Cages to Porous Covalent Organic Frameworks for Enhancing Iodine Adsorption Capability by Fully Exposed Nitrogen‐Rich Sites,” Small 19, no. 34 (2023): 2301998, 10.1002/smll.202301998. 37162443

[77] E. D. Miensah , J. Chen , A. Gu , et al., “Ultrahigh Capture of Radioiodine with Zinc Oxide‐decorated, Nitrogen‐doped Hierarchical Nanoporous Carbon Derived from Sonicated ZIF‐8‐precursor,” Journal of Materials Science 56, no. 15 (2021): 9106–9121, 10.1007/s10853-021-05887-1.

[78] Y. Xie , T. Pan , Q. Lei , et al., “Ionic Functionalization of Multivariate Covalent Organic Frameworks to Achieve an Exceptionally High Iodine‐Capture Capacity,” Angewandte Chemie 133, no. 41 (2021): 22606–22614, 10.1002/ange.202108522. 34431190

[79] Z.‐Y. Zeng , Z.‐C. Lou , L. Hu , et al., “Metal‐organic Cages for Efficient Capture and Convenient Detection of Iodine from Vapor and Aqueous Solutions,” Chemical Engineering Journal 496 (2024): 154091, 10.1016/j.cej.2024.154091.

[80] P. Chen , X. He , M. Pang , X. Dong , S. Zhao , and W. Zhang , “Iodine Capture Using Zr‐Based Metal–Organic Frameworks (Zr‐MOFs): Adsorption Performance and Mechanism,” ACS Applied Materials & Interfaces 12, no. 18 (2020): 20429–20439, 10.1021/acsami.0c02129. 32255599

[81] A. Jati , S. Dam , T. S. Khan , and B. Maji , “A Robust Nickel‐Interlocked π‐Conjugated Covalent Organic Framework Catalyst for Photocatalytic Aromatic Finkelstein and Retro‐Finkelstein Reactions,” Angewandte Chemie International Edition 64, no. 36 (2025): 202510788, 10.1002/anie.202510788. 40608012

[82] X. Zhan , Y. Jin , C. Qu , et al., “Synergistic Strategy toward Enhancing Photosynthesizing Reactive Oxygen Species of Covalent Organic Frameworks,” Advanced Functional Materials 35, no. 8 (2024): 2415629, 10.1002/adfm.202415629.

[83] X. Liu , C. Si , J. Xu , H. Sun , J. Li , and Q. Han , “Constructing a Polyoxometalate‐Based Metal–Organic Framework for Photocatalytic Oxidation of Thioethers to Sulfoxides Utilizing in Situ‐Generated Superoxide Radicals,” Inorganic Chemistry 64, no. 3 (2025): 1263–1271, 10.1021/acs.inorgchem.4c03219. 39812524

[84] G. K. Dam , V. Jaiswal , S. Let , et al., “Ultrafast Photocatalytic Decontamination of Mustard Gas Simulant and Thermodynamic Insights by a Metal‐Free Imidazoline Porous Organic Polymer,” Chemistry of Materials 37, no. 5 (2025): 1854–1864, 10.1021/acs.chemmater.4c02959.

[85] Z. Chen , L. Ding , X. Huang , et al., “Self‐assembly of Azulene‐based Metallacycles for Efficient Sulfide Photooxidation,” Inorganic Chemistry Communications 177 (2025): 114407, 10.1016/j.inoche.2025.114407.

[86] T. Huang , J. Kou , H. Yuan , et al., “Linker Modulation of Covalent Organic Frameworks at Atomic Level for Enhanced and Selective Photocatalytic Oxidation of Thioether,” Advanced Functional Materials 35, no. 4 (2024): 2413943, 10.1002/adfm.202413943.

[87] X. Bai , X. Han , Y. Wang , et al., “Two 3D Two‐Fold Interpenetrated Dia‐Like Polyoxometalate‐Based Metal–Organic Frameworks: Synthesis and Sulfide Selective Oxidation Activity,” Inorganic Chemistry 62, no. 33 (2023): 13221–13229, 10.1021/acs.inorgchem.3c01393. 37552041

[88] F. Shi , M. K. Tse , H. M. Kaiser , and M. Beller , “Self‐Catalyzed Oxidation of Sulfides with Hydrogen Peroxide: a Green and Practical Process for the Synthesis of Sulfoxides,” Advanced Synthesis & Catalysis 349, no. 16 (2007): 2425–2430, 10.1002/adsc.200700206.

