# Supplementary material for: Self‐Healing Pd(II) Cage Metallogels for Iodine Capture in Water and Heterogeneous Photocatalysis
Source: Adv Sci (Weinh). 2026 Sep 29:e78042. Online ahead of print. doi: 10.1002/advs.78042 (PMC13624423; doi:10.1002/advs.78042)
Supplement: Supplementary file 1 [file ADVS-9999-e78042-s001.docx]

**Self-Healing Pd(II) Cage Metallogels for Iodine Capture in Water and Heterogeneous Photocatalysis**

*Monotosh Dalapati,^a,b^ Raghunath Singha,^a,b^ Debashree Manna,^c^ Dipak Samanta* ^a,b,d^*

^a^School of Chemical Sciences, National Institute of Science Education and Research (NISER), Bhubaneswar, PO Bhimpur- Padanpur, Via Jatni, District Khurda, Odisha 752050, India.

^b^Homi Bhabha National Institute, Training School Complex, Anushakti Nagar, Mumbai 400094, India

^c^Institute of Organic Chemistry and Biochemistry, Czech Academy of Sciences, v.v.i., Flemingovo nám. 2, Prague 6, Praha 16610, Czech Republic

^d^Center for Interdisciplinary Sciences (CIS), National Institute of Science Education and Research (NISER), An OCC of Homi Bhabha National Institute (HBNI), PO Bhimpur- Padanpur, Via Jatni, District Khurda, Odisha 752050, India.

E-mail: [dsamanta@niser.ac.in](mailto:dsamanta@niser.ac.in)

**Table of Content**

1. **General Method** …………….…………...…………….…………………………….**S3**
2. **Synthesis and Characterization**………………………………...…………………..**S5**
3. **X-ray Data Collection and Structure Refinement**………………………….….....**S38**
4. **Gas Sorption Isotherm** ……...……………………………………………….....….**S41**
5. **FE-SEM Image** ………………………………...……………………………….….**S42**
6. **EDAX Analysis** ………………………...…………………………………...….…..**S43**
7. **Gel Preparation and Characterization** …………………………………….....…..**S45**
8. **Assessment of Self-healing Properties**………………………………………….....**S62**
9. **Static Adsorption of Iodine from Aqueous Media**.…………………………..…..**S66**
10. **Dynamic Adsorption of Iodine from Aqueous Solution**………..….…………......**S90**
11. **Adsorption of Trace Aqueous Iodine** ………….….………………………….…...**S92**
12. **Investigation of the Absorption Mechanism** …………………..….……..…….....**S93**
13. **DFT Calculations** …………………………………………………………............**S102**
14. **Iodine Adsorption Capacities of Selected Materials in Aqueous Solution** ........**S107**
15. **Iodine Adsorption Kinetics across Various Material** ………………..…...….....**S108**
16. **Photocatalytic Oxidation Reaction**…………………………………………....…**S109**
17. **Reusability of the Photocatalytic Material** …………………………….…....…..**S116**
18. **Fluorescence quenching experiment** ……………………………..…….……......**S116**
19. **Time-Dependent Reaction Kinetics and Selectivity** ……………………………**S117**
20. **Comparison of TOFs for Photocatalytic Oxidation Catalysts** ……………….. **S136**
21. **Conversion Efficiency Comparison for Photocatalytic Sulfoxidation** .………..**S137**
22. **Comparison of Reported Metallocage based Gels and their Applications**...….**S141**
23. **Analytical Data for Synthesized Products** ..………………………………...…..**S142**
24. **References** …………………………………………………………….………......**S162**

**1**. **General Method**

**Materials and General Procedure**: Commercially available reagents and solvents were used as received without further purifications. The compounds were purified by column chromatography on silica gel (mesh 230−400). NMR spectra were recorded on Bruker Avance III 400 MHz or 700 MHz instruments at 298 K. Chemical shifts (*δ*) are given in ppm relative to residual protio solvent resonances (7.26 ppm for CDCl_3_, 3.31 ppm for CD_3_OD and 2.50 for DMSO-d_6_). The diffusion coefficient of the solvent was used as a calibration standard. Unless otherwise specified, all measurements were conducted at 298 K. High-resolution mass spectra (HR-MS) were recorded on an ESI-TOF (time of flight) mass spectrometer. FT-IR spectra were obtained using a Perkin Elmer spectrophotometer with ATR Gold. UV-Vis experiments were conducted on a Perkin Elmer Lamda 365+ UV-Vis spectrophotometer with a 1 nm slit width. XPS was performed using a PHI5000 Versaprobe III XPS. The Raman spectra were acquired on a Lab RAM HR Evolution, Horiba Scientific, Raman Spectrometer, equipped with a 532 nm laser ULF. Thermal stability was assessed using the Discovery TGA-1 TA analyser in N_2_ atmosphere at a heating rate of 10 ^ο^C min^–1^. EPR spectra were obtained with a Bruker System EMX-microX at 298K and 9.4335 GHz. Typical EPR spectrometer parameters are shown as follows, scan range: 100 G; centre field set: 3480.00 G; time constant: 0.16 ms; scan time:128.22 s; modulation amplitude: 20.0 G; modulation frequency: 100 kHz; receiver gain: 2.00 × 102; microwave power: 7.14e^−001^ mW; g = 2.007092. Powder X-ray diffraction (PXRD) patterns were collected at room temperature on a Bruker D8 Advance X-ray powder diffractometer with Cu Kα radiation (λ = 1.5418 Å) as the X-ray source. Scanning electron microscopy (SEM) measurements were conducted with a Merlin Compact field effect SEM (FESEM) with a GEMINI-I electron column, Zeiss Pvt. Ltd., Germany. N_2_ sorption analyses were performed at 77 K on a Quantachrome Instruments Autosorb iQ MP automatic volumetric instrument. Atomic force microscopy (AFM) is employed to understand the nanoscale surface morphology of the prepared samples. The measurements are performed in tapping mode (AC mode imaging) with a silicon probe TESPG V2 using Bruker Nano Wizard Sense+ Bio-AFM. This TESPG V2 probe has a spring constant of ~37 N/m and a resonant frequency of ~320 kHz. TEM analysis was carried out using a JEOL JEM-2100 electron microscope operated at an accelerating voltage of 200 kV. PL spectra were recorded using an Edinburgh FS5 spectrofluorometer. Long-lived fluorescence lifetimes were measured using time-correlated single-photon counting (TCSPC) with an Edinburgh Instruments OB-920 system. Excitation was provided by a diode laser, and emission was collected at the magic angle (54.7°) to minimize polarization effects. The instrument response function (IRF), determined using a scattering Ludox solution, was approximately 90 ps. Fluorescence decay curves were analyzed and fitted using the instrument’s F980 software. Differential scanning calorimetric (DSC) analyses were recorded on a Mettler Toledo, Star ESystem DSC1/700 model in a nitrogen atmosphere at a heating rate of 5 °C min^–1^. Dynamic light scattering (DLS) measurement were performed using a MALVERN ZETASIZER Nano ZS. To access this mechanical strength of the systems, frequency sweep experiments were conducted at a fixed strain of 0.1%. The linear viscoelastic region (LVR) of the gels was determined by initial strain sweep measurements over a strain range of 0.01–100% at a constant oscillatory frequency of 1 Hz by Rheometer Model- TA AR G2 Sl, No.- 11D4672, Maker- TA INSTRUMENTS LTD. Time-dependent mechanical properties were subsequently monitored at a constant strain of 0.1% and a fixed frequency of 1 Hz at 25 °C. Dynamic light scattering measurement (DLS) were performed using a Malvernn Zetasizer Nanno ZS. An Autolab 302 N electrochemical station is used to perform all electrochemical measurements, using glassy carbon (GC) as a working electrode, Pt wire as a counter electrode, and Ag/AgCl (3M KCl) as a reference electrode. ThermoScientific's iCAP 7000 series inductively coupled plasma-optical emission spectroscopy (ICP-OES) was used to quantify the metal leaching. HANNA pH METER edge dedicated pH, 230V was used to determine the pH of iodine solutions under different pH solution. Lyophilizer MODEL NO. HVL- D64 · By HOVERLABS was used for freeze-drying the gel to obtain the corresponding dry gel powder.

**2**. **Synthesis and Characterization**

**3,6-dibromo-9H-carbazole**:^1^

**Scheme S1**. Synthesis of 3,6-dibromo-9H-carbazole.

Carbazole (2.6 g, 15.54 mmol) was dissolved in 10 mL of *N*,*N*-dimethylformamide (DMF) and a solution of *N*-bromosuccinimide (NBS) (6.36 g, 35.76 mmol) in 20 mL of DMF was added dropwise at 0 °C. The mixture was stirred for 2 h, then 150 mL of deionized water was added to precipitate the product. The solid, 3,6-dibromocarbazole, was isolated by vacuum filtration and dried under reduced pressure. Yield: 4.8 g (92%).

^1^H NMR (700 MHz, CD_3_OD): *δ* (ppm) *=* 8.13 (s, 2H), 8.10 (s, 1H), 7.52 (dd, *J* = 8.5, 1.7 Hz, 2H), 7.31 (d, *J* = 8.5 Hz, 2H). ^13^C NMR (176 MHz, CD_3_OD): *δ* (ppm) = 138.37, 129.35, 124.13, 123.30, 112.67, 112.25. HRMS (ESI): C_12_H_7_Br_2_N, [M + H] ^+^ = 326.00 (calculated) found: 325.9852.

**3,6-dibromo-9-methyl-9H-carbazole**:

**Scheme S2**. Preparation of 3,6-dibromo-9-methyl-9H-carbazole.

A mixture of 3,6-dibromo-9H-carbazole (200 mg, 0.615 mmol) and potassium tert-butoxide in THF (5 mL) was heated to 60 °C under reflux. Methyl iodide (46.5 μL, 0.74 mmol) was added dropwise to the reaction mixture and the reaction was continued at 60 °C for an additional 2 h under reflux conditions. The solvent was subsequently removed under reduced pressure, and the addition of water induced precipitation of the product. The resulting solid was collected by filtration, washed with cold methanol, and dried to obtain the pure product. Yield: 280 mg (85%).

^1^H NMR (400 MHz, CDCl_3_): *δ* (ppm) *=* 8.02 (s, 2H), 7.52 (d, *J* = 8.6 Hz, 2H), 7.16 (d, *J* = 8.7 Hz, 2H), 3.70 (s, 3H). ^13^C NMR (101 MHz, CD_3_OD): *δ* (ppm) = δ 139.73, 128.99, 123.25, 123.08, 112.00, 110.08, 29.23. HRMS (ESI): C_13_H_9_Br_2_N, [M + H]^+^ = 340.03 (calculated) found:340.0292.

**3,6-di(1H-imidazol-1-yl)-9-methyl-9H-carbazole**:^2^

**Scheme S3**. Synthesis of ligand **L1**.

In a dry two-necked round-bottom flask equipped with a magnetic stir bar and a reflux condenser, copper(I) iodide (76.41 mg, 0.4 mmol), 1,10-phenanthroline (162.7 mg, 0.9 mmol), and DMF (3 mL) were combined and heated at 120 °C in an oil bath under reflux for 5 min. After cooling the reaction mixture to room temperature, imidazole (1092 mg, 16.04 mmol), potassium tert-butoxide (1807 mg, 16.04 mmol), 3,6-di(1H-imidazol-1-yl)-9-methyl-9H-carbazole (680 mg, 2.0 mmol), and a catalytic amount of 18-crown-6 were added. The resulting mixture was heated at 135 °C and stirred for 36 h. After completion of the reaction, the mixture was cooled to room temperature and extracted with DCM (150 mL). The organic layer was washed three times with distilled water, dried over anhydrous MgSO_4_ and concentrated under reduced pressure using a rotary evaporator. The crude product was purified by recrystallization from ethyl acetate to afford the desired compound. Yield: 387 mg (61.6%).

^1^H NMR (700 MHz, DMSO-*d*_6_): *δ* (ppm) *=* 8.49 (s, 2H), 8.27 (s, 2H), 7.77 (d, *J* = 26.6 Hz, 6H), 7.20 (s, 2H), 3.95 (s, 3H). ^13^C NMR (176 MHz, DMSO-*d*_6_): *δ* (ppm) = 140.51, 136.73, 130.20, 130.04, 122.66, 120.42, 119.67, 113.70, 110.98, 29.90. HRMS (ESI): C_19_H_15_N_5_, [M + H]^+^ = 314.31 (calculated) found: 314.3085.

**9-phenyl-9H-carbazole**:^3^

**Scheme S4**. Synthesis of 9-phenyl-9H-carbazole.

In a 100 mL round-bottom flask equipped with a stir bar, 9H-carbazole (1.00 g, 5.98 mmol), copper iodide (1.16 g, 6.10 mmol), potassium carbonate (1.65 g, 11.96 mmol), and iodobenzene (1.5 g, 7.18 mmol) were combined in dry DMF (50 mL). The reaction mixture was stirred under a nitrogen atmosphere and heated at 150 °C for 24 h. After completion of the reaction, the mixture was cooled to room temperature and the solvent was removed under reduced pressure. The residue was dissolved in DCM (120 mL) and washed with brine (60 mL), followed by three additional extractions with DCM. The combined organic layers were dried over anhydrous MgSO_4_ and concentrated under vacuum. The crude product was purified by column chromatography using petroleum ether/EtOAc (10:1) as the eluent, followed by recrystallization from DCM/hexane (three times) to afford colorless crystals. Yield: 1.2 g (82%).

^1^H NMR (400 MHz, CDCl_3_): *δ* (ppm) *=* 8.21 (d, *J* = 7.7 Hz, 2H), 7.64 (q, *J* = 7.9 Hz, 4H), 7.51 (t, *J* = 6.9 Hz, 1H), 7.47 (d, *J* = 3.5 Hz, 4H), 7.38 – 7.31 (m, 2H). ^13^C NMR (176 MHz, CDCl_3_): *δ* (ppm) = 140.94, 137.75, 129.91, 127.49, 127.19, 125.96, 123.39, 120.34, 119.94, 109.81. HRMS (ESI): C_18_H_13_N, [M + H]^+^ = 244.10 (calculated) found: 244.0978.

**3,6-dibromo-9-phenyl-9H-carbazole**:^4^

**Scheme S5**. Preparation of 3,6-dibromo-9-phenyl-9H-carbazole.

A solution of N-bromosuccinimide (NBS) (4.45 g, 25 mmol) in dichloromethane (20 mL) was added dropwise to a solution of 9-phenyl-9H-carbazole (2.43 g, 10 mmol) in DCM (20 mL) under ice-bath cooling conditions. After complete addition, the reaction mixture was allowed to warm to room temperature and stirred overnight. The solvent was then removed under reduced pressure using a rotary evaporator. The crude product was purified by silica gel column chromatography using petroleum ether as the eluent to afford the desired product as a white solid. Yield: 3.43 g (85.7%).

^1^H NMR (700 MHz, DMSO-*d*_6_): *δ* (ppm) *=* 8.59 (d, *J* = 1.7 Hz, 2H), 7.69 (t, *J* = 7.7 Hz, 2H), 7.61 (d, *J* = 7.6 Hz, 2H), 7.60 – 7.56 (m, 3H), 7.32 (d, *J* = 8.7 Hz, 2H). ^13^C NMR (176 MHz, DMSO-*d*_6_): *δ* (ppm) = 144.49, 141.19, 135.53, 134.69, 133.46, 131.91, 128.92, 128.78, 117.66, 117.08. HRMS (ESI): C_18_H_11_Br_2_N, [M + H]^+^ = 401.92 (calculated) found: 401.9196.

**3,6-di(1H-imidazol-1-yl)-9-phenyl-9H-carbazole** (**L2**):

**Scheme S6**. Synthesis of the ditopic ligand **L2**.

The compound was synthesized following the same procedure used for the preparation of ligand **L1**.

Yield: 515 mg (55%).

^1^H NMR (400 MHz, DMSO-*d*_6_): *δ* (ppm) *=* δ 8.63 (d, *J* = 1.8 Hz, 2H), 8.26 (s, 2H), 7.79 – 7.75 (m, 3H), 7.74 – 7.67 (m, 5H), 7.60 (t, *J* = 7.1 Hz, 1H), 7.51 (d, *J* = 8.8 Hz, 2H), 7.18 (s, 2H). ^13^C NMR (176 MHz, DMSO-*d*_6_): *δ* (ppm) = 139.97, 136.76, 136.38, 131.04, 130.86, 130.19, 128.65, 127.14, 123.54, 121.00, 119.32, 114.06, 111.42. HRMS (ESI): C_24_H_17_N_5_, [M + H]^+^ = 376.14 (calculated) found: 376.1387.

**Figure S1**. ^1^H NMR spectrum of 3,6-dibromo-9H-carbazole in CDCl_3_ (700 MHz, 298 K).

**Figure S2**. ^13^C NMR spectrum of 3,6-dibromo-9H-carbazole in CDCl_3_ (176 MHz, 298 K).

**Figure S3**. ^1^H NMR spectrum of 3,6-dibromo-9-methyl-9H-carbazole in CDCl_3_ (700 MHz, 298 K).

**Figure S4**. ^13^C NMR spectrum of 3,6-dibromo-9-methyl-9H-carbazole in CDCl_3_ (176 MHz, 298 K).

**Figure S5**. ^1^H NMR spectrum of **L1** in DMSO-*d*_6_ (700 MHz, 298 K).

**Figure S6**. ^13^C NMR spectrum of **L1** in DMSO-*d*_6_ (176 MHz, 298 K).

**Figure S7**. ^1^H‒^1^H COSY NMR spectrum of **L1** in DMSO-*d*_6_ (700 MHz, 298 K).

**Figure S8**. ^1^H NMR spectrum of 9-phenyl-9H-carbazole in CDCl_3_ (700 MHz, 298 K).

**Figure S9**. ^13^C NMR spectrum of 9-phenyl-9H-carbazole in DMSO-*d*_6_ (176 MHz, 298 K).

**Figure S10**. ^1^H NMR spectrum of 3,6-dibromo-9-phenyl-9H-carbazole in DMSO-*d*_6_ (700 MHz, 298 K).

**Figure S11**. ^13^C NMR spectrum of 3,6-dibromo-9-phenyl-9H-carbazole in DMSO-*d*_6_ (176 MHz, 298 K).

**Figure S12**. ^1^H NMR spectrum of **L2** in DMSO-*d*_6_ (700 MHz, 298 K).

**Figure S13**. ^13^C NMR spectrum of **L2** in DMSO-*d*_6_ (176 MHz, 298 K).

**Figure S14**. ^1^H‒^1^H COSY NMR spectrum of **L2** in DMSO-*d*_6_ (700 MHz, 298 K).

**General Method for the Preparation of Cages (MOC1 and MOC2)**

A 2 mL clear DMSO solution of Pd(NO_3_)_2_ was added separately to each ligand (**L1** and **L2**), and the mixtures were stirred at room temperature for 1 h. After the reaction, the resulting brownish solutions were centrifuged to obtain clear supernatants. Excess ethyl acetate was then added, inducing the formation of white precipitates, which were collected and washed three times with diethyl ether.

**MOC1**: Solid **L1** (45.0 mg, 0.145 mmol) was treated with a DMSO solution of Pd(NO_3_)_2_ (16.54 mg, 0.062 mmol). Yield = 43.2 mg, ⁓70%.

^1^H NMR (400 MHz, DMSO-*d*_6_): *δ* (ppm) *=* 8.97 (s, 8H), 8.64 (s, 8H), 8.05 (s, 8H), 7.84 (d, *J* = 8.9 Hz, 8H), 7.80 (d, *J* = 9.2 Hz, 8H), 7.42 (s, 8H), 3.94 (s, 12H). ^13^C NMR (176 MHz, DMSO-*d*_6_): *δ* (ppm) = 141.34, 138.50, 129.19, 128.79, 122.18, 122.09, 121.46, 115.02, 111.48, 30.05. ESI-MS (DMSO+CH_3_CN): m/z = 795.1578 for [**MOC1**+2(NO_3_)]^2+^ (calc. 795.1451) and 509.447 for [**MOC1**+1(NO_3_)]^3+^ (calc. 509.4284).

**MOC2**: A DMSO solution of Pd(NO_3_)_2_ (15.0 mg, 0.0562 mmol) was added to the solid **L2** (42.27 mg, 0.113 mmol). Yield = 52.7 mg, ⁓95%.

^1^H NMR (400 MHz, DMSO-*d*_6_): *δ* (ppm) *=* 9.46 (s, 8H), 8.96 (s, 8H), 8.12 (s, 8H), 7.74 (d, *J* = 8.5 Hz, 8H), 7.65 – 7.61 (m, 16H), 7.50 (d, *J* = 7.5 Hz, 12H), 7.44 (d, *J* = 8.2 Hz, 8H). ^13^C NMR (176 MHz, DMSO-*d*_6_): *δ* (ppm) = 140.55, 137.36, 136.30, 130.89, 129.67, 129.33, 128.75, 126.67, 123.34, 120.62, 119.73, 114.88, 111.54. ESI-MS (DMSO+CH_3_CN): m/z = 919.1877 for [**MOC2**+2(NO_3_)]^2+^ (calc. 919.3051) and 592.1317 for [**MOC2**+1(NO_3_)]^3+^ (calc. 592.2017).

 **Figure S15**. ^1^H NMR spectrum of **MOC1** in DMSO-*d*_6_ (700 MHz, 298 K).

**Figure S16**. ^13^C NMR spectrum of **MOC1** in DMSO-*d*_6_ (176 MHz, 298 K).

**Figure S17**. ^1^H-^1^H COSY spectrum of **MOC1** in DMSO-*d*_6_ (700 MHz, 298 K).

**Figure S18**. Partial ^1^H-^1^H COSY spectrum of **MOC1** in DMSO-*d*_6_ (700 MHz, 298 K).

**Figure S19**. ^1^H-^1^H NOESY spectrum of **MOC1** in DMSO-*d*_6_ (700 MHz, 298 K).

**Figure S20**. Partial ^1^H-^1^H NOESY spectrum of **MOC1** in DMSO-*d*_6_ (700 MHz, 298 K).

**Figure S21**. ^1^H DOSY NMR spectrum of **MOC1** in DMSO-*d*_6_ (400 MHz, 298 K).

**Figure S22**. ESI-MS spectrum of **MOC1** in DMSO and CH_3_CN mixture.

**Figure S23**. ^1^H NMR spectrum of **MOC2** in DMSO-*d*_6_ (700 MHz, 298 K).

**Figure S24**. ^13^C NMR spectrum of **MOC2** in DMSO-*d*_6_ (176 MHz, 298 K).

**Figure S25**. ^1^H-^1^H COSY spectrum of **MOC2** in DMSO-*d*_6_ (700 MHz, 298 K).

**Figure S26**. Partial ^1^H-^1^H COSY spectrum of **MOC2** in DMSO-*d*_6_ (700 MHz, 298 K).

**Figure S27**. Partial ^1^H-^1^H NOSY spectrum of **MOC2** in DMSO-*d*_6_ (700 MHz, 298 K).

**Figure S28**. ^1^H DOSY NMR spectrum of **MOC2** in DMSO-*d*_6_ (400 MHz, 298 K).

**Figure S29**. ESI-MS spectrum of **MOC2** in DMSO and CH_3_CN mixture.

**3**. **X-ray Data Collection and Structure Refinement**

The structural analysis of the coordination cages (**MOC1** and **MOC2**) was performed using single-crystal X-ray crystallography. Suitable single crystals for X-ray diffraction were selected, coated with crystal oil, and mounted under standard conditions. These crystals data were assigned the accession codes CCDC 2523369 and 2523370 for **MOC1** and **MOC2,** respectively. X-ray diffraction were conducted on a Rigaku Super-Nova dual diffractometer equipped with fine-focus Cu-Kα radiation (λ = 1.54184 Å) and Mo-Kα radiation (λ = 0.71073 Å) and multilayer optics with aluminium filtration. A PILATUS200K detector was used at 100 K. Intensity data were collected, corrected, and absorption corrections were applied using the multi-scan method with SCALE3 ABSPACK in CrysAlisPro 50 software. The single-crystal X-ray diffraction data were processed using Olex2 software,^5^ and structural refinement was conducted via least-squares minimisation in SHELXL.^6,7^ Anisotropic refinement was applied to all non-hydrogen atoms. Hydrogen atoms, except those in water molecules, due to their elongated O–H bond lengths, were placed in idealised geometric positions and refined using a riding model. The electron density corresponding to other disordered solvent molecules was squeezed. Despite multiple attempts, the precise positions of hydrogen atoms in water molecules could not be accurately determined due to weak diffraction.

**Table S1**: Crystallographic table of **MOC1**.

| **Parameters** | **MOC1** |
| --- | --- |
| Formula | C_76_H_60_ N_20_F_24_P_4_Pd_2_O4 |
| *Fw* | 2110.08 |
| Crystal system | Monoclinic |
| Space group | *C*2/c |
| Crystal colour and shape | clear light and Block |
| *a*/Å | 28.281(1) |
| *b*/Å | 13.3701(5) |
| *c*/Å | 27.6974(11) |
| *α*/° | 90 |
| *β*/° | 106.347(4) |
| *γ*/° | 90 |
| *V*/Å^3^ | 10049.6(7) |
| *Z* | 4 |
| *D*_c_/g cm^−3^ | 1.395 |
| *μ* Mo K_α_/mm^−1^ | 0.71073 |
| F000 | 4223.9 |
| *T*/K | 100(10) |
| *θ* max. | 26.976 |
| Total no. of reflections | 63847 |
| Independent reflections | 10857 |
| Observed reflections | 7685 |
| Parameters refined | 596 |
| *R*_1_, *I* > 2*σ*(*I*) | 0.0697 |
| w*R*_2_, *I* > 2*σ*(*I*) | 0.2167 |
| GOF (*F*^2^) | 1.040 |
| CCDC no. | 2523369 |

**Table S2**: Crystallographic table of **MOC2**.

| **Parameters** | **MOC2** |
| --- | --- |
| Formula | C_120_H_68_O_6_N_20_F_18_P_3_Pd_2_ |
| *Fw* | 2533.65 |
| Crystal system | triclinic |
| Space group | $P\bar{1}$ |
| Crystal colour and shape | Clear whiteish and prisam |
| *a*/Å | 14.9208(2) |
| *b*/Å | 15.6049(2) |
| *c*/Å | 16.7955(2) |
| *α*/° | 111.0084(11) |
| *β*/° | 96.121(11) |
| *γ*/° | 116.0403(14) |
| *V*/Å^3^ | 3113.36(9) |
| *Z* | 1 |
| *D*_c_/g cm^−3^ | 1.351 |
| *μ* Cu K_α_/mm^−1^ | 1.54184 |
| F000 | 1275.0 |
| *T*/K | 100 K |
| *θ* max. | 75.541 |
| Total no. of reflections | 38721 |
| Independent reflections | 12596 |
| Observed reflections | 11971 |
| Parameters refined | 763 |
| *R*_1_, *I* > 2*σ*(*I*) | 0.0636 |
| w*R*_2_, *I* > 2*σ*(*I*) | 0.1936 |
| GOF (*F*^2^) | 1.040 |
| CCDC no. | 2523370 |

**4**. **Gas Sorption Isotherm**

**Figure S30**. Nitrogen sorption isotherms of the two cages.

**Figure S31**. Pore size distributions of the coordination cages (a) **MOC1** and (b) **MOC2**.

**Table S3**. Summary of the surface area, pore size and pore volume of the cages

| Cage | Surface Area  (m^2^ g) | Pore Volume  (cm^3^ g^−1^) | Pore Diameter  (Å) |
| --- | --- | --- | --- |
| **MOC1** | 42.301 | 0.033 | 15.27 |
| **MOC2** | 74.442 | 0.066 | 17.10 |

**5**. **FE-SEM Image**

**Figure S32**. FE-SEM image of the cages (a, b) **MOC1**, and (c, d) **MOC2**.

**6**. **EDAX Profile for the Cages**

**Figure S33**. FE-SEM-based EDAX elemental analysis of **MOC1** (top) and the corresponding EDS elemental mapping of the **MOC1** cage (bottom).

**Figure S34**. EDAX elemental analysis of **MOC2** (top) and the corresponding EDS elemental mapping images (bottom).

**7**. **Gel Preparation and Characterization**

**Preparation of the Gel**

Ligand **L1**/**L2** (0.026 mmol ) was dissolved in DMSO (0.3 mL) in a 3 mL glass vial to obtain a clear solution. An aqueous solution of Pd(NO_3_)_2_ (3.35 mg, 0.013 mmol in 0.2 mL water), maintaining a metal-to-ligand ratio of 1:2, was added dropwise to the ligand solution with intermittent shaking followed by standing. Repeated shaking and standing of the mixture resulted in complete gelation within 2 h. The formation of the metallogels (**MOC1G**/**MOC2G**) at a concentration of 2.2% w/v was confirmed by the vial inversion test and rheological measurements.

**Rheology Measurement**

The hydrogel was heated until a homogeneous solution was obtained and subsequently transferred onto the Peltier stage. Measurement of the storage modulus (*G*′) and loss modulus (*G*″) was carried out after the cooling of the sample to 25 °C. In the strain sweep, the *G*′ and *G*″ of the hydrogel samples were measured as a function of strain in the range of 0.01 to 100% at a constant angular frequency (ω = 10 rad/s). In the frequency sweep, the *G*′ and *G*″ of the hydrogel samples were measured as a function of angular frequency in the range of 0.1 to 100 rad/s at a constant strain (γ_0_ = 0.1%).^8^

**Preparation of Freeze-Dried Metallogel Powder**

The metallogel was prepared at a concentration of 2.2% (w/v) in DMSO/H₂O solvent mixture. To remove DMSO, the resulting gel was subjected to solvent exchange with deionized water by washing it five times. The water-exchanged gel was subsequently freeze-dried using a lyophilizer to obtain the corresponding dry gel powder.

**Table S4**: Gelation ability of the compounds in various solvents (C_L_ = 2.2% w/v).

**Figure S35**. Photographs of the control experiments showing that the individual ligands (**L1** and **L2**) do not undergo gelation in a DMSO/H_2_O (3:2, v/v) solvent mixture.

**Figure S36**. ^1^H NMR spectrum of **MOC1G** in DMSO-*d*_6_ (400 MHz, 298 K).

 **Figure S37**. ^1^H NMR spectrum of **MOC2G** in DMSO-*d*_6_ (400 MHz, 298 K).

**Figure S38**. ^1^H DOSY NMR spectrum of **MOC1G** in DMSO-*d*_6_ (400 MHz, 298 K).

**Figure S39**. ^1^H DOSY NMR spectrum of **MOC2G** in DMSO-*d*_6_ (400 MHz, 298 K).

**Figure S40**. ESI-MS spectrum of **MOC1G** in a DMSO/CH_3_CN mixture (inset: isotopic distribution for [**MOC1G** + 1NO_3_]^3+^.

**Figure S41**. ESI-MS analysis of **MOC2G** recorded in DMSO/CH_3_CN, with the inset highlighting isotopic pattern of [**MOC2G** + 2NO_3_]^2+^.

**Figure S42**. Variable-temperature ^1^H NMR spectra (400 MHz) of **MOC1G**.

**Figure S43**. Variable-temperature ^1^H NMR spectra (400 MHz) of **MOC2G**.

**Figure S44**. Comparative FT-IR spectra of the corresponding ligand (**L**, green) and the gel states of MOCs.

**Figure S45**. EDAX elemental analysis of **MOC1G** (top) and the corresponding EDS elemental mapping images (bottom).

**Figure S46**. EDAX elemental analysis of **MOC2G** (top) along with the corresponding EDS elemental mapping images (bottom).

**Figure S47**. Differential scanning calorimetry (DSC) thermograms of metallogels **MOC1G** and **MOC2G** showing temperature-dependent thermal transitions.

**Figure S48**. DLS measurements of **MOC1G** at different temperatures showing temperature-dependent variations in particle size.

**Figure S49**. FE-SEM micrographs and corresponding fiber width distribution histograms of the metallogels **MOC1G** and **MOC2G**.

**Figure S50**. AFM-derived height profiles of the metallogels **MOC1G** and **MOC2G**.

**Figure S51**. Time-resolved FT-IR spectra monitoring in situ formation of the **MOC1G** gel upon addition of ligand **L1** to the Pd(II) acceptor.

**Figure S52**. Time-resolved FT-IR spectra monitoring in-situ formation of **MOC2G** gel upon addition of ligand **L2** to the Pd(II) acceptor.

**Figure S53**. Comparative TGA profiles of (a) **MOC1** and **MOC1G** and (b) **MOC2** and **MOC2G**.

**Figure S54**. Strain amplitude sweep of **MOC1G** showing storage (G′) and loss (G″) moduli of the metallogel as a function of applied strain (γ).

**Figure S55**. Oscillatory frequency sweep measurements of **MOC1G** showing the variation of storage modulus (G′) and loss modulus (G″) with angular frequency (ω).

**8**. **Assessment of Self-healing Properties**

A circular specimen (diameter ≈ 1.8 cm; height ≈ 0.4 cm) was completely was completely cut using a razor blade. The freshly cut surfaces were carefully brought back into contact and allowed to heal at 25°C without applying any external pressure. Complete healing was evidenced by the disappearance of the cut mark within a few minutes.

The self-healing properties of the metallogels were quantitatively evaluated using rheological measurements. Metallogels (2.2% w/v) were prepared by mixing ligand (**L1** or **L2**) with Pd(NO_3_)_2_ in a DMSO/water mixture (3:2 v/v). The linear viscoelastic region of the metallogel was first determined through oscillatory shear experiments. At 25 °C, the strain was varied from 0.1 to 50% while maintaining a constant angular frequency (ω = 10 rad s^−1^). For the self-healing experiments, the strain was varied according to the following protocol. γ_1_ = 0.1%, *t*_1_ = 20 s → γ_2_ = 50%, *t*_2_ = 20 s → γ_3_ = 0.1%, *t*_3_ = 20 s → γ_4_ = 50%, *t*_4_ = 20 s → γ_5_ = 0.1%, t_5_ = 20 s → γ_6_ = 50%, t_6_ = 20 s → γ_7_ = 0.1%, *t*_7_ = 20 s.^9^

**Figure S56**. Step-strain rheological analysis demonstrating thixotropic and self-healing behavior of the **MOC1G** under alternating low (0.1%) and high (50%) strain conditions.

**Figure S57**. Macroscopic visualization of the self-healing capability of **MOC1G** following mechanical disruption.

**Figure S58**. Oscillatory frequency sweep measurements of **MOC1G** showing storage modulus G′ and loss modulus G″ as a function of angular frequency ($\omega$) across three damage-healing cycles.

**Figure S59**. Strain amplitude sweep measurements of **MOC1G**, showing the storage (G′) and loss (G″) moduli as a function of applied strain (γ) over three consecutive damage–healing cycles.

**Figure S60**. Frequency dependent rheological behavior of **MOC2G** displaying storage G′ and loss G″ moduli variation with angular frequency ($\omega$) over three self-healing cycles.

**Figure S61**. Amplitude sweep measurements of **MOC2G**, showing G′ and G″ moduli as a function of applied strain (γ) over three consecutive damage–healing cycles.

**Table S5**. Comparison of the quantitative recovery ratios of G′ and G″ by frequncy sweep (ω = 10 rad/s) and amplitude sweep (γ = 0.1%) during three consecutive damage−healing cycles.

**9**. **Static Adsorption of Iodine from Aqueous Media**

**Adsorption from an Aqueous Iodine Solution**:

To assess the iodine adsorption performance of the metal-organic cages (**MOC**s) in aqueous media, 12 mg of each MOC sample was divided equally into five vials (2 mg per vial). To each vial, 3 mL of a saturated aqueous iodine solution (1.2 mM) was added, and the suspensions were stirred at room temperature for predetermined time intervals. After adsorption, the mixtures were centrifuged to separate the solid and liquid phases. The residual iodine concentration in the supernatant was determined by UV−Vis spectroscopy using an external calibration curve. The iodine removal efficiency was subsequently calculated according to the following equation:

Removal efficiency = $\frac{C_{0}-C_{t}}{C_{0}} \times100$……………………. (Eq.1)

where $C_{0}$(mM) and $C_{t}$(mM) denote the initial iodine concentration and the iodine concentration at time $t$, respectively, in the aqueous solution before and after contact with the cage material.

The amount of iodine adsorbed from the aqueous phase was calculated using the following expression:

Quantity of iodine uptake (*q*_t_) = $\frac{C_{0}-C_{t}}{m}\times v\times M$…………..…(Eq.2)

Here, $q_{t}$(mg g^−1^) denotes the amount of iodine adsorbed per unit mass of adsorbent at time $t$(min). $C_{0}$(mM) and $C_{t}$(mM) correspond to the initial iodine concentration and the concentration at time $t$, respectively, in the aqueous phase before and after contact with the cage. $M$(g mol^‒1^) represents the molecular weight of iodine, $v$(mL) is the volume of the iodine solution, and $m$(mg) is the mass of the cage employed in the adsorption experiment.

**Maximum Iodine Uptake from a Saturated Aqueous Iodine Solution**:

To reliably assess the iodine adsorption capacity of the cages, a control experiment was conducted to quantify iodine loss arising from volatilization. This approach enabled clear differentiation between iodine removed via adsorption by the cages and iodine lost through evaporation.

**Control Experiment**: A 10 mL aliquot of aqueous iodine solution ($C_{0}$ = 1.2 mM) was stirred at ambient temperature for 48 h in a sealed vial without any adsorbent. Afterward, the residual iodine concentration (𝐶_c_) was measured via UV−Vis spectroscopy. The amount of lost iodine due to volatilization ${(Q}_{C}$) was calculated using:

$$Q_{C}=\left( C_{0}-C_{c} \right)\times v\times M$$

where *v* is the volume of the solution, and *M* (g mol⁻^1^) is the molecular weight of iodine.

**Adsorption Experiment**: Each cage sample (15 mg) was dispersed in a round-bottom flask containing 200 mL of saturated aqueous iodine solution (1.2 mM) and stirred at ambient temperature for 48 h. Upon completion of the adsorption process, the cage material was recovered by filtration. The residual iodine concentration in the filtrate ($C_{f}$) was quantified by UV-Vis spectroscopy using an appropriate calibration curve. The apparent iodine uptake ($Q_{a}$) was then calculated as follows:

$$Q_{a}=\left( C_{0}-C_{f} \right)\times v\times M$$

**Adsorption Capacity**: The actual adsorption capacity per gram of cage material ($Q_{t}$)^10^ was determined by correcting for iodine volatilization:

$$Q_{t}=\frac{\left( Q_{a}-Q_{c} \right)}{m}\times M$$

Here, m denotes the mass of the adsorbent cage. This calculation accounts for iodine losses due to volatilization, thereby ensuring that the reported adsorption capacity corresponds exclusively to the amount of iodine irreversibly retained by the solid adsorbent.

**Iodine Adsorption from Sea Water**:

A 200 mL aqueous seawater solution containing 1.2 mM iodine was placed in a 250 mL round bottom flask, and 15 mg of each MOC was added under continuous stirring. UV‒Vis absorption spectra were recorded at regular time intervals to monitor the adsorption process and determine the iodine uptake capacity as a function of time.

**Experiments to Assess Selective Adsorption**:

A known amount of each cage (3 mg) was added to an aqueous iodine solution (20 mL, 1.2 mM) containing 1–200 equivalents of competing salts (KF, KCl, KBr, KHCO_3_, K_2_SO_4_, or KNO_3_). The resulting suspensions were magnetically stirred at 25 °C for 60 min. After equilibration, the cages were separated by centrifugation, and the residual iodine concentration in the supernatant was quantified by UV–Vis spectroscopy. The iodine removal efficiency was calculated using Eq. 3. Control experiments were performed under identical conditions using iodine solution without added competing anions for comparison.

Distribution coefficient (*K*_d_) was calculated using the following Eq.3.^11^

Distribution coefficient (*K*_d_) $=\left( \frac{c_{0}-c_{t}}{c_{t}} \right)\times\frac{v}{m}$………….… (Eq.3)

Here, *K*_d_ denotes the distribution coefficient, where $C_{0}$ and $C_{t}$ correspond to the initial and equilibrium iodine concentrations in the aqueous phase, respectively. The parameter $v$ (mL) represents the solution volume, and $m$ (mg) is the mass of the adsorbent.

**Preparation of a 0.5 mM aqueous solution of I_3_^−^**:

Triiodide (I_3_^−^) solutions were freshly prepared prior to each experiment by dissolving iodine (I_2_, 21 mg) and potassium iodide (KI, 42 mg) in 100 mL of deionized water under continuous stirring. This procedure afforded an aqueous I_3_^−^ solution with a concentration of 0.50 mM, established by the equilibrium $I_{2}(\mathrm{aq})+I^{-}(\mathrm{aq})\rightleftharpoons I_{3}^{-}(\mathrm{aq})$( $K\approx783$).^12^

**Aqueous Phase Static Tri-iodide (I_2_/KI) Capture Studies**:

To determine the maximum iodine uptake capacity, 6 mg of each cage material was added to a vial containing potassium iodide (KI, 600 mg), iodine (I_2_, 300 mg), and deionized water (6 mL). The mixture was stirred at ambient temperature for 48 h to ensure saturation. After adsorption, the solid cage material was recovered by filtration, thoroughly washed with deionized water, and air-dried to afford a black solid. The filtrate was retained and analyzed by bisulfite-starch titration as described below to quantify the residual iodine concentration.^10,13^

**Bisulfite Starch Titration**:

An aliquot (5 mL) of a 1% (w/v) aqueous starch indicator solution was added to the combined filtrate and washings. The resulting solution was titrated dropwise with 0.05 M aqueous sodium bisulfite until complete disappearance of the characteristic blue coloration of the starch, iodine complex. The iodine adsorption capacity of the cage under investigation was subsequently calculated from the difference in iodine concentration in the aqueous phase before and after exposure to the cage material.

**Selectivity Test in Presence of Other Competing Ions**:

To evaluate the effect of competing anions on iodide uptake, 2 mg of each cage material was individually incubated with 3 mL of an I_3_^−^ solution (2.5 mM) containing 1–200 equivalents of a single competing anion (KF, KCl, KBr, KHCO₃, K₂SO₄, or KNO₃). The suspensions were stirred at ambient temperature for 60 min, after which the cages were separated by centrifugation. The residual I_3_^−^ concentration in the supernatant was quantified by UV-Vis spectrophotometry. Capture efficiencies in the presence of competing anions were determined relative to a control experiment performed with I_3_^−^ solution alone. The removal efficiency and distribution coefficient were calculated using Eq. 1 and 3, respectively.

**Isotherm Models of Iodine Adsorption**:

To elucidate the adsorption mechanism, the experimental adsorption data were analyzed using the Langmuir and Freundlich isotherm models.^[10]^ The Langmuir model, which assumes monolayer adsorption on a homogeneous surface, suggests that each adsorption site accommodates a single adsorbate molecule and that lateral interactions between adsorbed species are negligible. This model is described mathematically by Eq. 4

$\frac{Cⅇ}{q_{e}}=\frac{1}{k_{L}q_{m}}+\frac{Cⅇ}{q_{m}}$ ………………………………..(Eq.4)

where *k*_L_ (L mg^‒1^) is the Langmuir constant, and *q*_m_ (mg g^‒1^) is the maximum adsorption capacity.

The Freundlich model represents as Eq.5.

$\ln q_{e}=\ln k_{F}+\left( \frac{1}{n} \right)\ln c_{e}$…………………….. (Eq..5)

Where, *k*_F_ and (1/n) are the Freundlich model constant, indicating capacity and intensity of adsorption respectively.

**Figure S62**. (a) UV-Vis absorption spectra were recorded for aqueous iodine solutions across a range of concentrations. (b) A calibration curve was generated by plotting the absorbance at 460 nm as a function of concentration.

**Figure S63**. Time-dependent UV−Vis spectra monitoring the adsorption of iodine from water following the introduction of **MOC1**.

**Figure S64**. Time-resolved UV-Vis spectra of the aqueous iodine solution after the addition of **MOC2**.

**Figure S65**. The time-dependent decrease in aqueous iodine absorbance following treatment with **MOC1** and **MOC2**.

**Figure S66**. Time dependent adsorption of iodine by the cages from aqueous iodine solution.

**Figure S67**. Iodine removal efficiency from aqueous solution in the presence of the MOCs.

**Figure S68**. Linear fitting of the pseudo-second-order kinetic model for iodine adsorption from water by different cages.

**Table S6**. Pseudo second order kinetic parameters for the adsorption of iodine from aqueous solution

| Cage | *q*_e_ (mg g^‒1^)  (calculated) | *k*_2_  (g mg^‒1^ min^‒1^) | R^2^ |
| --- | --- | --- | --- |
| **MOC1** | 446.42 | 0.1463 | 0.99 |
| **MOC2** | 432.90 | 0.2198 | 0.99 |

**Figure S69**. UV–Vis absorption spectra showing the maximum iodine adsorption by the MOCs from aqueous solution.

**Figure S70**. Relative distribution coefficients (*K*_d_) for I_2_ in aqueous media containing 1-equivalent of various interfering anions in the presence of (a) **MOC1** and (b) **MOC2** cages.

**Figure S71**. (a) Langmuir and (b) Freundlich adsorption isotherms describing iodine uptake by **MOC1** materials from aqueous solution.

**Figure S72**. Iodine adsorption isotherms of cage **MOC2** in aqueous solution.

**Table S7**. Iodine adsorption isotherm parameters

| Cage | Langmuir isotherm model | | | Freundlich isotherm model | | |
| --- | --- | --- | --- | --- | --- | --- |
|  | *q*_m_  (mg g^‒1^) | *k*_L_  (L mg^‒1^) | R^2^ | *n* | *k*_F_  (L mg^‒1^) | R^2^ |
| **MOC1** | 469.00 | 0.1851 | 0.82 | 1.7353 | 1.65 | 0.93 |
| **MOC2** | 454.54 | 0.310 | 0.80 | 2.061 | 2.41 | 0.98 |

**Figure S73**. (a) UV–Vis absorption spectra of aqueous iodine solutions recorded at different concentrations. (b) Corresponding calibration curve constructed from the absorbance values measured at 352 nm.

**Figure S74**. Time-resolved UV–Vis absorption spectra of an aqueous I_3_^−^ solution in the presence of **MOC1**.

**Figure S75**. Progressive decrease in the UV–Vis absorbance at 352 nm of an aqueous I_3_^−^ solution upon addition of the MOCs.

**Figure S76**. I_3_^‒^ removal efficiency from aqueous solution in the presence of different **MOC**s.

**Figure S77**. Pseudo-second-order kinetic model fitting for the adsorption of aqueous triiodide (I_3_^−^) by the cages.

**Table S8**. Kinetic parameters for I_3_^−^ adsorption by the MOCs

| Cage | *q*_e_ (mg g⁻^1^)  (calculated) | *k*_2_  (g mg⁻^1^ min⁻^1^) | R^2^ |
| --- | --- | --- | --- |
| **MOC1** | 253.73 | 0.26 | 0.99 |
| **MOC2** | 263.85 | 0.56 | 0.99 |

**Figure S78**. Adsorption isotherm fitting of I_3_^−^ uptake by **MOC1** using the (a) Langmuir and (b) Freundlich models.

**Figure S79**. Adsorption isotherm fitting of I_3_^−^ uptake by **MOC2** using the (a) Langmuir and (b) Freundlich models.

**Table S9**. Isotherm parameters for the adsorption of I_3_^−^ by the cages

| Cage | Langmuir isotherm model | | | Freundlich isotherm model | | |
| --- | --- | --- | --- | --- | --- | --- |
|  | *q*_m_  (mg g^−1^) | *k*_L_  (L mg^−1^) | R^2^ | *n* | *k*_F_  (L mg^−1^) | R^2^ |
| **MOC1** | 345.16 | 0.073 | 0.82 | 1.02 | 33.76 | 0.92 |
| **MOC2** | 197.40 | 0.033 | 0.89 | 2.98 | 38.7 | 0.94 |

**Figure S80**. Adsorption of I_3_^−^ from aqueous solution by **MOC1** in the presence of individual competing anions (Br^−^, Cl^−^, F^−^, NO_3_^−^ HCO_3_^‒^, or SO_4_^2−^) added in excess (1–200 equivalents).

**Figure S81**. Relative distribution coefficients (*K*_d_) for I_3_^−^ measured in the presence of one equivalent of each competing anion.

**Figure S82**. I_3_^‒^ adsorption by **MOC1** at different pH in water.

**Figure S83**. pH-dependent I_3_^‒^ adsorption behavior of **MOC2** in the range of pH 4–9.

 **Figure S84**. Removal efficiency of I_3_^‒^ from aqueous solution at different pH values by the cages, (a) **MOC1** and (b) **MOC2**.

**Iodine adsorption by Gel**

**Figure S85**. Time-dependent aqueous iodine (1.2 mM) adsorption by the gel (a) **MOC1G** and (b) **MOC2G**.

**Figure S86**. Time-dependent (a) removal efficiency and (b) iodine uptake capacity of the MOCGs.

**Figure S87**. Pseudo-second-order kinetic model for iodine adsorption from water by the MOCGs.

**Figure S88**. Time-resolved I_3_^‒^ ($0.5\text{ mM}$) uptake by (a) **MOC1G** and (b) **MOC2G**.

**Figure S89**. Time-dependent (a) I_3_^‒^ removal efficiency and (b) I_3_^‒^ uptake capacity of the MOCGs from water.

**Figure S90**. Pseudo-second-order kinetic model for I_3_^‒^ adsorption from water by the MOCGs.

**Table SE10**. Comparison of the kinetics of iodine adsorption from aqueous media by the cages and their corresponding MOCGs.

**Figure S91**. Iodide (I_3_^−^) adsorption selectivity by **MOC1G** and **MOC2G** in aqueous media containing a 100-fold excess of individual co-existing anions (Br^−^, Cl^−^, F^−^, NO_3_^−^ HCO_3_^‒^, or SO_4_^2−^).

**Iodine Release in Methanol**:

For solution-phase iodine release experiments, ⁓2.0 mg of the I_2_-loaded cages, obtained via aqueous-phase adsorption, were dispersed in 3 mL of methanol. The release of iodine was monitored by recording the time-dependent increase in UV-Vis absorbance at 445 nm.

**Figure S92**. (a) Time-resolved UV–Vis absorption spectra showing the release of I_2_ from I_2_@**MOC1** in methanol at different intervals. (b) Absorbance at 445 nm monitoring I_2_ release over various points of time.

**Figure S93**. (a) Time-resolved UV–Vis absorption spectra showing the release of I_2_ from I_2_@**MOC2** in methanol at different intervals. (b) Absorbance at 445 nm following I_2_ release over various time points.

**10**. **Dynamic Adsorption of Iodine from Aqueous Solution**

To evaluate the dynamic capture efficiency of I_2_ and I_3_^−^ by the cage material, a continuous-flow column adsorption experiments were carried out. The cage material (10 mg) was dried and packed into a column and immobilized between two layers of degreased glass wool. An aqueous I_3_^−^ solution (0.5 mM) and I_2_ solution (1.2 mM) were separately passed through the column at a controlled flow rate using a water-flow regulator. The effluent was collected in 1 mL microcuvettes at predetermined intervals and analyzed by UV−Vis spectroscopy to quantify residual iodine species. The breakthrough volume was determined from the breakthrough point observed during continuous flow, providing a measure of the dynamic adsorption performance of the material.^14^

**Figure S94**. Changes in absorption of the first 1 mL fraction of aqueous I_2_ solution collected after passing through columns packed with MOCs.

**Figure S95**. UV–Vis absorption changes of the first 1 mL fraction of aqueous I_3_^−^ solution collected after passing through columns packed with the MOCs.

**Figure S96**. (a) UV–Vis absorption spectra of the effluent collected during the breakthrough experiment using **MOC1**. (b) Corresponding breakthrough curve for I_3_^−^ adsorption by **MOC1**.

**11**. **Adsorption of Trace Aqueous Iodine**

For the determination of low-concentration iodine and iodide residues in seawater and drinking water, the leuco-crystal violet (LCV) method was adopted.^15^ The specific protocols for reagent preparation and spectrophotometric analysis are described below.

**Scheme S7**. Mechanism of the Leuco-crystal violet method for determining I_2_/I^−^ residues.

**Preparation of Standard Iodide Solution**: A stock solution of 100 ppm iodide (I^−^) was prepared by dissolving 13.01 mg of potassium iodide in deionized water and diluting the solution to a final volume of 100 mL. Working standard solutions in the concentration range of 20−100 ppb were subsequently prepared by serial dilution of the stock solution and used for calibration curve construction

**Preparation of Phosphate Buffer Solution**: A phosphate buffer solution (100 mL, pH 4.0) was prepared by dissolving potassium dihydrogen phosphate (KH_2_PO_4_, 3.40 g) in ⁓30 mL of distilled water in a 100 mL volumetric flask. To adjust the pH, 2 mL of 85% phosphoric acid was added, followed by dilution to a final volume of 100 mL with distilled water.

**Preparation of N-Chlorosuccinimide-Succinimide Solution**: A 100 mL solution was prepared by dissolving N-chlorosuccinimide (100 mg, 0.75 mmol) and succinimide (1.0 g, 10.1 mmol) in distilled water in a 100 mL volumetric flask, followed by dilution to the mark with distilled water.

**Preparation of Leuco-Crystal Violet Solution**: A 100 mL leuco-crystal violet solution was prepared by dissolving 4,4',4"-methylidynetris (N, N-dimethylaniline) (25 mg, 0.067 mmol) in ⁓30 mL of distilled water in a 100 mL volumetric flask. Subsequently, 0.25 mL of 85% phosphoric acid was added, and the mixture was vigorously shaken until complete dissolution was achieved. The solution was then diluted to the mark with distilled water.

**Residual Concentration of** **[I_2_ + I^−^] Measurement**: To quantify the residual concentration of total iodine species ([I_2_ + I^−^]) in seawater, distilled water, and mixed-anion solutions, a 25 mL aliquot of the iodinated sample was combined with 1 mL of phosphate buffer and 1 mL of N-chlorosuccinimide−succinimide reagent in an appropriate vessel. Subsequently, 1 mL of leuco-crystal violet solution was added, and the mixture was thoroughly mixed. The absorbance was recorded at 591 nm using a UV−Vis spectrophotometer at 25 °C, precisely 20 min after the addition of leuco-crystal violet. The residual [I_2_ + I^−^] concentration was determined from a pre-established calibration curve.

**8**. **Investigation of the Absorption Mechanism**

**FT-IR Analysis**:

**Figure S97**. Comparative IR spectra of (a) **MOC1** and (b) **MOC2** recorded before and after iodine adsorption.

**TGA Analysis**:

**Figure S98**. Thermogravimetric analysis of the pristine cage (green) and the iodine-loaded cage I_2_@**MOC** (red).

**PXRD Analysis**:

**Figure S99**. Powder X-ray diffraction (PXRD) analysis of the (a) **MOC1** and (b) **MOC2** before and after iodine adsorption.

**FE-SEM Image**:

**Figure S100**. SEM image of MOCs post‑iodine adsorption.

**EDAX Analysis and EDS Mapping**:

**Figure S101**. EDAX elemental analysis of **MOC1** (top) and the corresponding EDS elemental mapping images (bottom).

**Figure S102**. Energy-dispersive X-ray spectroscopy (EDAX) analysis of **MOC2**, showing the elemental spectrum (top) and corresponding elemental maps (bottom).

**XPS Analysis**:

**Figure S103**. High‑resolution XPS spectra of C 1s of prestine **MOC1** and I_2_@**MOC1**.

**Figure S104**. XPS I 3d core-level spectra of I_2_@**MOC2**.

**Figure S105**. ^1^H NMR spectra of **MOC1** after iodine release.

**Figure S106**. ^1^H NMR spectra of **MOC2** after iodine release.

**19**. **DFT Calculations**

**Computational Details**.

All structures were fully optimised using the B3LYP functional^16,17^ as implemented in Gaussian 16.^18^ The LANL2DZ effective core potential and corresponding basis sets were applied to Pd and I atoms,^19,20^ while the 6-31+G basis set^21,22^ was used for all other atoms. Grimme’s D3 dispersion correction^23^ was included to account for van der Waals interactions.

**Figure S107**. Optimized structures of (a) **MOC1** and (b) **MOC2** (Color code: C-grey, N-blue, H-white, Pd-green), along with the corresponding electrostatic potential (ESP) distributions.

**Figure S108**. DFT calculations of the binding energies (kcal mol^−1^) for **MOC1** with I_2_ at various binding sites, along with the corresponding electrostatic potential (ESP) distributions.

**Figure S109**. Calculations of binding energies of the I_3_^−^ ion interacting with **MOC1** across various binding sites, along with the corresponding ESP.

**Figure S110**. DFT calculations of the binding energies (kcal mol^−1^) for **MOC2** with I_2_ at various binding sites, along with the corresponding electrostatic potential (ESP) distributions.

**Figure S111**. Calculations of binding energies of the I_3_^−^ ion interacting with **MOC2** across various binding sites, along with the corresponding ESP.

**14**. **Iodine Adsorption Capacities of Selected Materials in Aqueous Solution**

**Table S11**. Comparison of aqueous iodine adsorption capacities among various materials.

| Material | I_2_/ I_3_^−^ | Uptake capacity  (g g^−1^) | Materials  type | Reference |
| --- | --- | --- | --- | --- |
| **MOC2** | **I_2_/KI** | **3.01** | **MOC** | **This Work** |
| **MOC1** | **I_2_/KI** | **2.56** | **MOC** | **This Work** |
| **MOC2** | **I_2_** | **2.45** | **MOC** | **This Work** |
| **MOC1** | **I_2_** | **2.07** | **MOC** | **This Work** |
| Phen-TPA | I_2_/KI | 11.9 | COF | *Adv. Sci.* **2025***, 12,* e00697 |
| SUPE-py-Imine-Cage | I_2_/KI | 9.01 | NAS | *Nat. Commun*.  **2023**, *14*, 5388. |
| IPcomp-7 | I_2_/KI | 5.16 | Hybrid composite | *Nat. Commun*.  **2024***,* *15*, 1278 |
| COFP–1 | I_2_ | 5.05 | COF | *Chem. Mater*.  **2022**, *34*, 11062–11071 |
| Impyr-HCl | I_2_/KI | 4.22 | COF | *Small*  **2024**, *20*, 2404994 |
| C[4]P-BTP | I_2_/KI | 3.24 | POP | *Angew. Chem. Int. Ed*.  **2022** *61*, e202113724 |
| HcOF-4 | I_2_/KI | 3.6 | HOF | *J. Am. Chem. Soc*.  **2019**, *141*, 10915-10923 |
| CaCOP3 | I_2_ | 3.1 | POP | *Mater. Chem. Phys*.  **2020**, *239*, 122328 |
| MC2 | I_2_ | 2.73 | MOC | *Small* **2025,** *21***,** 2504242 |
| CalCOP1 | I_2_/KI | 2.32 | COP | *J. Mater. Sci*.  **2020**, *55*, 1854–1864 |
| MeO–CTF600 | I_2_/KI | 2.21 | COF | ACS Appl. Nano Mater.  **2022**, *5*, 8783-8793 |
| HCOFs–1 | I_2_/KI | 2.1 | COF | *J. Am. Chem. Soc*.  **2017**, *139*, 7172-7175 |
| NapC | I_2_/KI | 1.99 | MOC | *Chem. Eng. J*. **2024**, *496*, 154091 |
| TIEPE-DABCO | I_2_/KI | 1.8 | XOF | Small  **2023**, *19*, 2302902 |
| BC@Dopa–ZIF | I_2_/KI | 1.31 | MOF | *Cryst. Growth Des*.  **2017**, *18*, 356-363 |
| 3D MOF-1 | I_2_/KI | 1.10 | MOF | *ACS Appl. Mater. Interfaces* **2020**, *12*, 46107-46118 |
| MBM-MOF | I_2_/KI | 0.88 | MOF | *Angew. Chem. Int. Ed*.  **2018**, *57*, 10148-10152 |
| G-QP6 | I_2_/KI | 0.247 | POP | *Angew. Chem. Int. Ed*.  **2019**, *58*, 3885 |
| Cd(II)- triazole MOF | I_2_/KI | 0.18 | MOF | *Chem. Commun*.  **2011**, *47*, 7185-7187 |

**15**. **Iodine Adsorption Kinetics across Various Adsorbents**

**Table S12**. Comparative adsorption kinetics of triiodide (I_3_^−^) ions on different adsorbent materials.

| Materials | *k*_2_  (g mg^−1^ min^−1^) | Time (min) | Material type | Reference |
| --- | --- | --- | --- | --- |
| **MOC2** | **5.6×10^−1^** | **4 min** | **MOC** | **This Work** |
| **MOC1** | **2.6×10^−1^** | **4 min** | **MOC** | **This Work** |
| NapC | 0.84 × 10^−3^ | 60 | MOC | *Chem. Eng. J.,* **2024***,496* 154091 |
| Se_4_Me-PF_6_ | 2.10 × 10^−3^ | 120 | tetrapodal receptor | *ChemSusChem,* **2025***,*  *18,* e202500751 |
| MC2 | 4.25×10^−1^ | 4 | MOC | *Small* **2025**, *21*, 2504242 |
| OH-ExR4 | 1.18×10^−2^ | 27 | Supramolecular macrocycle | *Angew. Chem., Int. Ed*. **2024**, *63*, e202411261 |
| MOF nanosheets | 2.20×10^−3^ | 60 | MOF | *Inorg. Chem*.  **2022**, *61*, 13883–13892. |
| C[4]P-BTP | 7.81×10^−3^ | 10 | POP | *Angew. Chem., Int. Ed*.  **2022** *61*, e202113724 |
| LFC–K | 1.90×10^−3^ | 15 | ACM | *Ind. Crops Prod*.  **2021**, *169*, 113649 |
| PEG hydrogel | 1.50×10^−3^ | 270 | Others | *ACS Appl. Mater.*  *Interfaces* **2023**, *15*, 42942–42953 |
| TIEPE-DABCO | 6.7×10^−4^ | 300 | XOF | *Small*  **2023**, *19*, 2302902 |
| PTIBBL | 6.19×10^−4^ | 270 | POP | *Chem. Commun*.  **2020**, *56*, 1401–1404 |
| PP–3 | 5.90×10^−4^ | 30 | POP | J. Mater. Chem. A  **2022**, *10*, 20090–20100. |
| SUPE-py-Imine-Cage | 4.67×10^−4^ | 30 | NAS | *Nat. Commun*.  **2023**, *14*, 5388. |

**16**. **Photocatalytic Oxidation Reaction**

**Electrochemical Measurement**:

Electrochemical measurements were performed using an autolab 302 N electrochemical station in a standard, undivided three-electrode configuration. For Mott–Schottky analysis, electrochemical impedance spectroscopy (Nyquist plots), transient photocurrent response, and open-circuit potential measurements, working electrodes were prepared by dispersing 5 mg of the solid MOC powder in 240 μL of water via vortex mixing, followed by drop-casting the suspension onto fluorine-doped tin oxide (FTO) substrates and drying in a desiccator. Degassed aqueous solution containing Na_2_SO_4_ (200 mM) was used as the electrolyte. An Ag/AgCl (3.5 M KCl) electrode and a platinum wire served as the reference and counter electrodes, respectively. Photocurrent response data were analyzed and fitted using Fityk 1.3.1 software.^24^

**General Procedure for the Transformation of Thioether to Sulfoxide**:

The substrate (1.0 mmol) and catalyst MOC (0.5 mol %) was mixed in an oven-dried borosilicate reaction tube containing a magnetic stir bar. Methanol (1.0 mL) was added as reaction solvent. The reaction mixture was purged with oxygen for 2 min and irradiated with blue light (λ_max_ = 390 nm) using a Kessil PR160L 390 lamp positioned 15−20 cm from the vessel without additional optical filter. The reaction was stirred continuously at ambient temperature (25 °C) for 6 h. The temperature was maintained by fan-assisted cooling and monitored periodically. After completion, the crude mixture was purified via column chromatography (hexane/ethyl acetate, 4:2 v/v) to afford the desired products, which were characterized by ^1^H and ^13^C NMR spectroscopy. Reaction yields and selectivities were determined directly from the crude reaction mixture by ^1^H NMR analysis in CD_3_OD without prior purification.

**Light On-Off Experiment**:

An oven-dried NMR tube equipped with a miniature magnetic stir bar was charged with thioanisole (1.0 mmol, 10 mg), **MOC2** (0.5 mol%, 0.735 mg), and CD_3_OD (500 μL). Mesitylene (1.0 mmol) was added as an internal standard. The reaction mixture was purged with O_2_ for 2 min, sealed, and placed 20 cm away from a 390 nm blue LED light source (Kessil PR160L). The reaction was carried out at ambient temperature under continuous irradiation. Reaction progress was monitored by ^1^H NMR spectroscopy at regular intervals during alternating light−dark cycles, and the conversion and product selectivity were plotted as a function of time to evaluate the photocontrolled nature of the catalytic process.

**EPR Experiment**:

A 15 mL reaction vial was charged with the **MOC2** catalyst (0.5 mol%) and methanol (1 mL), followed by the addition of TEMP (3 equiv.) as a singlet oxygen trapping agent. The mixture was stirred under blue LED irradiation for 20 min, after which electron paramagnetic resonance (EPR) spectroscopy was performed. The EPR spectrum displayed three characteristic signals corresponding to the TEMPO radical, unambiguously confirming the generation of singlet oxygen (^1^O_2_) by cage during photocatalysis.^25^

**Figure S112**. Nyquist plot of **MOC1** and **MOC2**.

**Figure S113**. LSV data of **MOC1** and **MOC2**.

**Figure S114**. Fluorescence emission spectra of the MOCs upon excitation at 300 nm.

**Figure S115**. DFT-calculated HOMO and LUMO energy levels and electronic band gap of **MOC1**.

**Figure S116**. Calculated HOMO–LUMO energy levels and band gap of the **MOC2**.

**Figure S117**. Optimization of the photocatalytic aerobic oxidation of MPS mediated by **MOC2** under various reaction conditions.

**Control Experiment**:

A 15 mL reaction vial was charged with thioanisole (0.5 mmol) in MeOH and purged with oxygen for 2 min. The mixture was stirred at room temperature under blue-light irradiation (λ_max_ = 390 nm) for 6 h. Upon completion, product formation was quantified by using ^1^H NMR spectroscopy. In the absence of the MOC catalyst, methyl phenyl sulfoxide was obtained in 19% yield, accompanied by ⁓3% formation of the corresponding sulfone.

**Procedure for Radical Quenching Experiment**:

A 15 mL reaction vial was charged with thioanisole (1.0 mmol) and the **MOC2** catalyst (0.05 mol%). MeOH and different quencher (3 equiv.) were then added, and the reaction mixture was purged with oxygen for 2 min. The mixture was then stirred under blue LED irradiation for 6 h. The product yield was calculated by ^1^H NMR spectroscopy.

**Figure S118**. ^1^H NMR (400 MHz, 298 K) spectra of the photocatalytic oxidation of thioanisole by **MOC2** in the presence of different quenchers.

**Figure S119**. Comparison of the yields of thioanisole oxidation catalyzed by **MOC2** in the presence of different quenchers.

**17**. **Reusability of the Photocatalytic Material**

A 15 mL reaction flask was charged with MOC (5 mg) and thioanisole dissolved in methanol (5 mL). The reaction mixture was purged with O_2_ for 2 min and stirred under blue-light irradiation for 6 h at ambient temperature. Upon completion, methanol was removed under reduced pressure using a rotary evaporator, and the reaction yield was determined by ^1^H NMR spectroscopy. The recovered MOC catalyst was subsequently washed thoroughly with chloroform, dried under vacuum, and reused directly in subsequent cycles. This recycling procedure was repeated for five consecutive runs, with the fifth cycle still affording a high product yield of ⁓84% for **MOC1** and ⁓87% for **MOC2**, demonstrating the excellent recyclability and stability of the catalyst.^26^

**18**. **Fluorescence Quenching Experiment**

A dilute solution of the MOC in DMSO was prepared, and increasing concentrations of the quencher (thioanisole) were incrementally added. Steady-state fluorescence measurements were conducted in a 10 mm path-length, 3 mL screw-cap quartz cuvette with excitation at 300 nm.

The Stern-Volmer equation (I_0_/I) = 1 + *K_sv_*[Q], is fitted with the two quenching experiments. Here, (I_0_/I) is the ratio of fluorescence intensity in the absence and the presence of a quencher, *K_sv_* and [Q] are the quenching constant and quencher concentration, respectively. From the slope of the above two quenching experiments, it was calculated that *K_sv_* values are 350.8 M^−1^ [for **MOC1** with (thioanisole)], and 530.6 M^−1^ [**MOC2** with (thioanisole)]. *K_sv_* = *k_q_* × τ°, where *k_q_* and τ° are the quenching rate and lifetime (7.52 ns for **MOC1**, and 8.31 ns for **MOC2**) of the photosensitizer, respectively. So, the quenching rate *k_q_* is calculated to be 4.79 × 10^10^ M^−1^s^−1^ [for **MOC1** with (thioanisole)], and 6.38 × 10^10^ M^−1^s^−1^ [**MOC2** with (thioanisole)].^24^

**Figure S120**. Fluorescence quenching of (a) **MOC1** and (b) **MOC2** upon addition of thioanisole.

**19**. **Time-Dependent Reaction Kinetics and Selectivity**

An oven-dried NMR tube equipped with a miniature magnetic stir bar was charged with thioether (1.0 mmol), MOC (0.5 mol%), and CD_3_OD (500 μL). Mesitylene (1.0 mmol) was added as an internal standard. The reaction mixture was purged with O_2_ for 2 min, sealed, and positioned 20 cm from a 390 nm blue LED source (Kessil PR160L). The reaction was conducted at ambient temperature under continuous irradiation. Reaction progress was monitored periodically by ^1^H NMR spectroscopy, and and the conversion and product selectivity were determined and plotted as a function of time.

**Figure S121**. Time-resolved ^1^H NMR spectra monitoring the photocatalytic oxidation of thioanisole (**1**) by **MOC2**.

**Figure S122**. Kinetic plots for the selective aerobic oxidation of thioether by **MOC2**.

**Figure S123**. ^1^H NMR spectra tracking the photocatalytic oxidation of ethyl phenyl sulfide (**2**) over time by **MOC2**.

**Figure S124**. Time-dependent conversion and selectivity profile for the photocatalytic oxidation of ethyl phenyl sulfide (**2**) by **MOC2**.

**Figure S125**. Reaction progress monitored by time-resolved ^1^H NMR spectroscopy during the photocatalytic oxidation of methyl *p*-tolyl sulfide (**3**) by **MOC2**.

**Figure S126**. Conversion and selectivity as a function of time for the photocatalytic oxidation of methyl *p*-tolyl sulfide (**3**) using **MOC2**.

**Figure S127**. ^1^H NMR spectra tracking the photocatalytic oxidation of 4-methoxythioanisole (**4**) over time by **MOC2**.

**Figure S128**. Conversion and selectivity as a function of time for the photocatalytic oxidation of 4-methoxythioanisole (**4**) by **MOC2**.

**Figure S129**. Time-resolved ^1^H NMR spectra monitoring the photocatalytic oxidation of 4-fluorothioanisole (**5**) by **MOC2**.

**Figure S130**. Time-dependent conversion and selectivity plot for the photocatalytic oxidation of 4-fluorothioanisole (**5**) by **MOC2**.

**Catalytic Activity of Gel**

**Figure S131**. ^1^H NMR spectra of the reaction mixture (CDCl_3_, 400 MHz, 298 K) obtained from the photocatalytic oxidation of thioanisole (**1**) using the metallogels **MOC1G** and **MOC2G**.

**Figure S132**. ^1^H NMR spectra (CDCl_3_, 400 MHz, 298 K) of the reaction mixture obtained from photocatalytic oxidation of ethyl(phenyl)sulfane (**2**) using **MOC1G** and **MOC2G**.

**Figure S133**. ^1^H NMR spectra (CDCl_3_, 400 MHz, 298 K) of the reaction mixture obtained from oxidation of methyl(p-tolyl)sulfane (**3**) using **MOC1G** and **MOC2G**.

**Catalytic Heterogeneity test**:

**Figure S134**. Solubility assesment of **MOC1** across various solvents analyzed by ^1^H NMR (400 MHz, 298K).

.

**Figure S135**. Solubility assessment of **MOC2** in different solvents analyzed by ^1^H NMR spectroscopy (400 MHz, 298K).

**Figure S136**. Time-resolved ^1^H NMR spectra monitoring the reaction progress of thioanisole oxidation by **MOC2**, before and after the hot filtration at 160 min.

**Figure S137**. Hot filtration test comparing the reaction progress of thioanisole oxidation catalyzed by **MOC2** before filtration (red) and after hot filtration following removal of the cage (black)

**Figure S138**. ${}^{1}\text{H}$ NMR spectrum recorded in DMSO-*d*_6_ (400 MHz, 298K) of the reaction mixture after hot filtration, confirming the absence of the **MOC1** catalyst.

**Figure S139**. ${}^{1}\text{H}$ NMR spectrum recorded in DMSO-*d*_6_ (400 MHz, 298K) of the reaction mixture after hot filtration, confirming the absence of the **MOC2** catalyst.

**Table S13**. ICP-OES analysis of metal leaching from the cages under the reaction conditions and in various solvents.

**Figure S140**. Visual comparison of the reaction mixture in methanol before and after the reaction, demonstrating that the catalyst remains suspended throughout the reaction.

**20**. **Comparison of TOFs for Photocatalytic Oxidation Catalysts**

**Table S14**. Comparative analysis of turnover frequencies (TOFs) for the photocatalytic oxidation of methyl phenyl sulfide to methyl phenyl sulfoxide using different catalytic systems.

| Entry | Photocatalyst | Light source | TOF (h^−1^) | Reference |
| --- | --- | --- | --- | --- |
| 1 | **MOC2** | **Blue LED 390 nm** | **29.47** | **This work** |
| 2 | **MOC1** | **Blue LED 390 nm** | **27.93** | **This work** |
| 3 | BTT-sp^2^c-COF | Grenn LED (520 nm, 3 W×4) | 42.6 | *Sci. China Chem*. **2023**, *66*, 3290. |
| 4 | TBPA-COF | Blue LED (460±10 nm, 3 W×4) | 38.9 | *Chem. Eng. J*. **2023**, *451*, 138802. |
| 5 | Py-Π-AQ-CMP | Blue LED (460 nm) | 37.2 | *Mater. Today Energy* **2023**, *38*, 101443. |
| 6 | HOF-66 | Red LED (>630 nm, 25 mW/cm^2^) | 19.6 | *Nano Res*. **2023**, *16*, 8809. |
| 7 | Py-Azine-COF | Blue LED (460 nm) | 23.5 | *Appl. Catal. B Environ. Energy* **2024**, *345*, 123660. |
| 8 | 3P-Por-COF | Red LED (620 nm>λ>630nm,25 mW/cm^2^) | 271.1 | *Adv. Funct. Mater*. **2025**, *35*, 2415629 |
| 9 | TTO-COF | Blue LED (3 W×4) | 12.4 | *Appl. Catal. B Environ*. **2022**, *305*, 121027. |
| 10 | CTF-PTZ | Blue LED (460±10 nm, 3 W×4) | 8.9 | *J. Mater. Chem. A* **2022**, *10*, 12489. |
| 11 | BCMP | Blue LED (100 W) | 4.3 | *Green Chem*. **2021**, *23*, 7945. |
| 12 | Tp-Bpy-COF | Blue LED | 1.5 | *Adv. Funct. Mater*. **2025**, *35*, 2413943. |

**21**. **Conversion Efficiency Comparison for Photocatalytic Sulfoxidation**

**Table 15**. Comparative evaluation of conversion efficiencies for the photocatalytic oxidation of methyl phenyl sulfide to methyl phenyl sulfoxide over different catalytic systems.

| Entry | Photocatalyst | Light source | Conversion efficiency  (mmol g^−1^ h^−1^) | Refences |
| --- | --- | --- | --- | --- |
| 1 | **MOC2** | **Blue LED 390 nm** | **16.17** | **This work** |
| 2 | **MOC1** | **Blue LED 390 nm** | **15.62** | **This work** |
| 2 | TPPS-BV | Red LED (620 nm>λ>630nm,25 mW/cm^2^) | 10 | *Adv. Sci.* **2024**, 11, 2308469. |
| 3 | NNU-45/Anthracene | Visible Light, Xe lamp (300W) | 16.7 | *ACS Appl. Mater*. *Interfaces* **2019**, *11*, 3016. |
| 4 | Zr_6_-Irphen/ Irphan | Blue LED (100 W, 460 nm) | 4.2 | *Appl. Mater. Interfaces* **2019**, *11*, 41448. |
| 5 | Compound 1 | 10 W LED (405 nm) | 33.3 | *Inorg. Chem*. **2023**, *62*, 10120. |
| 6 | ARS-Ti-MCM-48 | Green LED | 16.66 | *Chem. Eng. J*. **2022**, *432*, 134285. |
| 7 | In2S3/NU-1000-3 | LED lamp irradiation | 4.7 | *Chem. Eur. J*. **2022**, *28*, e202103466. |
| 8 | CoMo-TPT | 10 W white LED lamps | 4.014 | *Inorg. Chem*. **2021**, *60*, 16810 |
| 9 | ARS-TiO_2_/SiO_2_ | Blue LED (460±10 nm, 3 W×4) | 11.9 | *Chem. Eng. J*. **2021**, *423*, 129419 |
| 10 | ARS-Nb2O5 | Green LED (520±10 nm, 3 W×4) | 7.158 | *J. Mater. Chem. A* **2021**, *9*, 2214. |

**Figure S141**. ^1^H NMR spectra of **MOC1** after five catalytic cycle.

**Figure S142**. ^1^H NMR spectra of **MOC2** after five catalytic cycle.

**Figure S143**. Full XPS survey spectra of (a) **MOC1** and (b) **MOC2** before and after photocatalytic oxidation.

**Figure S144**. Pd 3d XPS spectra of (a) **MOC1** and (b) **MOC2** before and after photocatalytic oxidation

**22**. **Comparison of Reported Metallocage Based Gels and their Applications**

**Table 16**. Comparison of reported metallocage-based supramolecular gels their application.

| Entry | Mettalogel | Application | Reference |
| --- | --- | --- | --- |
| **1** | **MOC1G and MOC2G** | **Self-Healling gel, iodine adsorption, and heterogenous phtotcatalyst** | **This work** |
| 2 | **O-Pd_2_L_4_** cage Gel | Light responsive and smart soft materials | *Chem. Eur. J*. **2015**, *21*, 7418 – 7427 |
| 3 | **PdG** | Anti-cancer drug to  Melanoma Cell B16‒ F10 | *Chem Asian J.* **2024**, *19*, e202400419 |
| 4 | Complex **2a** Xerogel | Multiple stimuli-responsive  properties | *Dalton Trans*., **2015**, *44*, 15181–15188 |
| 5 | **NGel 1** | Sorbent for chloride ions in water and drug delivery | *Angew. Chem. Int. Ed.* **2024**, *63*, e202403834. |
| 6 | **MOC@PILs** | Removal of pollutants from water and for the purification of toluene and cyclohexane | *Nat Commun.,* **2024***,* *15*, 3766 |
| 7 | **1**⊂**Gel** and **2**⊂**Gel** | Separation of small molecules | *Angew. Chem. Int. Ed.* **2019**, *58*, 7982. |
| 8 | **G^NH2^**, **G^Me^**, **G^Pr^** and **G^Hept^** | Self-healing behavior and shows good adhesion to various surfaces | *Angew. Chem. Int. Ed.* **2022**, *61*, e202201793 |
| 9 | **S-G** | Adsorption of dyes (Crystal Violet and Rhodamine B) | *Dalton Trans.* **2023**, *52*, 4980–4984 |
| 10 | Complex **1** Gel | Stimuli-responsive, pyrene uptake and dye removal | *Inorg. Chem*. **2018**, *57*, 3634−3645 |

**22**. **Analytical data for synthesized products**

(methyl-sulfinyl) benzene (**1**)

^1^H NMR (700 MHz, DMSO-*d*_6_): *δ* (ppm) *=* 7.64 (dd, *J* = 7.8, 1.6 Hz, 2H), 7.55 – 7.47 (m, 3H), 2.72 (s, 3H). ^13^C NMR (176 MHz, DMSO-*d*_6_): *δ* (ppm) = 145.61, 131.22, 129.50, 123.65, 43.99. HRMS (ESI): C_7_H_8_OS, [M + H]^+^ = 141.20 (calculated) found: 141.201.

(ethyl-sulfinyl) benzene (**2**)

^1^H NMR (700 MHz, DMSO-*d*_6_): *δ* (ppm) *=* 77.62 (dd, *J* = 7.9, 1.7 Hz, 2H), 7.56 – 7.48 (m, 3H), 2.91 (dq, *J* = 14.9, 7.4 Hz, 1H), 2.83 – 2.73 (m, 1H), 1.21 (t, *J* = 7.4 Hz, 3H). ^13^C NMR (101 MHz, DMSO-*d*_6_): *δ* (ppm) = 143.32, 130.93, 129.14, 124.19, 50.30, 5.97. HRMS (ESI): C_8_H_10_OS, [M + H]^+^ = 155.23 (calculated) found: 155.231.

1-methyl-4-(methyl-sulfinyl) benzene (**3**)

^1^H NMR (400 MHz, DMSO-*d*_6_): *δ* (ppm) *=* 7.51 (d, *J* = 8.0 Hz, 2H), 7.30 (d, *J* = 7.9 Hz, 2H), 2.68 (s, 3H), 2.39 (s, 3H). ^13^C NMR (101 MHz, DMSO-*d*_6_): *δ* (ppm) = 142.42, 141.52, 130.03, 123.54, 43.97, 21.38. HRMS (ESI): C_8_H_10_OS, [M + H]^+^ = 155.23 (calculated) found: 155.23.

1-methoxy-4-(methyl-sulfinyl) benzene (**4**)

^1^H NMR (400 MHz, DMSO-*d*_6_): *δ* (ppm) *=* 7.58 (d, *J* = 8.7 Hz, 2H), 7.01 (d, *J* = 8.7 Hz, 2H), 3.83 (s, 3H), 2.70 (s, 3H). ^13^C NMR (101 MHz, DMSO-*d*_6_): *δ* (ppm) = 162.03, 136.08, 125.63, 114.90, 55.54, 43.69. HRMS (ESI): C_8_H_10_O_2_S, [M + H]^+^ = 171.23 (calculated) found: 171.228.

1-fluoro-4-(methyl-sulfinyl) benzene (**5**)

^1^H NMR (400 MHz, DMSO-*d*_6_): *δ* (ppm) *=* 7.69 – 7.61 (m, 2H), 7.22 (t, *J* = 8.6 Hz, 2H), 2.71 (s, 3H). ^13^C NMR (101 MHz, DMSO-*d*_6_): *δ* (ppm) = 165.60, 163.10, 141.17, 141.14, 125.90, 125.82, 116.83, 116.60, 44.16. HRMS (ESI): C_7_H_7_OFS, [M + H]^+^ = 159.19 (calculated) found: 159.192.

1-chloro-4-(methyl-sulfinyl) benzene (**6**)

^1^H NMR (400 MHz, DMSO-*d*_6_): *δ* (ppm) *=* 7.55 (d, *J* = 8.5 Hz, 2H), 7.46 (d, *J* = 8.6 Hz, 2H), 2.68 (s, 3H). ^13^C NMR (101 MHz, DMSO-*d*_6_): *δ* (ppm) = 144.24, 137.17, 129.63, 124.97, 44.02. HRMS (ESI): C_7_H_7_OClS, [M + H]^+^ = 175.64 (calculated) found: 175.647.

1-bromo-4-(methyl-sulfinyl) benzene (**7**)

^1^H NMR (400 MHz, DMSO-*d*_6_): *δ* (ppm) *=* 7.66 (d, *J* = 8.5 Hz, 2H), 7.51 (d, *J* = 8.5 Hz, 2H), 2.71 (s, 3H). ^13^C NMR (101 MHz, DMSO-*d*_6_): *δ* (ppm) = 144.84, 132.59, 125.49, 125.16, 43.98. HRMS (ESI): C_7_H_7_OBrS, [M + H]^+^ = 220.10 (calculated) found: 220.11.

4-(methyl-sulfinyl) benzaldehyde (8)

^1^H NMR (400 MHz, DMSO-*d*_6_): *δ* (ppm) *=* 10.02 (s, 1H), 7.98 (d, *J* = 8.3 Hz, 2H), 7.77 (d, *J* = 8.2 Hz, 2H), 2.74 (s, 3H). ^13^C NMR (101 MHz, DMSO-*d*_6_): *δ* (ppm) = 191.20, 152.22, 138.09, 130.38, 124.19, 43.61. HRMS (ESI): C_8_H_8_O_2_S, [M + H]^+^ = 169.21 (calculated) found: 169.213.

1-(methyl-sulfinyl)-4-nitrobenzene (**9**)

^1^H NMR (400 MHz, CDCl_3_): *δ* (ppm) *=* 8.42 (d, *J* = 8.8 Hz, 2H), 7.86 (d, *J* = 8.8 Hz, 2H), 2.82 (s, 3H). ^13^C NMR (101 MHz, DMSO-*d*_6_): *δ* (ppm) = 153.21, 149.55, 124.68, 124.52, 43.87. HRMS (ESI): C_7_H_7_NO_3_S, [M + H]^+^ = 186.20 (calculated) found: 186.203.

**Figure S145**. ^1^H NMR spectrum of (methyl-sulfinyl) benzene (**1**) in CDCl_3_ (400 MHz, 298 K).

**Figure S146**. ^13^C NMR spectrum of (methyl-sulfinyl) benzene (**1**) in CDCl_3_ (100 MHz, 298 K).

**Figure S147**. ^1^H NMR spectrum of (ethyl-sulfinyl) benzene (**2**) in CDCl_3_ (400 MHz, 298 K).

**Figure S148**. ^13^C NMR spectrum of (ethyl-sulfinyl) benzene (**2**) in CDCl_3_ (100 MHz, 298 K).

**Figure S149**. ^1^H NMR spectrum of 1-methyl-4-(methyl-sulfinyl) benzene (**3**) in CDCl_3_ (400 MHz, 298 K).

**Figure S150**. ^13^C NMR spectrum of 1-methyl-4-(methyl-sulfinyl) benzene (**3**) in CDCl_3_ (100 MHz, 298 K).

**Figure S151**. ^1^H NMR spectrum of 1-methoxy-4-(methyl-sulfinyl) benzene (**4**) in CDCl_3_ (400 MHz, 298 K).

**Figure S152**. ^13^C NMR spectrum of 1-methoxy-4-(methyl-sulfinyl) benzene (**4**) in CDCl_3_ (100 MHz, 298 K).

**Figure S153**. ^1^H NMR spectrum of 1-fluoro-4-(methyl-sulfinyl) benzene (**5**) in CDCl_3_ (400 MHz, 298 K).

**Figure S154**. ^13^C NMR spectrum of 1-fluoro-4-(methyl-sulfinyl) benzene (**5**) in CDCl_3_ (100 MHz, 298 K).

**Figure S155**. ^1^H NMR spectrum of 1-chloro-4-(methyl-sulfinyl) benzene (**6**) in CDCl_3_ (400 MHz, 298 K).

**Figure S156**. ^13^C NMR spectrum of 1-chloro-4-(methyl-sulfinyl) benzene (**6**) in CDCl_3_ (100 MHz, 298 K).

**Figure S157**. ^1^H NMR spectrum of 1-bromo-4-(methyl-sulfinyl) benzene (**7**) in CDCl_3_ (400 MHz, 298 K).

**Figure S158**. ^13^C NMR spectrum of 1-bromo-4-(methyl-sulfinyl) benzene (**7**) in CDCl_3_ (100 MHz, 298 K).

**Figure S159**. ^1^H NMR spectrum of 4-(methyl-sulfinyl) benzaldehyde (**8**) in CDCl_3_ (400 MHz, 298 K).

**Figure S160**. ^13^C NMR spectrum of 4-(methyl-sulfinyl) benzaldehyde (**8**) in CDCl_3_ (100 MHz, 298 K).

**Figure S161**. ^1^H NMR spectrum of 1-(methyl-sulfinyl)-4-nitrobenzene (**9**) in CDCl_3_ (400 MHz, 298 K).

**Figure S162**. ^13^C NMR spectrum of 1-(methyl-sulfinyl)-4-nitrobenzene (**9**) in CDCl_3_ (100 MHz, 298 K).

**24**. **References**

(1) A. Nakagawa; W. Ota; T. Ehara; Y. Matsuo; K. Miyata; K. Onda; T. Sato; S. Seki; T. Tanaka, "Synthesis of substituent-free dioxadiaza[8]circulene to investigate intermolecular interactions and photophysical properties" *Chemical Communications* 60, no. 99 (2024): 14770-14773 https://doi.org/10.1039/D4CC05539J.

(2) H. P. Zhou; P. Wang; Z. J. Hu; L. Li; J. J. Chen; Y. Cui; Y. P. Tian; J. Y. Wu; J. X. Yang; X. T. Tao; M. H. Jiang, "Synthesis, Crystal Structures, and Photoluminescence of a Series of Coordination Polymers with Two Homologous Functional Flexible Ligands" *European Journal of Inorganic Chemistry* 2007, no. 13 (2007): 1854-1866 https://doi.org/10.1002/ejic.200600887.

(3) S. Garain; A. A. Kongasseri; S. M. Wagalgave; R. Konar; D. Deb; K. S. Narayan; P. K. Samanta; S. J. George, "Supramolecular Charge‐Transfer Approach for Tunable and Efficient Circularly Polarized Delayed Fluorescence and Phosphorescence" *Angewandte Chemie International Edition* 64, no. 28 (2025): e202515218 https://doi.org/10.1002/anie.202501330.

(4) Y. Xie; Y. Gong; M. Han; F. Zhang; Q. Peng; G. Xie; Z. Li, "Tetraphenylcyclopentadiene-Based Hyperbranched Polymers: Convenient Syntheses from One Pot “A4 + B2” Polymerization and High External Quantum Yields up to 9.74% in OLED Devices" *Macromolecules* 52, no. 3 (2019): 896-903 https://doi.org/10.1021/acs.macromol.8b02051.

(5) O. V. Dolomanov; L. J. Bourhis; R. J. Gildea; J. A. K. Howard; H. Puschmann, "OLEX2: a complete structure solution, refinement and analysis program" *J. Appl. Crystallogr.* 42, no. 2 (2009): 339-341 https://doi.org/10.1107/s0021889808042726.

(6) G. M. Sheldrick, "A short history ofSHELX" *Acta Cryst. A* 64, no. 1 (2007): 112-122 https://doi.org/10.1107/s0108767307043930.

(7) G. M. Sheldrick, "SHELXT– Integrated space-group and crystal-structure determination" *Acta Cryst. A* 71, no. 1 (2015): 3-8 https://doi.org/10.1107/s2053273314026370.

(8) O. V. Dolomanov; L. J. Bourhis; R. J. Gildea; J. A. K. Howard; H. Puschmann, "OLEX2: a complete structure solution, refinement and analysis program" *Journal of Applied Crystallography* 42, no. 2 (2009): 339-341 https://doi.org/10.1107/s0021889808042726.

(9) C. Cheng; X. Zhang; Y. Meng; Z. Zhang; J. Chen; Q. Zhang, "Multiresponsive and biocompatible self-healing hydrogel: its facile synthesis in water, characterization and properties" *Soft Matter* 13, no. 16 (2017): 3003-3012 https://doi.org/10.1039/c7sm00350a.

(10) Z. Zheng; Q. Lin; L. Xie; X. Chen; H. Zhou; K. Lin; D. Zhang; X. Chi; J. L. Sessler; H. Wang, "Macrocycle polymeric networks based on a chair-like calix[4]pyrrole for the rapid and efficient adsorption of iodine from water" *J. Mater. Chem. A* 11, no. 25 (2023): 13399-13408 https://doi.org/10.1039/d2ta09474f.

(11) A. Sen; S. Sharma; S. Dutta; M. M. Shirolkar; G. K. Dam; S. Let; S. K. Ghosh, "Functionalized Ionic Porous Organic Polymers Exhibiting High Iodine Uptake from Both the Vapor and Aqueous Medium" *ACS Appl. Mater. Interfaces.* 13, no. 29 (2021): 34188-34196 https://doi.org/10.1021/acsami.1c07178.

(12) J. L. Pursell; C. J. Pursell, "Host–Guest Inclusion Complexation of α-Cyclodextrin and Triiodide Examined Using UV–Vis Spectrophotometry" *The Journal of Physical Chemistry A* 120, no. 13 (2016): 2144-2149 https://doi.org/10.1021/acs.jpca.6b00982.

(13) L. Xie; Z. Zheng; Q. Lin; H. Zhou; X. Ji; J. L. Sessler; H. Wang, "Calix[4]pyrrole‐based Crosslinked Polymer Networks for Highly Effective Iodine Adsorption from Water" *Angew. Chem. Int. Ed.* 61, no. 1 (2021): e202113724 https://doi.org/10.1002/anie.202113724.

(14) S. Saurabh; S. Mollick; Y. D. More; A. Banerjee; S. Fajal; N. Kumar; M. M. Shirolkar; S. B. Ogale; S. K. Ghosh, "Covalent Organic Framework Featuring High Iodine Uptake for Li-Ion Battery: Unlocking the Potential of Hazardous Waste" *ACS Materials Letters* 5, no. 9 (2023): 2422-2430 https://doi.org/10.1021/acsmaterialslett.3c00443.

(15) M. Zhang; J. Samanta; B. A. Atterberry; R. Staples; A. J. Rossini; C. Ke, "A Crosslinked Ionic Organic Framework for Efficient Iodine and Iodide Remediation in Water" *Angewandte Chemie International Edition* 61, no. 52 (2022): e202214189 https://doi.org/10.1002/anie.202214189.

(16) A. D. Becke, "Density-functional thermochemistry. III. The role of exact exchange" *The Journal of Chemical Physics* 98, no. 7 (1993): 5648-5652 https://doi.org/10.1063/1.464913.

(17) C. Lee; W. Yang; R. G. Parr, "Development of the Colle-Salvetti correlation-energy formula into a functional of the electron density" *Physical Review B* 37, no. 2 (1988): 785-789 https://doi.org/10.1103/PhysRevB.37.785.

(19) P. J. Hay; W. R. Wadt, "Ab initio effective core potentials for molecular calculations. Potentials for the transition metal atoms Sc to Hg" *The Journal of Chemical Physics* 82, no. 1 (1985): 270-283 https://doi.org/10.1063/1.448799.

(18) M. J. Frisch; G. W. Trucks; H. B. Schlegel; G. E. Scuseria; M. A. Robb; J. R. Cheeseman; G. Scalmani; V. Barone; G. A. Petersson; H. Nakatsuji; X. Li; M. Caricato; A. V. Marenich; J. Bloino; B. G. Janesko; R. Gomperts; B. Mennucci; H. P. Hratchian; J. V. Ortiz; A. F. Izmaylov; J. L. Sonnenberg; Williams; F. Ding; F. Lipparini; F. Egidi; J. Goings; B. Peng; A. Petrone; T. Henderson; D. Ranasinghe; V. G. Zakrzewski; J. Gao; N. Rega; G. Zheng; W. Liang; M. Hada; M. Ehara; K. Toyota; R. Fukuda; J. Hasegawa; M. Ishida; T. Nakajima; Y. Honda; O. Kitao; H. Nakai; T. Vreven; K. Throssell; J. A. Montgomery Jr.; J. E. Peralta; F. Ogliaro; M. J. Bearpark; J. J. Heyd; E. N. Brothers; K. N. Kudin; V. N. Staroverov; T. A. Keith; R. Kobayashi; J. Normand; K. Raghavachari; A. P. Rendell; J. C. Burant; S. S. Iyengar; J. Tomasi; M. Cossi; J. M. Millam; M. Klene; C. Adamo; R. Cammi; J. W. Ochterski; R. L. Martin; K. Morokuma; O. Farkas; J. B. Foresman; D. J. Fox *Gaussian 16 Rev. A.03*, Wallingford, CT, 2016.

(20) W. R. Wadt; P. J. Hay, "Ab initio effective core potentials for molecular calculations. Potentials for main group elements Na to Bi" *The Journal of Chemical Physics* 82, no. 1 (1985): 284-298 https://doi.org/10.1063/1.448800.

(21) R. Krishnan; J. S. Binkley; R. Seeger; J. A. Pople, "Self-consistent molecular orbital methods. XX. A basis set for correlated wave functions" *The Journal of Chemical Physics* 72, no. 1 (1980): 650-654 https://doi.org/10.1063/1.438955.

(22) T. Clark; J. Chandrasekhar; G. W. Spitznagel; P. V. R. Schleyer, "Efficient diffuse function‐augmented basis sets for anion calculations. III. The 3‐21+G basis set for first‐row elements, Li–F" *Journal of Computational Chemistry* 4, no. 3 (2004): 294-301 https://doi.org/10.1002/jcc.540040303.

(23) S. Grimme; J. Antony; S. Ehrlich; H. Krieg, "A consistent and accurateab initioparametrization of density functional dispersion correction (DFT-D) for the 94 elements H-Pu" *The Journal of Chemical Physics* 132, no. 15 (2010): 154104 https://doi.org/10.1063/1.3382344.

(24) A. Jati; S. Dam; T. S. Khan; B. Maji, "A Robust Nickel‐Interlocked π‐Conjugated Covalent Organic Framework Catalyst for Photocatalytic Aromatic Finkelstein and Retro‐Finkelstein Reactions" *Angewandte Chemie International Edition* 64, no. 36 (2025): e202510788 https://doi.org/10.1002/anie.202510788.

(25) J. Q. Chen; K. Y. Zhang; X. D. Zhang; Z. Q. Huang; H. Deng; Y. Zhao; Z. Z. Shi; W. Y. Sun, "A Green Environmental Protection Photocatalytic Molecular Reactor for Aerobic Oxidation of Sulfide to Sulfoxide" *Chemistry – A European Journal* 30, no. 12 (2024): e202303725 https://doi.org/10.1002/chem.202303725.

(26) B. Mishra; S. Maji; M. Roy; S. Dutta; A. Sinha; T. N. Das; S. Mondal; A. Banerjee; A. Thomas; P. Pachfule; D. Adhikari, "A Thiazole-Linked Dithiophenedione-Functionalized Covalent Organic Framework for Photocatalytic Oxidative Dehydrogenation Reactions" *Chemistry of Materials* 37, no. 21 (2025): 8775-8786 https://doi.org/10.1021/acs.chemmater.5c01745.
